# A travelling-wave strategy for plant–fungal trade

**DOI:** 10.1038/s41586-025-08614-x

**Published:** 2025-02-26

**Authors:** Loreto Oyarte Galvez, Corentin Bisot, Philippe Bourrianne, Rachael Cargill, Malin Klein, Marije van Son, Jaap van Krugten, Victor Caldas, Thomas Clerc, Kai-Kai Lin, Félix Kahane, Simon van Staalduine, Justin D. Stewart, Victoria Terry, Bianca Turcu, Sander van Otterdijk, Antoine Babu, Marko Kamp, Marco Seynen, Bas Steenbeek, Jan Zomerdijk, Evelina Tutucci, Merlin Sheldrake, Christophe Godin, Vasilis Kokkoris, Howard A. Stone, E. Toby Kiers, Thomas S. Shimizu

**Affiliations:** 1 Amsterdam Institute for Life and Environment, Vrije Universiteit, Amsterdam, The Netherlands; 2 AMOLF Institute, Amsterdam, The Netherlands; 3 Laboratoire Reproduction et Développement des Plantes, Univ Lyon, ENS de Lyon, UCB Lyon 1, CNRS, INRAE, INRIA, Lyon, France; 4 Department of Mechanical and Aerospace Engineering, Princeton University, Princeton, NJ USA; 5 PMMH, CNRS, ESPCI Paris, Université PSL, Sorbonne Université, Université Paris Cité, Paris, France; 6 Society for the Protection of Underground Networks, SPUN, Dover, DE USA

**Keywords:** Microbial ecology, Intracellular movement, Coevolution, Fungal biology, Biological physics

## Abstract

For nearly 450 million years, mycorrhizal fungi have constructed networks to collect and trade nutrient resources with plant roots^1,2^. Owing to their dependence on host-derived carbon, these fungi face conflicting trade-offs in building networks that balance construction costs against geographical coverage and long-distance resource transport to and from roots^3^. How they navigate these design challenges is unclear^4^. Here, to monitor the construction of living trade networks, we built a custom-designed robot for high-throughput time-lapse imaging that could track over 500,000 fungal nodes simultaneously. We then measured around 100,000 cytoplasmic flow trajectories inside the networks. We found that mycorrhizal fungi build networks as self-regulating travelling waves—pulses of growing tips pull an expanding wave of nutrient-absorbing mycelium, the density of which is self-regulated by fusion. This design offers a solution to conflicting trade demands because relatively small carbon investments fuel fungal range expansions beyond nutrient-depletion zones, fostering exploration for plant partners and nutrients. Over time, networks maintained highly constant transport efficiencies back to roots, while simultaneously adding loops that shorten paths to potential new trade partners. Fungi further enhance transport flux by both widening hyphal tubes and driving faster flows along ‘trunk routes’ of the network^5^. Our findings provide evidence that symbiotic fungi control network-level structure and flows to meet trade demands, and illuminate the design principles of a symbiotic supply-chain network shaped by millions of years of natural selection.

## Abstract (web summary)

Symbiotic fungi control network-level structure and flows to meet trade demands.

## Main

The arbuscular mycorrhizal (AM) symbiosis is arguably the most widespread symbiotic partnership in nature, forming in the roots of around 70% of terrestrial plant species^6^ that, in turn, dominate Earth’s biomass^7^. AM fungi form complex mycelial networks of filamentous hyphae that are aseptate—meaning that their cells are not divided by internal walls. They form open conduits where carbon and nutrients are stored, and also flow dynamically through cytoplasmic streaming toward and away from host roots^8^. These nutrient-rich networks can reach densities of 10 m cm^−3^ and underlie global carbon cycling^9,10^.

The diverse trade behaviours enacted by mycorrhizal fungi are well documented, with research suggesting that fungal partners move and exchange resources in ways that can improve their access to host carbon^11–13^. Although progress has been made in imaging mycorrhizal networks^14^ and exploring their cytoplasmic dynamics^8^, their precise topology—and internal cytoplasmic flows—have never been quantitatively tracked across space and time. Models of AM network growth have depended primarily on coarser mycelial density data^15^, which cannot resolve how AM fungi build and operate their networks to meet trade demands. This is surprising because the spatial and temporal context of resource movement is fundamental to AM symbioses: the fungal partner depends on plant roots for carbon, received as sugars and fats (that is, obligate biotroph). In return, the fungus must continuously provide nutrients (such as phosphorus) to the host by extracting and moving resources through filamentous networks. The spatial expansion of the fungal network leads to new opportunities for colonization and trade, as the network encounters new resources and roots.

**Fig. 1:**
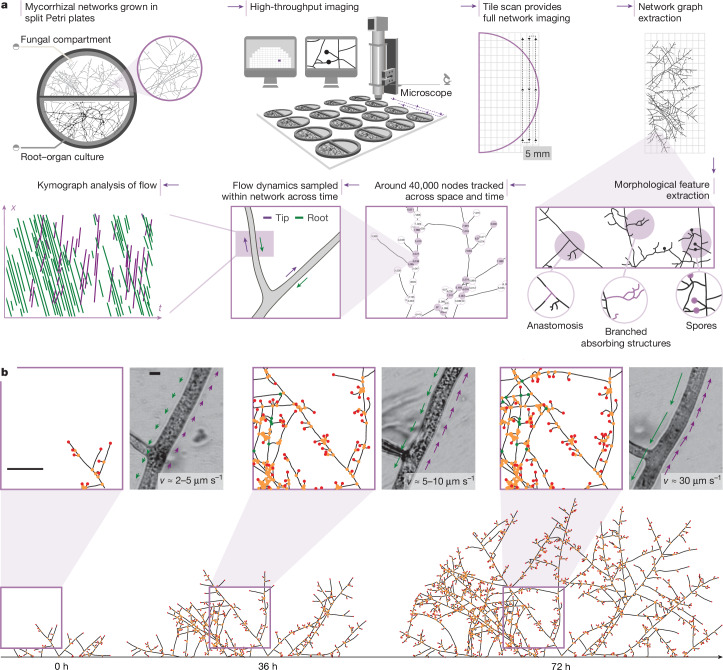
Overview of the data-extraction techniques. **a**, Schematic of the high-throughput imaging and analysis pipeline for extracting network architecture and flow dynamics across scales. **b**, Network skeleton and nodes (tip nodes (red), branch nodes (orange) and anastomosis nodes (green)) extracted are shown at three timepoints spanning 72 h. Magnified typical image frames; the observed maximum speeds from example videos of bidirectional cytoplasmic flows are shown. The directionality and speed of flows are illustrated by arrows. The larger arrows represent faster speeds; the green arrows point towards the root; and the purple arrows point away from the root. Scale bars, 10 mm (left) and 10 μm (right).

To date, difficulties in simultaneously tracking dynamic topologies of mycorrhizal networks, while measuring their internal cytoplasmic flows, have precluded understanding how symbiotic fungi modulate their anatomical architecture and transport patterns to meet trade demands. To overcome these challenges, we built an imaging robot enabling time-resolved microscopy of network topologies in up to 40 in vitro root organ culture (ROC) plant–fungal replicates simultaneously (Fig. 1a and Methods). A typical experiment acquired 150 images per replicate every 2 h at ×2 magnification, with an image overlap of about 20%. This configuration enables imaging of full network graphs by constraining growth to two dimensions, but basic symmetry considerations suggest relevance for fungal growth in three-dimensional soils (Supplementary Discussion). Through computational image analysis (Supplementary Methods), we extracted the full network graph at every timepoint (Supplementary Video 1) and tracked every node (growing tips, hyphal branches and junctions) and every edge (hyphal segments between nodes) across time, assigning each element a unique identifier (Supplementary Video 2). A typical experiment tracked around 40,000 nodes per plate and about 500,000 nodes across replicates. Using element-by-element tracking, we created time-lapse videos of fungal trade routes and monitored architectural rearrangements across the symbiotic network, such as hyphal fusion (that is, anastomosis) and timing/location of spore formation (Fig. 1b).

To image cytoplasmic flows within hyphal networks, we switched to ×100 magnification at targeted coordinates within the mapped networks for high-resolution video analyses (Supplementary Video 3). We quantified flow behaviour and velocity statistics, zooming into nodes and edges of interest. From these sequences, we constructed kymographs and extracted the speeds of bidirectional flows (Fig. 1a and Methods) to examine whether and how flow dynamics are related to topological network features (Fig. 1b). This enabled us to link around 100,000 individual flow trajectories to precise coordinates within the growing network.

We tracked mycorrhizal networks generated by three fungal strains: *Rhizophagus irregularis* A5 (DAOM664344), *R. irregularis* C2 (DAOM664346) and *Rhizophagus aggregatum*. We grew networks in two-compartment Petri plates. The colonized host, in vitro ROC *Daucus carota*, was restricted to the root compartment^16^. The fungal network crossed a physical barrier to a second compartment with additional phosphorus, inaccessible to the plant partner, lined with permeable cellophane to optimize visualization (Methods). From extracted network graphs, we computed spatial density profiles of hyphae and growing tips, growth speeds at growing tips and identified extraradical structures, including runner hyphae (RH), anastomoses, branched absorbing structures (BASs) and reproductive spores (Fig. 1a).

## A travelling wave with self-regulation

**Fig. 2:**
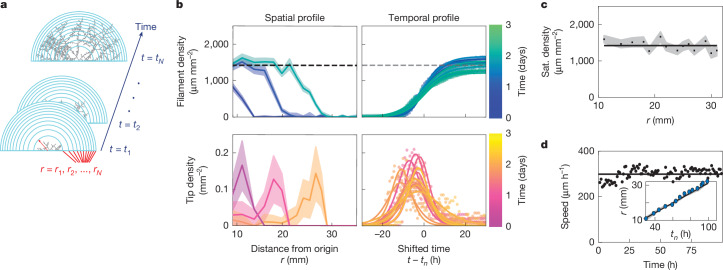
Hyphal-filament and growing-tip densities over time. **a**, Schematic showing concentric rings of equal area centred at the base of the colony for density estimates. The densities of growing tips and hyphal filaments are spatially averaged at each timestep within every ring and assigned a position corresponding to the radius *r* = *r*_1_, …, *r*_*N*_ of the ring and the arrival time of the network at each ring *t*_1_, ..., *t*_*N*_. **b**, Left, the spatial distribution of filament density (blue–green) and tip density (pink–orange) over 3 days. The shaded region corresponds to uncertainty in density estimates computed by bootstrapping. Colour gradients (blue to green and purple to orange) indicate time. Right, temporal filament and tip density dynamics within each ring, coloured by the arrival time *t*_*n*_ of network at each ring. The circles are individual datapoints and the solid lines are fits of sigmoid and sigmoid derivative functions. Time is shifted for each plot by arrival time *t*_*n*_ ∈ {*t*_1_ … *t*_*N*_}. **c**, Saturating (sat.) filament density as a function of ring radius. The solid line is a linear fit, and the shaded region is the confidence interval obtained by bootstrapping sigmoid fits of each density curve. **d**, The speed of growing tips over time. The black points are the average growth speed of hyphae at the front of the colony at each timestep. The black line is the average of the black points. Inset: the position of the wavefront over time, obtained from a sigmoid fit to the filament density. The black line is a linear fit. The shaded region is the bootstrapped confidence interval as in **c**.

**Extended Data Fig. 1:**
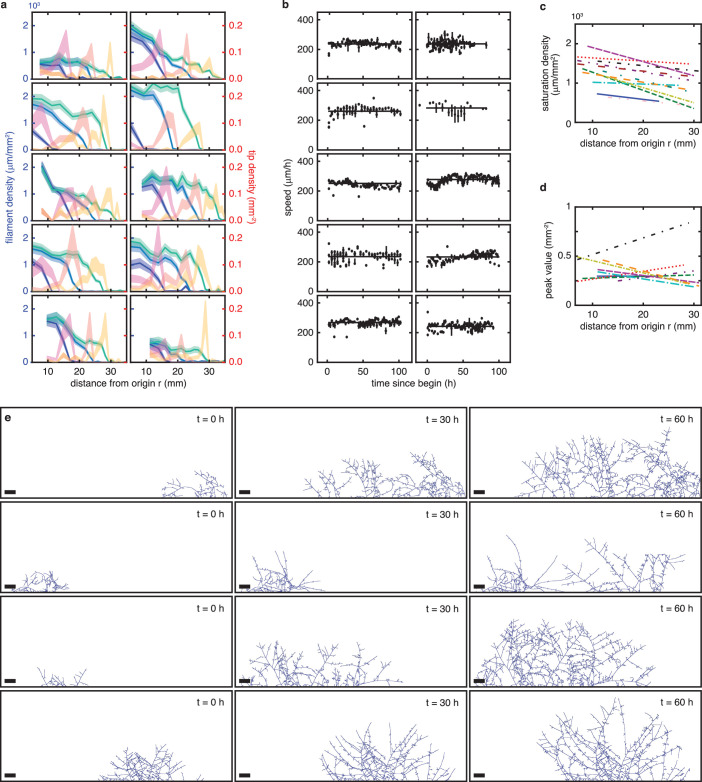
Replicates of *R. irregularis* A5 plates show consistent travelling wave pattern. (**a**) Spatial distribution of filament density (blue) and tip (red) density over three consecutive days (colour gradient, with dark colours going to lighter from day one to three). Shaded region corresponds to uncertainty in density estimates computed by extrapolating the ones obtained for the plate in the main text (Methods). Profile shown at regular intervals separated by 30 h, while distance from origin is computed under the assumption of half circular colonization of the plate (**b**) Speed of growing tips at the front over time. Black points are the average hyphal growth speed at the front of the colony at each timestep. Error bars represent mean ± s.e.m. Black line is the average of black points. (**c**) Saturation density as a function of ring radius. Each line corresponds to the linear fit across saturation value obtained by fitting a sigmoid to density curves for each replicate. (**d**) Growing tip density peak value as a function of ring radius. Each line corresponds to the linear fit across peak values obtained by fitting the derivative of a sigmoid to growing tip density curves for each replicate. (**e**) Additional examples of extracted networks. Each row represents different timepoints of the same replicate. Scale bar is 1 mm.

First, we examined whether mycorrhizal fungi formed trade networks in a consistent, repeatable manner over time. We determined the network architecture of *R. irregularis* A5, tracking the full network graph over time. The exact topology varied among replicates (Extended Data Fig. 1), but when we computed radially averaged spatial densities of growing tips and hyphal filaments (Fig. 2a and Methods), we identified a simple and reproducible pattern. When plotted against the distance *r* from the barrier crossing point (Fig. 2b), the hyphal filament density profile was plateau shaped, with a flat region toward the centre *r* → 0 and a sloping decay to zero toward the periphery *r* → ∞ (Fig. 2b (top)). By contrast, the density profile of growing tips had a peaked shape and was positioned within the decay zone of hyphal density (Fig. 2b (bottom)). Notably, the density of both hyphal filaments and growing tips had spatial profiles that were nearly invariant across time (Fig. 2b (left)) and temporal profiles that were invariant across space (Fig. 2b (right))—translational symmetries indicative of a wave advancing in space.

Together, these data suggest that AM fungi explore space following a morphogenetic pattern that is best described as a travelling wave^17–19^—a phenomenon observed in bacteria and other microorganisms^20,21^, but, to our knowledge, never before documented in symbiotic fungi. AM fungi grew as a singular wave of space-filling mycelium, made up of two intimately coupled populations: (1) growing tips that lead the wave as a pulse in space; and (2) hyphal filaments that densify the space in the wake of the advancing pulse (Supplementary Video 4). Consistent with this description, hyphal filament densities behind the wavefront saturated at a constant value across space (Fig. 2c), and wave speed was also independent of time (Fig. 2d).

To understand how the observed travelling-wave pattern emerged from the underlying microscopic processes, we developed a simple model of mycorrhizal network growth (derivation and a detailed discussion is provided in the Supplementary Discussion). This model, inspired by previous studies on microbial colony growth^20,22–24^ and branching morphogenesis^25,26^, describes the coupled dynamics of growing tip density *n* (number per unit area (mm^−2^)) and hyphal filament density *ρ* (length per unit area (μm mm^−2^)):

1 $${\rm{\partial }}n/{\rm{\partial }}t=b(n)-a(n,\rho )+{\rm{\nabla }}\cdot {\boldsymbol{J}}(n)$$

2 $$\partial \rho /\partial t=vn$$

The dynamics of tips (equation (1)) are governed by the rates of tip birth due to branching *b*(*n*), tip annihilation due to anastomosis *a*(*n*,*ρ*), and tip spatial flux ***J***(*n*) due to tip movements driven by apical growth and subapical branching. The dynamics of hyphal filaments (equation (2)) are determined by the local density *n* and speed *v* of growing tips.

**Fig. 3:**
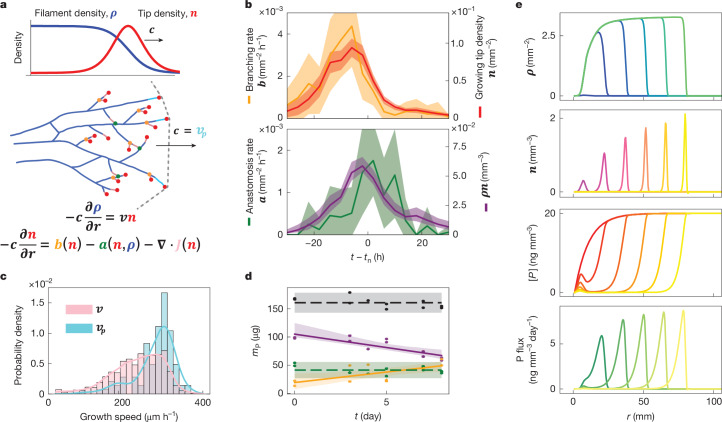
Minimal model of self-regulating travelling wave. **a**, Top, typical density profiles in the wave. Bottom, BARE wave model defined by two coupled partial differential equations for *n* (red, growing-tip density) and *ρ* (blue, hyphal-filament density), describing the dynamics of branching at rate *b* (orange), anastomoses at rate *a* (green) and tip movement by spatial flux ***J*** (pink), and yields wave speed *c*. Middle, wave propagation driven by fast tip growth of puller hyphae at the front (red dots, cyan tails) and densification through slower growth of tips behind the front (red dots and pink tails). Tips duplicate by branching (orange dots) close to growing tips and annihilate by anastomosis (green) with existing hyphae. **b**, The dynamics of the model variables in the ring reference frame. The lines show the average over all of the rings for branching rate *b* (orange), growing tip density *n* (red), anastomosis rate *a* (green) and the product of filament and tip densities *ρn* (purple). The shaded areas show the mean ± 2 s.e.m. **c**, The hyphal growth speed distribution across 100 h in a single replicate. Pink, speeds *v* of all hyphae. Cyan, speeds *v*_*p*_ of puller hyphae with tips at the wavefront. The lines show kernel density estimates of histograms. **d**, Phosphorus (P) absorbed by the expanding colony is transferred to the host root. P in the fungal-only compartment (purple) decreased, P in the root (orange) increased and P in the root compartment agar (green) remained constant. The total P across all compartments (black) remained constant. The points correspond to individual replicates, shown together with linear fit ± bootstrapped 95% confidence intervals of fit (solid lines and shading) and mean ± 2 s.e.m. (dashed lines and shading). **e**, Travelling-wave growth drives a P-depletion front. Numerical integration results for the BARE wave model of equations (1) and (2), together with Supplementary equation (6) to account for diffusion-limited P absorption. Spatial profiles (from top to bottom) for *ρ*, *n*, P concentration [*P*] and P flux are shown. The colour gradient indicates time (*t* = 0 h to *t* = 600 h).

This pair of coupled equations (equations (1) and (2)), which we call the branching and annihilating range expansion (BARE) wave model, has solutions that capture the observed travelling-wave dynamics (Fig. 2), where the wave speed *c* and saturating density *ρ*_sat_ are both constant (Fig. 3a, Supplementary Discussion and Extended Data Figs. 11 and 12). A sufficient condition for the existence of such solutions is that branching and anastomosis rates are well-approximated as *b*(*n*) = *αn* and *a*(*n*,*ρ*) = *βnρ*, respectively^18^, where *α* and *β* are constants. We confirmed that our data are compatible with these relationships, finding that temporal profiles of *b*(*n*) and *a*(*n*,*ρ*) had shapes that closely matched those of *n* and *nρ*, respectively (Fig. 3b), up to linear scaling factors *α* (about 0.04 h^−1^) and *β* (about 23 μm h^−1^), respectively. Thus, tips multiply at a rate *α* *≈* 4% per hour, and anastomose at a rate close to *β* × *ρ*_sat_ ≈ 2% per hour.

The model further predicts that the wave speed *c* is set by the fastest-growing subpopulation of tips at the front, which effectively ‘pull’ the wave^18^ (Supplementary Discussion). Consistent with this prediction, we confirmed that the speed *v*_p_ (about 280 µm h^−1^) of the subpopulation of ‘puller’ tips at the advancing wavefront (Figs. 2d and 3c (cyan)) closely matched the wave speed *c* (about 280 µm h^−1^, Fig. 2d (inset)). By contrast, the average growth speed of the entire tip population $\langle v\rangle$ (about 240 μm h^−1^) obtained by tracking (Fig. 3c) was nearly 15% lower. These data demonstrate that AM fungal travelling waves are ‘pulled waves’ whose speed *c* is determined by puller tips at the wavefront (Fig. 3a (cyan)) and the saturation density *ρ*_sat_ is set by the balance of branching and anastomosis (through *α* and *β*, respectively; Supplementary Discussion).

**Extended Data Fig. 2:**
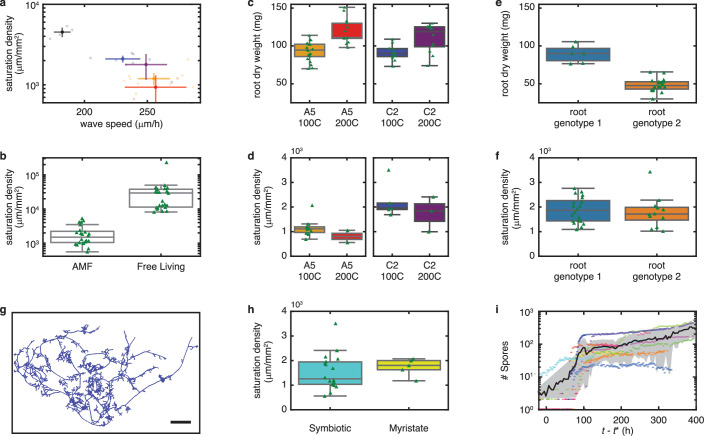
AM fungi consistently regulate network structure despite changes in carbon supply, root genotype, or even absence of a host root with carbon supplied as myristate. (**a**) AM fungal networks demonstrate a speed-density trade off, with saturation density and wave speed depending on fungal strain. On the same host root genotype, different fungal strains grow differently, with some strains growing sparse but fast networks, while others grew slower, denser networks. Scatter plot of saturation density and wave speed for all replicates. Data shown for *R. irregularis* A5, grown under control conditions (blue) or with root grown under double the amount of carbon in root compartment (purple),* R. irregularis* C2 grown under control conditions (orange) or with root grown under double the amount of carbon in root compartment (red), *R. aggregatum* (black). Error bars correspond to mean ± 2 s.e.m. (**b**) Saturation density (m/mm^2^) of AM fungi is substantially lower than free living fungi. Saturation density of AM fungi vs saturation density in data from non-chord-forming, free-living fungi as reported in Aguilar-Trigueros et al.^28^. (**c**) Comparison of root dry weight under control conditions and when carbon concentration for root compartment is doubled in replicates grown on root genotype 1 together with *R. irregularis* A5 (left) and* R. irregularis* C2 (right). (**d**) No difference in saturating hyphal density of* R. irregularis* A5 (left) and *R. irregularis* C2 (right) under control conditions and when carbon concentration for root compartment are doubled. (**e**) Carrot root genotype 1 (blue box) and genotype 2 (orange box) differ in root dry weight after 45 days of growth (**f**) Despite being grown on different root genotypes,* R. irregularis* C2 networks reached the same saturating hyphal density. (**g**) Network image of *R*. *irregularis* C2 grown in myristate in the absence of a host root after ≈14 days of growth. Scale bar: 1 mm. (**h**) *R*. *irregularis* C2 networks reached similar hyphal density after ≈14 days of growth, regardless of whether they are grown with or without a host root when supplied with myristate. Saturating hyphal density of AM fungi vs hyphal density of AM fungi grown in a myristate medium. In (**b-f**,**h**), triangles correspond to individual replicates and the box represents the interquartile range (IQR), with the central line indicating the median. The whiskers extend from the box to the minimum and maximum values within 1.5 times the IQR. (**i**) Total number of spores across the network jumps rapidly at variable times, and then grows more gradually. Data from the same 12 sample plates (dots coloured by replicate) as in Fig. 4b–e. Black line corresponds to the mean across all samples and the grey region represents mean ± s.d., plotted along a time axis offset by ${t}^{* }$, the time at which each sample’s network length reached ${L}_{\text{RH}}=1{0}^{2}$ mm.

These results are surprising because, in typical population waves, such as those observed in bacterial colonies, microorganisms exhibit growth up to a density ceiling imposed by the environmental carrying capacity^20,21,23,24^. By contrast, we found growth of mycorrhizal networks saturating at very low network densities (as low as 1,000 μm mm^−2^ for *R. irregularis* A5; Extended Data Figs. 1 and 2a). We also found that AM fungal densities observed here were an order of magnitude lower than those found for free-living (that is, non-trading) fungi (Extended Data Fig. 2b), which tend to continue exponential growth over a similar range of increase in total network length (10^1^−10^3^ mm)^27,28^. This difference in growth pattern between AM fungi and free-living fungi raises the interesting question of whether density saturation in these symbiotic fungi is driven by environmental carrying capacity alone, or whether it represents a specific growth strategy.

To address this question, we compared the saturation densities of AM fungi under changed environmental conditions, specifically modulating carbon availability to the fungus (Supplementary Methods). Despite changing both the root biomass (larger versus smaller) and root genotype (fast versus slow growing), we found no substantial difference in the density ceiling of the mycorrhizal network (Methods and Extended Data Fig. 2c–f), suggesting that the growth strategy was under fungal control. To further examine how fungi controlled their own growth when new hyphae enter into the network, we analysed how the collisions between fungal waves impacts density in the merged wave. We found no density increase, suggesting that fungi strictly regulate their saturation density through anastomosis (Supplementary Video 7 and Extended Data Fig. 9). We further confirmed that saturation density was under fungal control by growing AM fungi in the absence of a host root, replacing the in vitro root with 0.5 mM myristic acid (Methods), which allows AM fungi to grow and reproduce asymbiotically^29^. We found that AM saturation density was again invariant, with or without a host root (Extended Data Fig. 2g,h).

**Extended Data Fig. 3:**
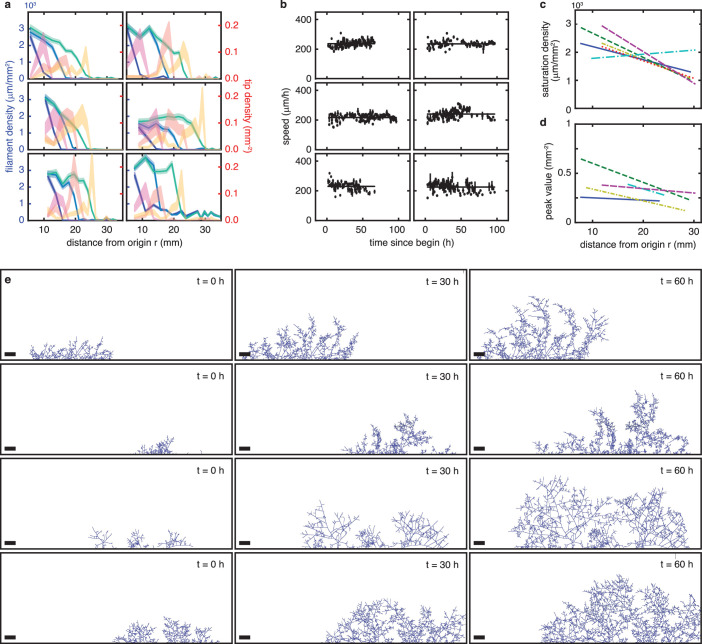
Replicates of *R. irregularis* C2 plates show consistent travelling wave pattern. (**a**) Spatial distribution of filament density (blue) and tip (red) density over three consecutive days (colour gradient, with dark colours going to lighter from day one to three). Shaded region corresponds to uncertainty in density estimates computed by extrapolating the ones obtained for the plate in the main text (see Methods). Profile shown at regular intervals separated by 30 h, while distance from origin is computed under the assumption of half circular colonization of the plate. (**b**) Speed of growing tips at the front over time. Black points are the average hyphal growth speed at the front of the colony at each timestep. Error bars represent mean ± s.e.m. Black line is the average of black points. (**c**) Saturation density as a function of ring radius. Each line corresponds to the linear fit across saturation value obtained by fitting a sigmoid to density curves for each replicate. (**d**) Growing tip density peak value as a function of ring radius. Each line corresponds to the linear fit across peak values obtained by fitting the derivative of a sigmoid to growing tip density curves for each replicate. (**e**) Additional examples of extracted networks. Each row represents different timepoints of the same replicate. Scale bar is 1 mm.

**Extended Data Fig. 4:**
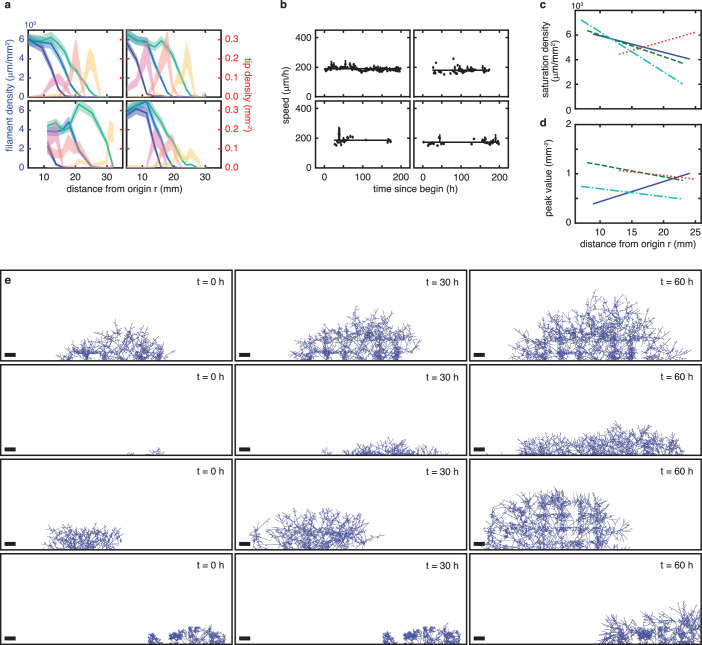
Replicates of *R. aggregatum* plates show consistent travelling wave pattern. (**a**) Spatial distribution of filament density (blue) and tip (red) density over three consecutive days (colour gradient, with dark colours going to lighter from day one to three). Shaded region corresponds to uncertainty in density estimates computed by extrapolating the ones obtained for the plate in the main text (see Methods). Profile shown at regular intervals separated by 30 h, while distance from origin is computed under the assumption of half circular colonization of the plate. (**b**) Speed of growing tips at the front over time. Black points are the average hyphal growth speed at the front of the colony at each timestep. Error bars represent mean ± s.e.m. Black line is the average of black points. (**c**) Saturation density as a function of ring radius. Each line corresponds to the linear fit across saturation value obtained by fitting a sigmoid to density curves for each replicate. (**d**) Active tip density peak value as a function of ring radius. Each line corresponds to the linear fit across peak values obtained by fitting the derivative of a sigmoid to growing tip density curves for each replicate. (**e**) Additional examples of extracted networks. Each row represents different timepoints of the same replicate. Scale bar is 1 mm. R. Aggregatum forms very thin and densely packed hyphae. The segmentation of this strain can be sensitive to illumination. Non-uniform illumination can cause non-uniform detection of these hyphae which explains the checkboard pattern observed in some plates.

If travelling-wave range expansion with a self-regulated density is a general feature of AM fungal growth, we would expect to see analogous patterns across different AM strains and species. We therefore measured networks of a different strain, *R. irregularis* C2, and a different AM species, *R. aggregatum*. Although both wave speed and saturation densities varied, all replicates showed a uniformly translating wavefront followed by a density profile that saturated at a low density, indicating a similar self-regulating growth strategy (Extended Data Figs. 3 and 4). These saturation densities correlated negatively with wave speeds, indicating a trade-off: strains that grew to higher densities demonstrated slower wave speeds, and vice versa (Extended Data Fig. 2a). Taken together, these findings suggest that self-regulating travelling waves represent a general growth strategy for AM fungi that favour spatial exploration over local densification.

## Regulated waves align trade interests

We next examined why AM fungi prioritize spatial exploration in their travelling-wave growth strategy. In contrast to free-living organisms, symbiotic trade requires that mycorrhizal fungi budget their imported carbon to balance nutrient export to host roots with network growth in search of new trade partners^3^. Self-regulating travelling waves could help to balance these conflicting demands because costs of building exploratory tips for new trade opportunities can be compensated by accompanying waves of absorbing mycelium that are just dense enough to extract and transport nutrients back to roots, in exchange for more carbon.

**Extended Data Fig. 5:**
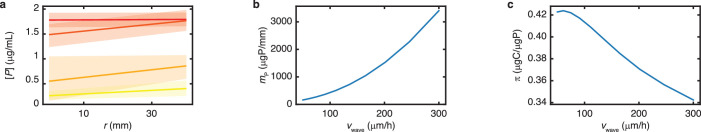
Pattern of spatial phosphorus (P) absorption aligns with regulated fungal wave growth. (**a**) P concentration of agar in fungal compartment of root organ cultures measured at 4 times points starting after fungal network crosses into fungal-only compartment (red = 0 days, dark orange = 3 days, light orange = 6 days, yellow = 9 days). $r=0$ mm corresponds to cross-over point where fungal colony enters fungal-only compartment and $r=40$ mm corresponds to the opposite end, where P depletion will take the longest to form. By 9 days (yellow line), P is almost completely depleted from the agar in the fungal-only compartment. Error bars correspond to mean ± 2 s.e.m. (**b-c**) The data of (**a**) were used to parameterize the model of P depletion by the fungal travelling wave defined in Supplementary Discussion 4.2.5. (**b**) Total phosphorous captured by the colony per unit length of root over 600 h of colony propagation (see Fig. 3e for spatial profiles) as a function of wave speed ${v}_{\text{wave}}$. At a fixed saturation density, the more the fungal colony invests in spatial exploration (i.e. the higher the wave speed), the more phosphorous it can absorb from its environment. This is because it can better escape its self-generated P depletion zone. (**c**) Carbon cost for the phosphorous captured by the colony over the same 600 h interval. The cost is defined as the total carbon cost of growth divided by the total amount of P acquired over the duration of travelling-wave growth. Carbon cost is calculated in the same manner as was done for experimental data, as explained in Supplementary Discussion 4.3.4.

To test this idea, we quantified phosphorus transported back to the host root across the expanding network. As expected, phosphorus in the root increased, whereas phosphorus in the fungal compartment was accordingly reduced (Fig. 3d). By quantifying phosphorus concentration in the growth medium near to (*r* ≈ 0 mm) and far from (*r* ≈ 40 mm) the root compartment, we found that a spatial gradient of phosphorus depletion developed over time, until fungal compartment phosphorus was entirely depleted (Extended Data Fig. 5a). This agrees with transcriptomics showing distinct spatial and temporal gene expression patterns for phosphorus absorption across hyphal networks^30^.

Using these data, we expanded the BARE wave model (Supplementary Discussion) to include phosphorus absorption by the network^15^, showing that a phosphorus gradient forms and propagates together with the advancing wavefront (Fig. 3e). The model suggests that, at a given saturation density, the more that the fungal colony invests in spatial exploration (that is, higher wave speed), the more phosphorus that it can absorb from its environment. This is because the network can better escape its self-generated phosphorus depletion zone, leading to an overall lower carbon cost per unit of acquired phosphorus (Extended Data Fig. 5b,c). As a result, plant and fungal interests are highly aligned because relatively small carbon investments can fuel fungi to expand beyond nutrient depletion zones, fostering longer-range spatial exploration for both new roots and new nutrient patches.

**Extended Data Fig. 6:**
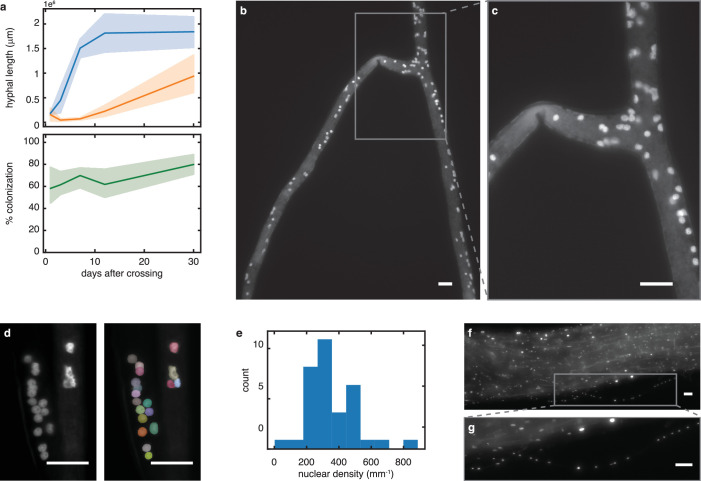
Intraradical growth and colonization increase concurrently with extraradical growth. (**a**) Temporal variation of (top) total extraradical hyphal length in m x1e^6^ (ERM, blue), total intraradical mycorrhizal length (IRM, orange) as measured by ddPCR, and (bottom) per cent colonization (green), as measured using Trouvelot method. AM fungal networks were grown and harvested destructively over 30 days. Lines connect the means at 1, 3, 7, 12 and 30 days after crossing, shaded regions correspond to mean ± 2 s.e.m. at each timepoint. For details of ddPCR and Trouvelot, see Methods. (**b-g**) Methodology for quantifying nuclear density. Nuclear imaging and segmentation for the extraradical hyphae for *R. irregularis* A5, using DAPI (4′,6-diamidino-2-phenylindole) blue-fluorescent DNA dye. Scale bars indicate 20 μm (**b-d**), and 100 μm (**f-g**). Panel (**b**) shows an example of the distribution of DAPI stained nuclei in the extraradical hyphae, while (**c**) is an enlarged cropped image at the extraradical hyphae intersection (see Supplementary Video 8). Panels (**d**) show the segmented nuclear masks – a process to automatically recognize nuclei and calculate their distribution and density. Unique colours are automatically attributed to each nucleus via the trained StarDist model. (**e**) Number of nuclei per millimetre of extraradical hyphal length as measured by DAPI staining. (**f**) Intraradical colonization of the host root, with DAPI staining both the nuclei and the host root nuclei. (**g**) Enlarged cropped image of the hyphae near the root’s surface, showing only the hyphal nuclei.

Given that carbon resources for fungal wave expansion comes from host roots, we would also expect intraradical colonization to increase within roots over time. By sequentially harvesting replicates, we found that the length of intraradical hyphae significantly increased over time, concurrently with extraradical network growth (Extended Data Fig. 6). Although live-tracking of intraradical colonization is currently only possible over small spatial scales using plant-based fluorescent protein reporters, data for *R. irregularis* on rice plants suggest that fungi form ‘arbuscular fronts’ that move down roots at a speed of about 15 μm h^−1^ (ref. ^31^), roughly 5% of the speed that we measured for the extraradical wavefront (see ref. ^32^ for *Glomus mosseae* arbuscular fronts).

## Topology and morphology changes over time

**Fig. 4:**
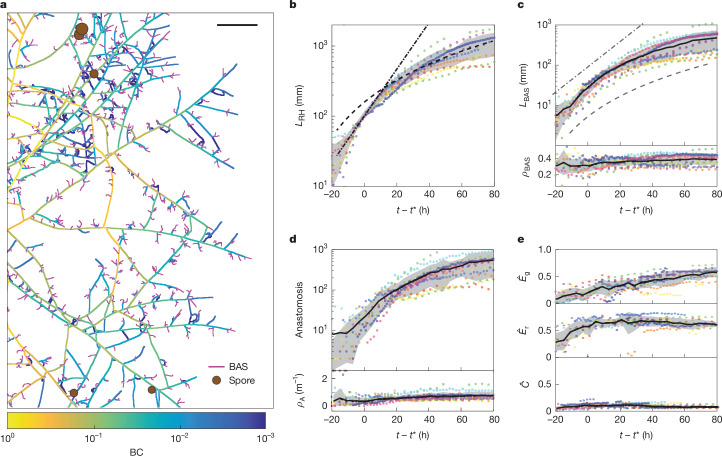
Graph statistics reveal a developing symbiotic supply-chain network. **a**, Network graph plotted together with BAS (magenta) and spores (brown circles). The graph edges are coloured by their betweenness centrality value (BC)—indicating the density of the shortest paths to root compartment—from the lowest (blue) to highest (yellow) BC. The arrows indicate examples of orthoradial edges that formed loops after anastomosis. Scale bar, 5 mm. **b**, The total RH length, *L*_RH_, over time. The black lines show exponential (dot dashed) and quadratic (dashed) fits, at early (<20 h) and later (>40 h) times, respectively. **c**, The total BAS length *L*_BAS_ (top) and the BAS length density *ρ*_BAS_ = *L*_BAS_/(*L*_RH_ + *L*_BAS_) (bottom) versus time, with exponential growth (dash dotted line) and quadratic growth (dashed line) guides for the eye. **d**, Cumulative anastomosis events (top) and anastomosis density *ρ*_A_ (bottom) versus time. **e**, Normalized global (${\hat{E}}_{{\rm{g}}}$) and root (${\hat{E}}_{{\rm{r}}}$) geometric transport efficiencies, and carbon cost ($\hat{C}$) versus time. In **b**–**e**, data are from 12 replicates (dots are coloured by replicate ID), the black line is the mean across all replicates, the grey region represents mean ± s.d., with a time axis offset by t*, the time at which each replicate’s network length reached *L*_RH_ = 10^2^ mm.

We next examined the underlying network architecture that supports these waves of carbon and phosphorus trade activity, and how fungal architecture, including investment in structures to absorb nutrients, changes over time to meet these demands. Previously, it was difficult to follow the dynamics of individual structures across the network to test how AM fungi differentially invest in nutrient absorptive capacity (through BASs^33^), spatial exploration (through growth) and reproduction (through spores). Our automated network extraction enabled network-wide tracking of these structures throughout the course of network growth (Fig. 4 and Extended Data Fig. 2i).

Consistent with self-regulated travelling-wave growth (with constant wave speed *c* and saturation density *ρ*_sat_), we found that the total length *L*_RH_ of all RH edges of the network transitioned from exponential to quadratic growth (*L*_RH_ ∝ *ρ*_sat_(*ct*)^2^) shortly after crossing into the fungal compartment (Fig. 4b). Simultaneously, the fungus constructed nutrient absorbing BAS structures at a uniform rate, such that approximately 30% of the network length was consistently composed of BAS (Fig. 4c). In agreement with past transcriptomics data^30^, this high and constant investment in BAS suggests that these structures have a key role in mediating nutrient trade^33^. Continual investment in BAS behind exploratory tips is required for phosphorus absorption because arbuscules will collapse prematurely if insufficient phosphorus is supplied to the plant by the fungus^34^. Once the BAS were established, we observed that the timing of sporulation—the onset of reproduction in AM fungi – varied among replicates, but gradually increased to an average of around 300 spores per network at 400 h (Extended Data Fig. 2i).

To construct expensive structures such as spores, which are packaged with large quantities of plant-derived carbon^35^, and growing tips, where biosynthetic carbon expenditures are concentrated^35^, fungi must efficiently transport resources across the network. We therefore examined how major transport routes were distributed in space as the network matured. To quantify the relative importance of each hyphal edge for network-scale transport, we calculated its betweenness centrality (BC), which quantifies the relative abundance of shortest paths passing through a network element^36^.

**Extended Data Fig. 7:**
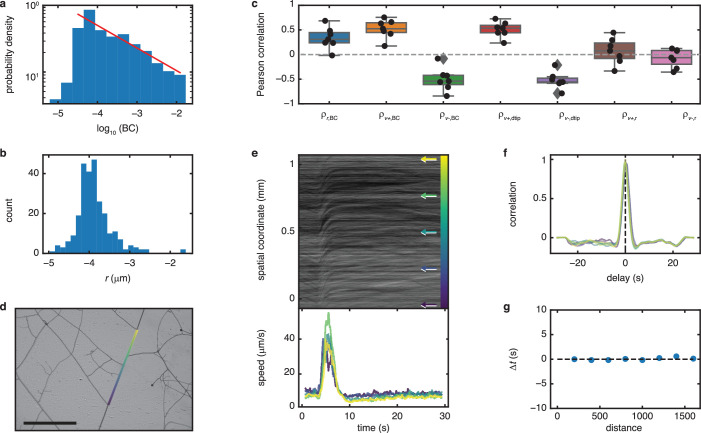
Flow statistics correlate Betweenness Centrality (BC) hierarchy and indicates incompressibility. (**a**) Length-weighted distribution of hyphal BC across the entire network demonstrates a long-tailed power-law distribution $P({BC}) \sim {{BC}}^{-\gamma }$ with $\gamma \approx 0.3$, indicating hierarchical order. (**b**) Distribution of hyphal radius extracted from high-resolution videos. (**c**) Correlation between measured edge observables: radius $r$, betweenness centrality BC, distance to tip ${d}_{\text{tip}}$, the velocity of particles moving towards the tip ${v}_{+}$, and towards the root ${v}_{-}$. Velocity sign is positive when particles move towards the tip and negative when they move towards the root. Each dot represents the Pearson correlation coefficient computed over all edges belonging to a replicate plate sample. Box represents interquartile range (IQR), with central line indicating median. Whiskers extend from box to minimum and maximum values within 1.5 times IQR. (**d-g**) Flow speeds are highly correlated across space and time with no detectable lag, consistent with incompressible flow. (**d**) Close-up of a long hypha imaged at 4x magnification. Colours indicate spatial coordinate along a single hyphal segment across which kymograph analysis was conducted. Scale bar: 1 mm. (**e**) Kymograph (top) and speed time series (bottom) of flows within the coloured hyphal segment in (**d**), for a 30 s interval in which rapid changes in flow speed were observed. Arrows indicate spatial position for speed time series of matching colour. (**f**) Cross-correlation of speed time series at *x* = 1.2 mm in (**e**) against all other speed time series in (**e**). All curves were nearly identical, peaking at a high cross-correlation value centred at a delay close to zero. (**g**) Cross-correlation delay $\Delta t$ corresponding to the peak position of each curve in (**f**), as a function of the distance along the hypha, was very close to zero and demonstrated no dependence on distance, consistent with incompressible flow.

We calculated and coloured hyphal edges by their BC value to represent the cumulative number of shortest paths passing through them that connect nodes in the network to the root compartment (Fig. 4a and Supplementary Methods). We found that BC tended to increase along each RH in the direction toward the root—a pattern expected for tree-like networks whereby each successive branchpoint integrates root-ward shortest paths from terminal nodes (that is, hyphal tips). The resulting distribution of BC across all network edges exhibited a long (power-law) tail (Extended Data Fig. 7a), characteristic of hierarchical planar networks and observed across a variety of infrastructural systems^37^ (Supplementary Discussion). However, we also found multiple instances of hyphae with a non-monotonic pattern of BC, reflecting the presence of numerous loops created by anasotomsis events, which occurred at a nearly constant rate throughout network growth (Fig. 4d). These fusion events are particularly interesting for the symbiotic context because they alter network topology by forming new connections between RH radiating outwards from the root. The resulting ‘orthoradial’ hyphae (Fig. 4a (arrows)) can lead to transport ‘short cuts’ toward potential new hosts. We therefore examined whether and how these topological features of the network graph changed over time, incorporating data on hyphal fusion.

To quantify the benefits of the network’s spatial layout, we computed the graph theoretical measure of geometric efficiency, *E*, which compares the distance *d*^sp^ along the shortest path between pairs of nodes to the shortest possible distance (that is, the Euclidean distance) *d*^E^ in the physical space embedding the network^4,38–41^. As AM fungal networks are symbiotic, we computed *E* in two contrasting contexts: (1) global efficiency (*E*_g_), for transport between arbitrary pairs of nodes within the network; and (2) root efficiency (*E*_r_), for transport between network nodes and the host root (Supplementary Methods). Thus, increasing *E*_r_ benefits trade with an already established partner, whereas increasing *E*_g_ enhances readiness for potential new trade partners encountered during growth.

However, from a network design perspective, simply maximizing *E*_g_ or *E*_r_ is unlikely to be the best strategy because it fails to account for associated costs. We therefore estimated the carbon cost *C* for building the network by scaling the measured growth in total network length (Supplementary Discussion), and further normalized *E*_g_, *E*_r_ and *C* using two ‘ideal networks’ that represent extremes in the inherent trade-off between resource costs and efficiency^4,38–40^.

The first, minimum spanning tree (MST), is the shortest possible network that connects all nodes. This is the limit of low *C*, but is geometrically less efficient, that is, requires longer travel between node pairs^40^. The second, Delaunay triangulation (DT) yields a space-filling mesh network that fully connects all nodes—a maximal planar graph^40^. It represents an upper limit for *E* because the shortest paths through the meshed network tend to be close to the Euclidian distance. DT is also robust to damage because the meshed network contains many loops^39^, but is expensive in terms of building costs.

We assessed the costs and benefits of the measured fungal graph relative to these limiting networks, by normalizing *E*_g_, *E*_r_ and *C* to those of MST and DT^42^ to obtain the relative efficiencies^42^ ${\hat{E}}_{{\rm{g}}}$ and ${\hat{E}}_{{\rm{r}}}$ and relative cost $\widehat{C}$ (Supplementary Methods). With this normalization, values approaching unity (closer to DT) suggest network designs maximizing transport efficiency and robustness to damage, whereas values approaching zero (closer to MST) suggest designs that minimize material cost.

We found that the relative root efficiency ${\hat{E}}_{{\rm{r}}}$ (Fig. 4e (top)) remained stable at around 0.5, approximately halfway between MST and DT. Likewise, the normalized carbon cost $\hat{C}$ for building the network (Fig. 4e (bottom)) also remained constant albeit at a level near zero, much closer to MST than DT. By contrast, the relative global efficiency ${\hat{E}}_{{\rm{g}}}$ (Fig. 4e (middle)) gradually increased over time from around 0.2 shortly after crossing to approximately 0.5 at later times. These results indicate that, similar to human-built transport networks^43,44^ and previously studied biological networks^45^, mycorrhizal networks strike a balance between maximizing transport efficiency (DT) and minimizing material cost (MST). However, evidently, AM fungi navigate this trade-off in a time-dependent manner during network development, maintaining ${\hat{E}}_{{\rm{r}}}$ nearly constant while gradually increasing ${\hat{E}}_{{\rm{g}}}$. Together, these data suggest that, as symbiotic networks start to age, their relative efficiencies shift in favour of exploration for new trade partners over exploitation of exchanges with already established partners.

For efficient transport, central edges (that is, those with high BC) must accommodate a larger fraction of the flux than less central edges (with lower BC). Analogous to flows of traffic along road networks^46,47^, increased flux can be achieved in principle by (1) increasing the cross-sectional dimensions of transport routes; (2) the density of the flowing material; and/or (3) the speeds of flows along those routes. We found that the radii of hyphal network edges (which determine cross-sectional dimension) were distributed broadly (Extended Data Fig. 7b) and significantly correlated with BC (Extended Data Fig. 7c). This suggests that AM fungi modulate the width of hyphal edges in a manner informed by network architecture, with edges of higher BC having wider cross-sections to support increased flux.

## Modulation of bidirectional flows

**Fig. 5:**
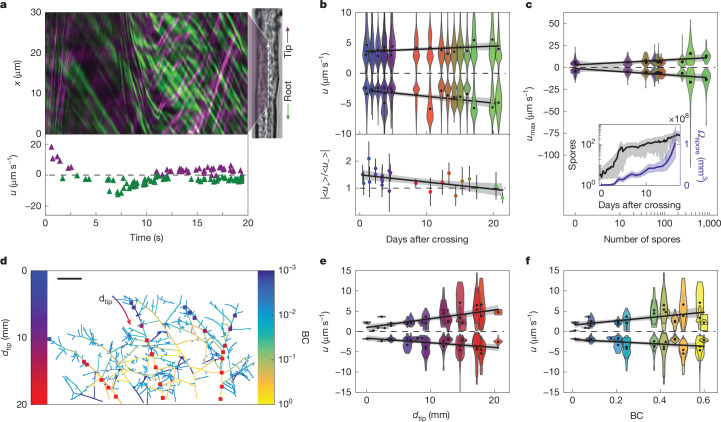
Network architecture connects statistics of bidirectional flows to symbiotic transport requirements. **a**, Top, extracted kymograph example for flow trajectories, with lines corresponding to moving particles coloured in purple (tip direction) and green (root direction). Bottom, extracted particle velocities *u* across time (*u* > 0 toward tip, purple; *u* < 0 toward root, green). **b**, Top, the distribution of observed velocities *u* over the course of network development. Each violin plot distribution includes all velocities extracted from kymographs for each replicate. The violin plot colours follow the time gradient*.* Bottom, the absolute ratio $|\langle {u}_{+}\rangle /\langle {u}_{-}\rangle |$ (mean ± 2 s.e.m.) indicating tipward bias at early times. **c**, The distribution of observed maximum speeds as a function of the total spore number. The violin plot colours are as in **b**. Inset: the total spore number and volume as a function of time. **d**, Example sampling of speed across a network (with edges coloured by BC). The squares correspond to sampled positions, and are coloured by the distance to the tip, *d*_tip_. Scale bar, 5 mm. **e**,**f**, The distribution of observed velocities as a function of *d*_tip_ (**e**) and BC (**f**) for the positions sampled in **d**. The violin plot shows the distribution of velocities from one video at a given position. The violin plot colours are as in **d**. The black points correspond to positive and negative averages for each video. For **b**,**c**,**e**,**f**, the black points indicate the average of the positive ($\langle {u}_{+}\rangle$) and negative ($\langle {u}_{-}\rangle$) parts of the shown velocity distributions; the black lines are linear fits through the black points; and the grey shading represents the bootstrapped 95% confidence intervals of the linear fit.

We next examined how cytoplasmic flows within mycorrhizal networks were organized across space and time to accommodate resource trade. AM fungi are unusual: their networks are composed of one continuous cytoplasm. In these open tubes, AM fungi must move resources both towards and away from the host root^48^. Yet, it is unclear how flows are modulated across networks. To examine whether speed statistics differ in hyphae closer to roots, and whether they change with the age or network position as the wavefront advances, we systematically collected high-resolution, real-time videos of flows at known coordinates across the network. To avoid potential confounding influence of dyes, we conducted label-free imaging using bright-field microscopy (Fig. 5a, (right)). We recorded flow videos at 20–25 frames per second for 20–60 s (Supplementary Videos 3, 5 and 6). In each video, one or more regions of interest (ROIs) were manually defined for kymograph analysis of speeds (Fig. 1a and Methods).

To extract the statistics of speeds in both directions, we used a machine-learning-based kymograph analysis of particle trajectories^49^ (Methods). The resulting kymographs revealed a very rich set of flow behaviours, including simultaneous antiparallel streams of particles within each hyphae, speeds varying significantly across time and space, and abrupt direction switching (that is, sign changes) of streams (Fig. 5a (left) and Supplementary Video 6). Flow behaviours were similarly rich in host-free AM fungal networks grown on myristate as a carbon source (Methods and Supplementary Video 9).

By quantifying over 100,000 particle trajectories, we found that the average speeds in both the tip direction $\langle {u}_{+}\rangle$ and the root direction $\langle {u}_{-}\rangle$ were highly stable over 20 days (Fig. 5b (top)), despite increases in total network length by almost two orders of magnitude (Fig. 4b). However, we did identify a significant directional bias at early times of colony development: tipward average speeds were around 30% faster than rootward average speeds. Notably, this bias gradually decayed to below significance at around 10 days (Fig. 5b (bottom)). We were interested in whether this subsiding directional bias reflects a change in the transport demands as networks develop. At early times, the tipward bias may support growth demands of advancing fronts. At later times, when resources are rapidly acquired by BAS, translocation to roots becomes increasingly important, potentially reflecting changes in trade requirements.

We also observed long tails in the speed distribution from each colony, with speeds often increasing transiently up to 20 μm s^−1^. On rare occasions, we observed short-lived bursts of extremely high speeds, with particles moving between 50 and 120 μm s^−1^. We therefore examined whether high flow speeds were correlated with the emergence of specific network features. By analysing statistics of fastest speeds observed across all ROIs, we found a positive correlation between maximum flow speed and total number of spores (Fig. 5c). As both the spore number (Extended Data Fig. 2i) and spore volume (Fig. 5c (inset)) exhibited increases with time, maximum speeds could reflect either increases in spore number or spore size.

Having observed changes in flow speeds across time, we next investigated whether AM fungi also modulate speeds across space. Driving active flows comes at an energetic cost that increases with speed^50,51^. To reduce costs, fungi might regulate flow speeds across space in ways that meet the demands of different network locations. Specifically, near growing tips, diffusion can be sufficiently efficient for transport within a length scale *L* ≈ 3 mm from the tip (given by *L* = 2*D*/*v*_*g*_, with *v*_*g*_ ≈ 300 μm h^−1^ the measured tip growth speed and *D* ≈ 125 μm^2^ s^−1^ typical of cytoplasmic diffusivity^52^). We therefore expected high-speed, active transport to be absent at around 3 mm from growing tips, where diffusion processes should dominate.

By systematically sampling speeds at different distances *d*_tip_ from growing tips (Fig. 5d), we found that both tipward and rootward speeds decayed towards zero near tips (Fig. 5e). However, the spatial range of decay was unexpectedly long-ranged, with speeds |*u*| depending linearly on *d*_tip_ over the entire sampled range (0 < *d*_tip_ ⪅ 20 mm; Fig. 5e). Although lower speeds near tips (*d*_tip_ ⪅ 3 mm) could reflect dominance of diffusion, we wondered why the flow speeds should continue to vary across much longer ranges across the network. To answer this question, we considered overall network topology. As noted above, increased flux through network edges of high centrality (that is, those with high BC) enhances transport efficiency. This can be achieved by increasing fluid density or speed of flows. Analysing flow velocity dynamics at different points in space revealed statistics consistent with incompressible flow (Extended Data Fig. 7e–g), therefore ruling out variation in fluid density across the network. Thus, we examined the relationship between flow speeds in each edge of the network and the edge’s BC value. We found a similarly simple dependence—speeds |*u*| in both directions were close to zero at low BC and increased systematically on average with increasing BC (Fig. 5f). This trend of increasing |*u*| as a function of both *d*_tip_ and BC was robust across all of the tested samples (Extended Data Fig. 7c). These observations raise the compelling possibility that AM fungi modulate not only hyphal width, but also flow speeds, in a manner that is informed by network topology. This can act to enhance flux through trunk hyphae—transport routes with a higher density of shortest paths to the root. This network-scale flow modulation potentially enables the colony to respond efficiently to demands for lipids at the growing tips and nutrients at the root interface.

## Discussion

We built an imaging robot that enables systematic mapping of network topologies and internal flows across symbiotic mycorrhizal networks. We identified that AM fungi build networks as self-regulating travelling waves. We use the term self-regulating because the wave pattern contrasts with typical microbial colony growth in which waves result from environmental carrying capacities^20^. Instead, AM fungal waves appear to be regulated by density-dependent hyphal fusion events at very low densities (about 1,000 μm mm^−2^), a pattern not found in free-living fungi that densify exponentially to much greater densities^27,28^.

**Extended Data Fig. 8:**
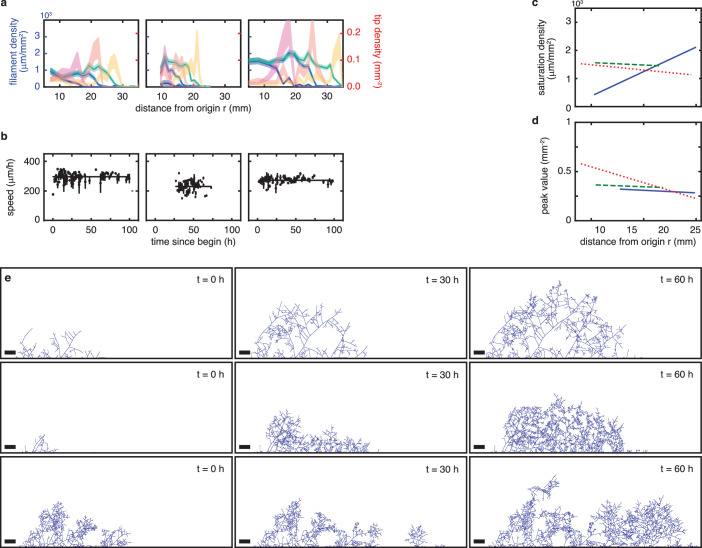
Travelling wave dynamics do not differ between the sucrose and no sucrose treatments in the fungal compartment. (**a**) Spatial distribution of filament density (blue) and tip (red) density over three consecutive days (colour gradient, with dark colours going to lighter from day one to three). Shaded region corresponds to uncertainty in density estimates computed by extrapolating the ones obtained for the plate in the main text (see Methods). Profile shown at regular intervals separated by 30 h, while distance from origin is computed under the assumption of half circular colonization of the plate (**b**) Speed of growing tips at the front over time. Black points are the average hyphal growth speed at the front of the colony at each timestep. Error bars represent mean ± s.e.m. Black line is the average of black points. (**c**) Saturation density as a function of ring radius. Each line corresponds to the linear fit across saturation value obtained by fitting a sigmoid to density curves for each replicate. (**d**) Growing tip density peak value as a function of ring radius. Each line corresponds to the linear fit across peak values obtained by fitting the derivative of a sigmoid to growing tip density curves for each replicate. (**e**) Additional examples of extracted networks. Each row represents different timepoints of the same replicate. Scale bar is 1 mm.

‘Self-regulating’ implies that the program is under fungal control. Although, as obligate biotrophs, the amount of carbon received is ultimately controlled by their plant hosts, our study suggests mycorrhizal fungi precisely control how that carbon is used. We found self-regulating travelling waves across all tested AM fungi (Extended Data Figs. 1, 3, 4 and 8), despite differences in wavefront speeds and saturation densities (Extended Data Fig. 2a). This consistency may reflect an alignment of plant and fungal interests: host carbon to support exploratory tips favouring growth and new trade partnerships must be compensated with nutrients extracted by absorbing mycelium (Fig. 3d,e).

Recent work on free-living fungi^53^ and slime moulds^54,55^ revealed how microorganisms with networked anatomies can response to environmental change by adapting their topology and morphology. Our dynamic mapping of AM fungi network graphs shows how symbiotic morphogenetic programs change across time. We found that BAS density and anastomoses remain nearly constant. This ensures constant (graph-theoretical) transport efficiency towards the host root, while gradually increasing the transport efficiency between arbitrary points in the network. Adding such loops shortens paths to potential new root systems^39^. We also found jumps in spore production at later times, reflecting balanced investment between reproduction and growth as the network matures.

Our systematic mapping of internal flows motivates further questions on how supply-chain dynamics for symbiotic trade are modulated in mycorrhizal fungi during network development. We found flows were consistently and simultaneously bidirectional in most of our 1,600 videos. Notably, flows were faster, and hyphae were wider, along edges with higher BC within the network. This is analogous to hierarchical road networks in which a larger number of lanes and higher speed limits enhance flux along roads of high centrality^47^. This is evidence that intrahyphal flows are controlled by AM fungi in a manner informed by spatial and temporal context.

The architecture of fungal trade networks has been shaped by natural selection for over 450 million years^6^. To understand the flexibility and responsiveness of network design, more data are now needed on how AM fungi modulate travelling-wave strategies after changes in trade partners and resource availability. Mechanistically, what enables antiparallel fluid flows within individual hyphae, and how do AM fungi control nutrient flows despite such a distributed anatomy? Whether these designs can inform human-built supply chain architectures is a compelling question^45^.

## Methods

### Biological material and plate preparation

We performed experiments with Ri T-DNA transformed carrot root (*D. carota* clone DCI) organ cultures colonized by *R. irregularis* strain A5 (DAOM664344), *R. irregularis* strain C2 (DAOM664346)^56^ (I. Sanders) and *R. aggregatum*. We cultivated fungal stocks on modified Strullu–Romand (MSR) medium^57,58^ in association with transformed carrot root for 2–6 months until plates were fully colonized. We then used these stock cultures to inoculate sterile roots, as described below.

Each biological sample contained a root-organ culture in a split Petri plate (94 mm diameter, Greiner Bio-One). One side contained the root colonized with AM fungi, and the other side contained a fungus-only compartment. We designed trapezoid shaped acrylic frames to fit against the central barrier of the two-compartment split plates. These 1-mm-thick frames had a longer top edge (88 mm) than bottom edge (85.5 cm), and a consistent height (12 mm). The frames included a central opening (50 × 2 mm) that was located 2 mm from the top edge. This opening connected to the upper edge of the central barrier of the plate and was covered by a nylon mesh, leaving only a 50-mm-wide window for the fungus (but not the root) to cross through into the second compartment. A nylon mesh (pore size, 50 μm, 9 × 71 mm) was attached to the acrylic frame using UV resin such that the frame opening was fully covered by the mesh and free of resin. The resin was cured with UV light for 3 min. The frames were then wrapped in aluminium foil and sterilized at 80 °C for 72 h.

We filled the two compartments with MSR medium. In the fungus-only compartment, we used regular MSR (per 1 l, 739 mg MgSO_4_·7H_2_O, 76 mg KNO_3_, 65 mg KCl, 4.1 mg KH_2_PO_4_, 359 mg Ca(NO_3_)_2_·4H_2_O, 0.9 mg calcium pantothenate, 1 mg biotin, 1 mg nicotinic acid, 0.9 mg pyridoxine, 0.4 mg cyanocobalamin, 3 mg glycine, 50 mg myo-inositol, 1.6 mg NaFeEDTA, 2.45 mg MnSO_4_·4H_2_O, 0.28 mg ZnSO_4_·7H_2_O, 1.85 mg H_3_BO_3_, 0.22 mg CuSo4_4_·5H_2_O, 2.4 mg Na_2_MoO_4_·2H_2_O, 34 mg (NH_4_)Mo_7_O_24_·4H_2_O). In the root compartment, the phosphate content of the medium was reduced to 1% (that is, 1%P MSR) of the abovementioned concentration (41 mg l^−1^ KH_2_PO_4_). We supplemented all media with 10 g l^−1^ sucrose and 3 g l^−1^ Phytagel. We verified that the addition of sucrose to the fungal compartment did not change the dynamics discussed in this Article (Extended Data Fig. 8). In the plates where carbon availability was doubled, the sucrose concentration in the media in the root compartment was increased to 20 g l^−1^ sucrose.

In a laminar airflow hood, we filled one compartment of a sterile two-compartment split plate with 28 ml MSR medium. We placed an autoclaved sheet of cellophane (Hoefer TE73, semi-circle with trapezoidal overhang at the straight edge) on top of the solidified medium. We then folded the cellophane overhang into the empty second compartment. We next inserted the custom acrylic frame into the empty compartment, securing the cellophane overhang between the acrylic frame, central barrier and the bottom of the plate. To avoid dislocation of cellophane and/or frame, we poured 5 ml 1%P MSR into the second compartment to immobilize the components. We then filled the compartment to a total of 25 ml 1%P MSR.

To quantify the saturation density and internal flow velocities of AM fungi in the absence of host roots, we used MSR medium with 0.3% (w/v) Phytagel as a growth substrate. For the myristate treatment, we supplied the medium with 0.5 mM myristic acid (Sigma-Aldrich; stock concentration 0.5 M in acetone). The control treatment lacking myristate (0 mM) received the same volume of acetone. After autoclaving, we filled 15 ml of medium in small Petri dishes (60 mm). Once fully cooled, we placed a circular sterile piece of porous cellophane (Hoefer TE73, 50 mm) onto the surface of the medium. The 0.5 mM concentration of myristic acid (C_14_H_28_O_2_) contributed a total of 1.3 mg of carbon to the 15 ml of medium in the plate, which was then inoculated with spores as described below.

### Inoculation of fungal material on root system

In a laminar airflow hood, we transferred 2–3 cm of in vitro Ri T-DNA transformed *D. carota* root (genotype 1 or 2) to the root compartment of the split plate. This compartment was not covered by cellophane. We then cut a circular plug containing only AM fungal mycelium and spores from fungal stock plates. We placed the inoculation plug on top of the root, covering around half of the root. We sealed the plates with parafilm and stored them horizontally and upright in an incubator at 25 °C. For AM fungi grown with myristate, we inoculated the centre of each plate with 10–15 spores in mixture of single spores and clusters without roots. In all cases, we checked plates regularly for growth and removed any roots crossing from the root compartment into the cellophane-covered fungal compartment. The AM fungi colonized the roots in roughly 30 days, crossing into the fungus-only compartment around 5–6 weeks after inoculation. For AM fungi grown without roots, we used a similar time frame (around 2 months). We recorded the time of the first barrier-crossing event, which was set as time zero, after which we began imaging network formation.

### Image processing

Details of image processing describing the segmentation of hyphal segments and spores as well as network graph extraction and node tracking can be found in Supplementary Methods.

### Analysis

#### Defining network features

*Defining the ROI*. To detect and describe all network features, we first defined the ROI for analysis within the fully stitched image of the fungal compartment. To avoid border effects, we defined the ROI as the area between a line that ran parallel to the central barrier of the split plate, separated by 6 mm from the barrier, and a 4.5 cm radius semicircle centred at the midpoint of that line within the plate.

*Classifying graph edges into BASs and RH*. We considered that a given edge of the network graph belonged to a BAS when any of these three criteria were met: (1) the length of the edge was less than 400 μm; (2) the length of the edge was less than 1,000 μm and one of the two end points of the edge coincided with the end point of a hypha (that is, a tip); (3) the average width of the edge was less than 7 μm and the product between this width and the length was less than 9,000 μm^2^. These choices were inspired by the definition of BASs according to a previous study^33^, with parameters tuned to achieve satisfactory BAS detection under visual evaluation. Edges that did not belong to a BAS were designated as belonging to an RH. The total length of RH shown in Fig. 4b is therefore the total length of all edges of the network minus the total length of BAS edges.

*Width estimates for BAS identification*. Although the low (×2) magnification used for network extraction does preclude accurate determination of hyphal width, we found that, to discriminate between RH and BAS, it was helpful to use crude width estimates obtained using the following procedure. First, transects perpendicular to the hyphal edge and of 120 pixels in extent were generated using the function profile_line of the package skimage^59^. We then fitted a Gaussian function to the resulting curve using the curve_fit function of the scipy.optimize^60^ package. Width was defined as two times the s.d.

*Growing tip definition*. Growing tips were defined as tracked nodes of degree 1 that were at least 40 pixels from their detected position in the last image frame where they were detected. Most network tips (degree 1 nodes) are non-growing BAS tips. Imperfect network alignment or extraction sometimes led to artefactual detection of growth in non-growing tips, which meant a non-zero speed was not a sufficiently robust criterion for detecting actively growing tips. It was therefore important to analyse non-growing BAS tips separately (see below).

*Classifying growing tips into BAS and RH*. Although the classification criteria for graph edges based on hyphal filament dimensions (see above) were sufficient for accurate estimation of total RH and BAS lengths, they did yield a finite rate of classification errors that tended to be higher near growing tips (because edges near RH tips had similar dimensions to BAS edges). Those errors had little effect on total length estimation but did significantly affect the estimation of growing tip density. Accurate estimation of growing tip density therefore required distinct classification criteria from those of edges. A growing tip was therefore designated as belonging to a RH if its final position was at a distance greater than a threshold distance, 2.5 mm, away from its initial position and all other growing tips were designated as belonging to BAS. The threshold was set by visually assessing the classification quality. As the model described the population of RH tips, we chose to plot in Figs. 2 and 3 the density of growing RH tips. Including all growing tips did not affect the travelling-wave dynamics discussed in the text.

*Detecting anastomosis (tip annihilation) events in space and time*. Anastomosis events occur when growing tips fuse with hyphal edges to create junctions across which cytoplasm is connected. Thus, every anastomosis event is also a tip annihilation event that contributes to regulating colony growth and densification behind the advancing wavefront. To compute the anastomosis rate plotted in Fig. 3, we first detected anastomosis events in our tracking analysis, where they were defined as the subset of all events at which a tracked node’s degree jumps from 1 to 3, whose degree never reverts back to degree 1 thereafter. The complementary subset whose degree does revert back to 1 were classified as hyphal crossing events. We recorded for each anastomosis event its position in space, defined by the pixel at which the skeletonized T-junction trifurcates, and its time, defined as the last timestep in which they were of degree 1. The anastomosis rate (in units mm^−2^ h^−1^) within a given ring at time *t* was computed by dividing the number of anastomosis events occurring within that ring over a time interval [*t*,*t* + Δ*t*] by the area of that ring and also by Δ*t*, the time interval between two successive frames. For the total anastomosis count (Fig. 4), the method was adjusted to study the evolving topology of the network (see the ‘Total anastomosis count’ section).

*Detecting branching events/newborn tips*. Newborn tips, which result from branching events, at each timestep *t* were defined as growing tips that appeared for the first time at *t*. To compute the branching rate plotted in Fig. 3, we first detected branching events in our tracking analysis. The branching rate (in units mm^−2^ h^−1^) within a given ring at time *t* was computed by dividing the number of branching events occurring within that ring over a time interval [*t*,*t* + Δ*t*] by the area of that ring and also by Δ*t*, the time interval between two successive frames.

*Distance from origin and travelling-wave speed*. We defined the distance from origin *r* by approximating the polygonal convex hull of the colony as a semicircle, and computing from the convex hull area *A* the semicircle radius *r* as $r=\sqrt{\frac{2A}{\pi }}$. The accuracy of this approximation for *r* is limited by the degree to which the convex hull of the colony is well approximated as a semicircle and varied across sample plates given the considerable random variation in colony shape. Inaccuracies in the estimate for *r* in turn leads to inaccuracies in the wave speed *v* estimated from the density profiles *n*(*r*) and *ρ*(*r*) at different times. For this reason, the growth speed of the ‘puller hyphae’ at the growing front *v*_p_ provides a more robust proxy for the travelling-wave speed, and was used to study the stability of wave speeds in Fig. 2 and Extended Data Figs. 1, 3, 4 and 8.

*Definition of ‘puller hyphae’ at the growth front*. At every timepoint *t*, we defined puller hyphae as those hyphae whose tip satisfies the definition for growing tips (see above), and in addition resides at the growth front at time *t*. Tips were defined to be at the growth front at timestep *t* if they were a vertex of the colony’s convex hull at both timesteps *t* and *t* + 1.

*Definition of the time coordinate*. In Figs. 2d, 3d and 5, zero on the time axis corresponds to the start time of imaging, which was initiated as soon as crossing into the fungal compartment was detected by manual examination of the pre-imaging sample pool. As those manual examinations were carried out once every 2 days, on average, the zero point on these time axes is therefore later than the actual crossing time by an unknown interval of up to 2 days. To compare temporal network development across samples in Fig. 4b–e, it was necessary to align in time the data from each sample. We therefore defined the offset time t* as the time at which the total RH length in the fungal compartment reached 100 mm, and plotted data from all samples as a function of *t* − t*.

*Definition of the arrival time* *t*_*n*_ *at the nth ring*. The arrival time *t*_*n*_ (used as a time offset in Fig. 2c) at which the travelling wave passes through the *n*th ring was defined as the time at which the front of density within that ring reached half of its maximal value. To obtain *t*_*n*_ for the hyphal density wave, we fit a sigmoid curve of equation $\rho (t)={K}_{1}\frac{1}{1+{{\rm{e}}}^{\lambda ({t}_{n}-t)}}$ to the hyphal density timeseries in the *n*th ring, with *K*_1_, *λ* and *t*_*n*_ as free parameters. For the tip density wave, we fit the curve of equation $n(t)={K}_{2}\frac{{{\rm{e}}}^{\lambda ({t}_{n}^{{\prime} }-t)}}{(1+{{\rm{e}}}^{\lambda ({t}_{n}^{{\prime} }-t)}{)}^{2}}$ with *K*_2_, *λ* and ${t}_{n}^{{\prime} }$ as free parameters.

*Total anastomosis count*. Whereas spatially resolved detection of anastomosis events for Fig. 3 achieved through tracking (see above) allowed us to estimate the rate of tip annihilation after formation of three-way nodes, the aim of the total anastomosis count across the entire network (Fig. 4) was to study the evolving topology of the network. As described in the Supplementary Methods, a fraction of anastomoses also occurred at crossing points (that is, degree 4 nodes), which do not lead to tip annihilation but might significantly affect the overall graph topology. We therefore used a different technique to detect anastomoses for the total-count analysis, based on graph theory. According to Euler’s formula for planar graphs, there is a relationship between the number of faces (which equals the number of anastomoses), the number of nodes *v* and the number of edges *e* and the number of faces *f*: *v* − *e* + *f* = 2. The number of edges and number of nodes were readily accessible from the extracted network graph. We found through high-magnification control experiments (Supplementary Methods) that around 10% of all anastomoses computed through the Euler formula corresponded to anastomoses at degree 4 nodes. Counting or not counting them does not substantially affect the overall picture, but we nevertheless included them in the anastomosis count (Fig. 4c).

### Analysis of flows

#### Kymograph generation

We recorded transport videos at high magnification (×100), as described in Supplementary Methods. We captured more than 1,600 videos at 20 or 25 fps for a minimum duration of 20 s across a total of 28 biological samples (split Petri plates). Within each biological sample, we recorded videos at different positions within the network (between 20 and 100 distinct positions per plate). We captured all videos within RH. We sampled flows as a function of spatial position across the network and this was done in a manner that follows particular hyphae (>90% of videos), as exemplified by the experiment shown in Fig. 5d. As these bright-field videos are label free, they effectively integrate information about the motion of any organelle or other biological object within the cytoplasm that produces sufficient intensity contrast. Most videos exhibited simultaneous antiparallel flows of such contrast objects (Supplementary Video 5), with some directed towards the host, that is, the root (Fig. 5a (green arrow)) and others toward the tip of the hypha (Fig. 5a (purple arrow)). For kymograph analysis, we analysed each video by first picking a linear (one-pixel wide) ROI of length 20 µm (Fig. 5a (white arrow)) at the centre of a straight section of the hypha to obtain at every image frame a (one-dimensional) vector of pixels **x**. We then arrayed **x** at each image frame to obtain a 2D image (kymograph) where one axis (shown vertically, in the example of Fig. 5a) represents the spatial dimension along **x** (of total length 20 µm) and the other axis (Fig. 5a (horizontal)) represents time (over the entire duration of the video, ranging from 20 s to 60 s). Example kymographs are shown in Extended Data Fig. 10, including kymographs from networks grown without a host in myristate (Extended Data Fig. 10). Given the label-free nature of the imaging performed, these kymographs represent a superimposition of many trajectories of individual contrast objects within the cytoplasm (detected generically as ‘particles’ in our speed analysis; see below) as their position along **x** evolves from frame to frame.

#### Speed extraction

We extracted one kymograph with multiple trajectories per video recorded, that is, a total of more than 1,600 kymographs to sample around 100,000 trajectories across a range of positions in space and times throughout network development. The kymograph extraction was done using MATLAB. We detected from each kymograph a set of individual trajectories by using a deep learning software for automated kymograph analysis (KymoButler, Wolfram Mathematica) developed previously^49^. This program works by using a fully convolutional deep neural network to identify bidirectional tracks to obtain from each kymograph a collection of particle trajectories. We imposed a minimum duration of ten consecutive frames as a constraint on the trajectory detection algorithm to ensure that detected trajectories correspond to the actual motion of contrast objects. Very short trajectories (below 500 ms in duration) could be associated with tracking errors across image frames that give rise to erroneous (and often anomalously high) flow speeds being detected. Imposing this constraint led to robust detection of correct flow speeds up to a limit of 40 or 50 µm s^−1^ (for videos recorded at 20 or 25 fps, respectively), corresponding to the speed of an object that travels the full spatial extent of the kymograph (20 µm) in 10 image frames. We checked all kymographs manually after the automatic detection to validate our protocol and confirm the accuracy of our detected speeds. Example kymographs are represented in Extended Data Fig. 10. As seen in Extended Data Fig. 10, detected trajectories demonstrated movements in both directions (toward the tip in purple and toward the root in green) for around 3,600 trajectories per biological sample). The statistics of their average velocities are represented in the violin plots of Fig. 5b.

#### Maximum-speed extraction

As the automatic detection of average velocities did not capture outliers (that is, very rapid flows larger than 40–50 µm s^−1^, mentioned above), we performed a manual screening of all recorded videos (more than 1,600 in number) to detect the fastest flow in each direction for each video. Once the fastest flows were identified within each video, the maximum velocity within the video was obtained by manually pointing at the slope of the corresponding trajectory (straight line) within the associated kymograph. We then obtained maximum velocities for each video as represented in Fig. 5c.

Over the entire set of over 1,600 videos investigated, only 7% of the videos (<120 in number) included maximum-speed outliers that were not detected automatically by the algorithm. This relatively low incidence of undetected outliers provided additional confidence that the average speed statistics represented from the automated detection (Fig. 5b) are reliably representative.

#### Spatial mapping within the network

We next mapped the spatial position of each flow video to the skeletonized network to enable analyses of flow velocities as a function of space, as seen in Fig. 5d–f. We recorded the acquisition *x*–*y* coordinate within the sample plate for each high-magnification flow video (using a ×100 objective, image size of 141 µm × 103 µm at the sample plane), and we aligned the full set of these coordinates for each plate with the network skeleton (extracted from the stitched image of the fungal compartment obtained with a ×2 objective) leading to a rough overlap. This first alignment was achieved by matching the *x*–*y* coordinates of one particular video (such as an easily identifiable tip) within the skeleton. We then manually performed a finer adjustment of the position of each video within the network by comparing the exact shape of each hypha imaged with the skeleton. The maximum error for alignment was around 100 µm (of the same order of magnitude as the size of the high-magnification field of view).

#### Distance to the tip

The distance to the tip corresponds to the curvilinear length to the tip of the hypha to which a given video position belongs—not the closest tip including other hypha of the network. A hypha is an equivalence class on the set of edges based on the continuity relationship. In brief, two edges belong to the same hypha if they are the trace left by the same growing tip. In practice hyphae can be recognized by a continuity in edge directionality and width at each junction. On the basis of visual identification, we manually defined which hypha each edge in a given video belonged to and therefore with which tip of the hypha it was associated. Distances are defined by the curvilinear length along the graph through the shortest path that goes from the video position to the tip.

### Plate harvesting and DNA extraction

The roots were harvested from the split plate using tweezers, removing any trace amounts of media at 1 (*n* = 3), 3 (*n* = 5), 7 (*n* = 3), 12 (*n* = 4) and 30 (*n* = 8) days after *R. irregularis* A5 crossed to the fungal compartment. The total wet weight of the roots was measured and split for DNA extraction and root staining to determine colonization. The roots separated for DNA extraction were placed to dry in an oven in a paper bag at 80 °C and the dry weight was measured after 72 h.

The samples were first ground using liquid nitrogen and a mortar and pestle to disrupt the cells. DNA was extracted using the Qiagen DNeasy PowerSoil kit according to manufacturer’s instructions. One change was made in the duration of the first centrifugation step, lengthened from 30 s to 3 min to better separate the supernatant. DNA was eluted into 50 µl 10 nM Tris-HCl, pH 8.5, and quantified using the Nanodrop Spectrophotometer ND-1000. For phosphorus content determination of roots and agar, see the Supplementary Methods.

### Intraradical mycelium quantification

To quantify intraradical length, we used two methods: (1) one traditional method relying on root staining and visual quantification (that is, the Trouvelot method)^61^; and (2) one method using droplet digital PCR (ddPCR)^62,63^ to calculate the number of nuclei and then convert it to length of hyphae based on our calculated nuclear density per µm of hyphae (Supplementary Methods). We used ddPCR with fluorescent probes specific to AM fungi, targeting sequences that occur only once per nucleus (that is, the single-copy MAT gene) to directly quantify the total number of nuclei across the hyphae^64^. For each plate, four non-template controls and four positive controls (*R. irregularis*, A5 DNA extracted from pure culture) were used to set the fluorescence amplitude threshold (high threshold) values to distinguish between the positive and negative droplet cloud. Once thresholds were set, we used the concentration (copies per µl) to calculate the length of hyphae using a modified formula from^63^. To calculate the length of the hyphae in the roots (µm), this final value was then multiplied by the average distance between nuclei as described in the Supplementary Methods, Extended Data Fig. 6 and Supplementary Video 8.

### Statistics and reproducibility

#### Network analyses

For Fig. 3b and Extended Data Fig. 12e–g, ring-frame temporal profiles were computed over *n*_ring_ = 15 rings, sampled across the same growing network.

For Fig. 3c, speed histograms were computed from *n*_pink_ = 1,645 and *n*_cyan_ = 103 growing tips.

For Extended Data Figs. 1b, 3b, 4b and 8b, datapoints represent the mean over the *n* growing tips at the front observed at each timepoint. The value of *n* fluctuated in the range 1 ≤ *n* ≤ 21, with 77% of all timepoints in the range *n* ≥ 3.

For Extended Data Fig. 2, the number of samples for each data series is given by *p*/*t*/*n* where *p* is the panel label, *t* indicates genotype and/or treatment, and *n* is the number of independent biological replicates: a/A5-100C/10, a/A5-200C/2, a/C2-100C/5 a/C2-200C/3, a/Agg-100C/4, b/AMF/23, b/free-living/25, c/A5-100C/20, c/A5-200C/13, c/C2-100C/11 c/C2-200C/12, d/A5-100C/10, d/A5-200C/2, d/C2-100C/5 d/C2-200C/3, e/genotype-1/22, e/genotype-2/6, f/genotype-1/27f/genotype-2/18, h/symbiotic/23, h/myristate/5.

Extended Data Figure 11a was computed from 118 hyphal growth trajectories, and Extended Data Fig. 11b was computed from 71 RH and 881 BAS branch points, all sampled from the same growing network.

#### Flow analyses

For Fig. 1b, we observed speeds between 2 and 5 μm s^−1^ in 43 videos acquired within the time interval *t* = 0.5–1.5 days, speeds between 5 and 10 μm s^−1^ in 26 videos acquired within *t* = 1.5–2.5 days and speeds between 15 and 45 μm s^−1^ in 8 videos acquired within *t* = 2.5–3.5 days.

For Fig. 5b, each pair of violin plots in the top panel (for positive and negative velocities) and points in the bottom panel (for the absolute ratio of positive and negative means) appearing at the same *x* coordinate corresponds to data from an independent biological replicate. The number of samples for violins at each *x* coordinate is given by *x*/*p*/*r*/*k*, where *x* is the timepoint (in days) at which that biological replicate was measured (*x* coordinate), *p* is the count of trajectories in the tipward direction, *r* is the count of trajectories in the rootward direction and *k* is the number of videos from that replicate: 0.4/464/494/12, 0.6/989/387/29, 1.4/527/516/13, 1.5/306/555/7, 2.3/774/941/22, 2.4/686/1,092/19, 3.3/402/872/23, 3.3/969/1,160/24, 3.4/1,678/1,060/54, 3.4/1,553/2,332/50, 4.4/592/1,084/24, 4.4/673/865/18, 4.6/1,475/1,842/52, 8.4/4,959/4,594/158, 10.6/677/614/22, 12.4/1,817/1,823/22, 13.3/825/1,546/16, 14.4/517/1,365/13, 15.3/1,223/1,284/22, 16.4/1,034/987/26, 17.5/2,468/1,373/53, 20.4/2,689/2,342/73, 21.4/9,595/8,414/284. In total, about 75,000 trajectories from approximately 1,200 videos were used.

For Fig. 5c, each pair of violin plots (for positive and negative extreme velocities) appearing at the same *x* coordinate corresponds to data from an independent biological replicate, except at *x* = 0, where 11 biological replicates had the same measured value along the *x* coordinate. The number of samples for violins at each *x* coordinate is given by *x*/*p*/*r*/*k*, where *x* is the number of spores measured at that *x* coordinate, *p* is the count of trajectories in the tipward direction, *r* is the count of trajectories in the rootward direction and k is the number of videos from that replicate: 0/8,060/9,026/309, 15/1,475/1,842/58, 38/1,223/1,284/32, 39/517/1,365/21, 60/1,553/2,332/55, 67/1,034/987/31, 72/2,494/2,437/54, 73/4,959/4,594/177, 84/825/15,46/27, 249/9,595/8,414/332, 380/2,689/2,342/81, 857/2,468/1,373/59 In total, about 1,200 videos were used. For each video, only the maximum value of all trajectories is used for the violin plot.

For Fig. 5e, each pair of violin plots (for positive and negative velocities) appearing at the same *x* coordinate corresponds to data from one kymograph, from a set of videos sampled across network locations indicated in Fig. 5d. The number of samples for each violin is given by *x*/*p*/*r*, where *x* is the distance to the tip (*x* coordinate), *p* is the count of trajectories in the tipward direction and *r* is the count of trajectories in the rootward direction: 0.01/3/2, 1.02/1/0, 1.73/1/0, 2.31/2/1, 2.79/1/0, 3.78/1/29, 3.78/1/29, 3.78/1/29, 3.78/1/29, 6.33/1/43, 6.33/1/7, 6.33/60/43, 6.33/60/7, 6.87/64/35, 6.87/4/35, 7.85/0/4, 8.64/5/10, 9.22/48/94, 9.22/48/36, 9.22/5/94, 9.22/5/36, 12.0/4/43, 12.0/4/14, 12.0/35/43, 12.0/35/14, 12.72/24/74, 12.72/24/29, 12.72/45/74, 12.72/45/29, 14.57/21/40, 14.57/14/35, 14.57/14/40, 14.57/21/35, 14.94/2/44, 14.94/2/24, 14.94/4/44, 14.94/4/24, 17.19/40/32, 17.19/50/32, 17.19/50/32, 17.19/40/32, 17.89/23/46, 17.89/27/39, 17.89/27/46, 17.89/23/39, 20.53/2/79. In total, 824 trajectories from 27 kymographs were used. Each violin corresponds to one video.

For Fig. 5f, each pair of violin plots (for positive and negative velocities) appearing at the same *x* coordinate corresponds to data from one kymograph, from a set of videos sampled across network locations indicated in Fig. 5d. The number of samples for each violin is given by *x*/*p*/*r*, where *x* is the BC (*x* coordinate), *p* is the count of trajectories in the tipward direction and *r* is the count of trajectories in the rootward direction: 0.01/1/0, 0.01/3/2, 0.06/1/0, 0.07/1/0, 0.07/2/1, 0.08/1/29, 0.08/1/29, 0.08/1/29, 0.08/1/29, 0.09/64/35, 0.09/4/35, 0.18/0/4, 0.21/60/43, 0.21/60/7, 0.21/1/43, 0.21/1/7, 0.21/5/10, 0.25/5/94, 0.25/5/36, 0.25/48/94, 0.25/48/36, 0.37/4/43, 0.37/4/14, 0.37/35/43, 0.37/35/14, 0.42/40/32, 0.42/50/32, 0.42/40/32, 0.42/50/32, 0.43/2/79, 0.47/24/74, 0.47/24/29, 0.47/45/74, 0.47/45/29, 0.5/23/46, 0.5/27/39, 0.5/27/46, 0.5/23/39, 0.58/14/35, 0.58/14/40, 0.58/21/35, 0.58/21/40, 0.6/2/44, 0.6/2/24, 0.6/4/44, 0.6/4/24. In total, 824 trajectories from 27 kymographs were used. Each violin corresponds to one video.

For Fig. 5b–f, all violin plots plotted using violinplot function of matplotlib Python library with the parameter show_extrema set to false. All linear fits computed using regplot function of seaborn Python package^65^. It computes the regression line and shows a 95% confidence interval as a shaded area around this regression line.

For Extended Data Fig. 7c, for all box plots, the number *n* of independent biological replicates was *n* = 7.

For Extended Data Fig. 10g, violin plots were constructed from *n* trajectories from *p* videos sampled across *k* independent biological replicates. For networks connected to host roots *n* = 71,009, *p* = 113, *k* = 11; for networks in non-symbiotic context *n* = 2,450, *p* = 86, *k* = 7. We excluded immotile objects, which we defined as trajectories demonstrating displacements indistinguishable from diffusion, demonstrating speeds below a threshold of 0.8 μm s^−1^ corresponding to the average speed of a one pixel wide (*r* = 35 nm) particle diffusing for Δ*t* = 20 s (video length) in water ($D=\frac{{k}_{b}T}{6\pi \eta r},v=\sqrt{\frac{D}{\Delta t}}$). This lead to excluding about 50% of the trajectories in the case of myristate, where flows were generally less active.

#### Intraradical colonization imaging

For Extended Data Fig. 6b–f, imaging with DAPI staining was done on three independent plates on three different days showing similar results.

#### Bootstrap resampling uncertainties

For Fig. 2b, we obtained uncertainty estimates for hyphal and growing tip density by splitting each ring in which densities were computed into a set of 10,000 rectangles of equal area, computing the densities in each of the rectangular area separately, and estimating the s.d. of the mean density by bootstrap resampling (sampling with replacement) 100 times over the set of rectangular area densities. The shaded regions correspond to two times the s.d. of the bootstrap resampling.

For Extended Data Figs. 1a, 3a, 4a and 8a, as the bootstrapping procedure used for Fig. 2c is computationally costly, for these Extended Data figures on replicates we estimated uncertainties in the density by assuming scaling relations based on relative magnitudes of uncertainties in Fig. 2b. Specifically, we used *σ*_*ρ*_ = 160 μm mm^−2^ × *ρ*/*ρ*_sat_ for the filament-density uncertainty and σ_*n*_ = *n*/4 for the tip-density uncertainty. The shaded regions correspond to the mean ± *σ*_*ρ*__,__*n*_.

For Fig. 2c,d, the shaded region is the confidence interval obtained by bootstrap resampling 1,000 times the sigmoid fit of each density profile in each ring reference frame. A function $\rho (t)={K}_{1}\frac{1}{1+{{\rm{e}}}^{\lambda ({t}_{n}-t)}}$ was fitted to the resampled hyphal density timeseries in the *n*-th ring, with *K*_1_, *λ* and *t*_*n*_ as free parameters. The grey region in Fig. 2c,d shows, respectively, the interval around the mean value of *K*_1_, *t*_*n*_ ± 2 times the standard error of the bootstrap estimates of these parameters.

### Reporting summary

Further information on research design is available in the Nature Portfolio Reporting Summary linked to this article.

## Online content

Any methods, additional references, Nature Portfolio reporting summaries, source data, extended data, supplementary information, acknowledgements, peer review information; details of author contributions and competing interests; and statements of data and code availability are available at 10.1038/s41586-025-08614-x.

## Supplementary information

Supplementary Information Supplementary Methods, Supplementary Discussion and Supplementary References

Reporting Summary

Supplementary Video 1 Extracted skeleton of the network development over the first 112 h. The colour of each segment corresponds to time of appearance.

Supplementary Video 2 Tracking of nodes in a region of space. The node colour corresponds to their label, maintained over time. The edge colour corresponds to label, maintained over time when the two end nodes are constant. Scale bar, 1 mm.

Supplementary Video 3 Example high-magnification video of flow trajectories of *R. irregularis* A5. Scale bar, 10 μm.

Supplementary Video 4 Travelling wave of hyphal filaments and tip densities. Blue is filament density and red is tip density. The shaded region corresponds to uncertainty in density estimates computed using bootstrapping.

Supplementary Video 5 Example high-magnification video of flow trajectories of *R. irregularis* A5. Videos correspond to kymographs of Extended Data Fig. 10a–e. Scale bar, 10 μm.

Supplementary Video 6 High-magnification video associated with Fig. 5a. Scale bar, 10 μm.

**Extended Data Fig. 9:**
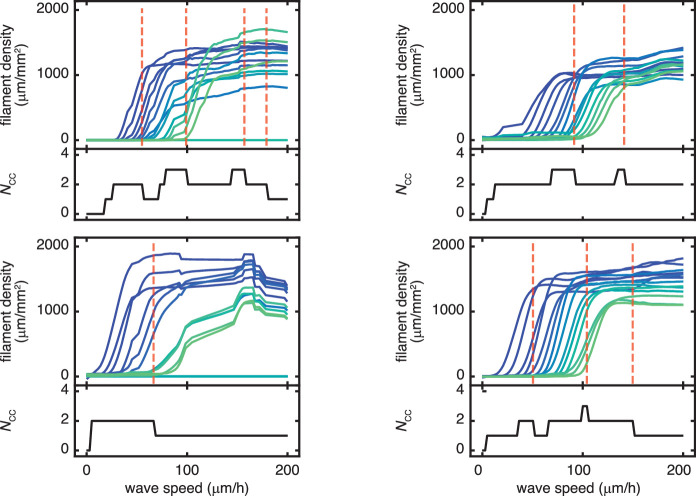
Quantitative analysis of colliding waves in plates with multiple crossing events (Supplementary Video 7). The position of the four panels within the 2 × 2 array above reflects the position of the corresponding video within the SI video 7. Within each panel, the upper subpanel shows a family of density time series, one for each successive concentric rings of increasing radius, represented by blue to green gradient (blue = small, green = large). No effects on the density time series are observed upon collision events (red lines), which are detected as decreases in the number of connected components ${N}_{\text{CC}}$ waves (lower subpanel) representing the number of disjoint waves that have yet to collide. This outcome is consistent with our model: when waves collide, networks continue to cover the available space until the tips anastomose into an existing network upon reaching saturation density.

Supplementary Video 7 Example colliding wave videos corresponding to Extended Data Fig. 9. Scale bar, 1 cm.

Supplementary Video 8 Imaging of nuclei in fixed mycelia of the strain *R. irregularis* A5. Scale bar, 20 μm; corresponding to Extended Data Fig. 6.

**Extended Data Fig. 10:**
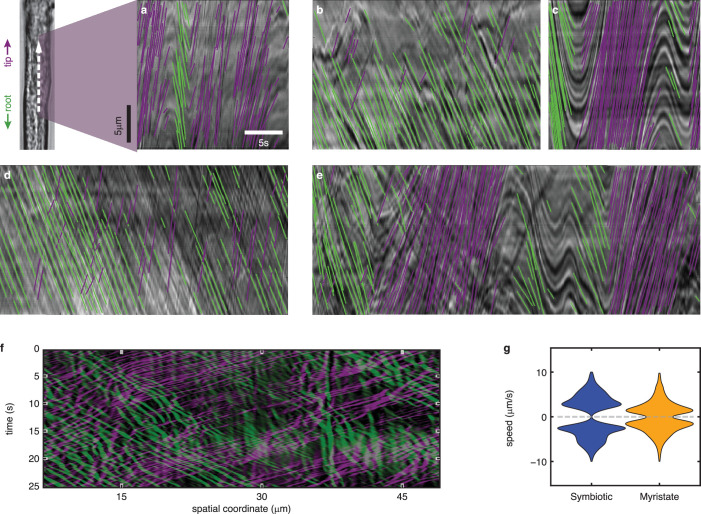
Example kymographs showing the automatic detection of flow velocities across different ages and conditions. Representation of five kymographs from five different biological samples of *R. irregularis* of age: (**a**) 1 day, (**b & c**), 4 days, (**d**) 5 days and (**e**) 11 days after crossing. Vertical axis represents space (20 µm) while horizontal axis represents time (from 20 s to 60 s). Averaged velocities automatically detected are represented by coloured straight lines. Scale bars in (**a**) are valid for all panels. Trajectories in both directions, towards the tip (purple) and the root (green), were detected in most of the 1600 videos acquired. The density of detected trajectories fluctuated substantially across samples (as seen by the differences between (**a**) and (**b**) for instance). Flow patterns across time were also diverse – for example, the reversal events prominent in (**a**), (**c**), (**e**) are not observed in (**b**) or (**d**). Corresponding videos can be found in (Supplementary Video 5). (**f-g**) Flow patterns inside networks of *R. irregularis* strain A5 when grown in the absence of a host root. (**f**) Example kymograph from a video of flows of a R. irregularis A5 network grown on myristate rather than with a host root shows bidirectional movement. (**g**) Distribution of observed velocities in two directions measuring flows from symbiotic context, in which networks are connected to host roots (blue), and distribution of flow speeds observed of networks grown in non-symbiotic context with myristate (orange). In both cases, we excluded the immotile fraction of tracked objects, defined as those demonstrating displacements indistinguishable from diffusion (see Methods).

**Extended Data Fig. 11:**
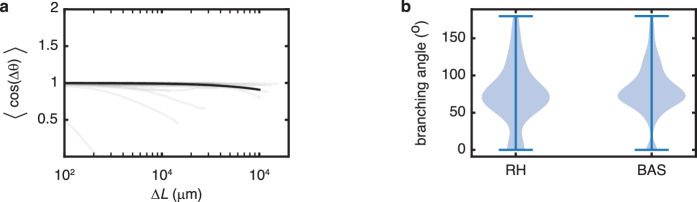
Individual hyphal filaments grow with high directional persistence but form branches over a distribution of angles. (**a**) For each individual hyphal filament, persistence of the growth direction is shown by plotting the average cosine of the angular deviation $\langle \cos (\Delta \theta (\Delta L))\rangle$ computed for different lags in length $\Delta L$. Under the assumption that the expectation value of the cosine of the angle falls off exponentially with distance we have $\langle \cos (\Delta \theta (\Delta L))\rangle ={e}^{-\Delta L/{{\ell }}_{p}}$ where ${{\ell }}_{{\rm{p}}}$ is the persistence length of the growing filament and the averaging is done over all the starting positions along the hypha. Grey lines show the decay of $\langle \cos (\Delta \theta (\Delta L))\rangle$ as a function of $\Delta L$ for each individual hypha. The median persistence length of all hyphae is $\mathop{{\ell }_{{\rm{p}}}}\limits^{ \sim }\approx 10$ cm. The corresponding median decay ${e}^{-\Delta L/\mathop{{\ell }_{{\rm{p}}}}\limits^{ \sim }}$ is shown as a thick black line. Note that given that the orientation correlation $\langle \cos (\Delta \theta (\Delta L))\rangle$ barely decays over the entire tested range, these persistence lengths estimated from exponential fits should be interpreted with care, and likely represent lower bounds on the true persistence length. (**b**) Distribution of absolute branching angle of new-born runner hyphae and new-born branched absorbing structures. Angles above 100° can be an artefact of the extraction. On average RH branch at an angle of 73° from the initial growth direction and BAS branch at an angle of 80°. These two values are not statistically significantly different.

**Extended Data Fig. 12:**
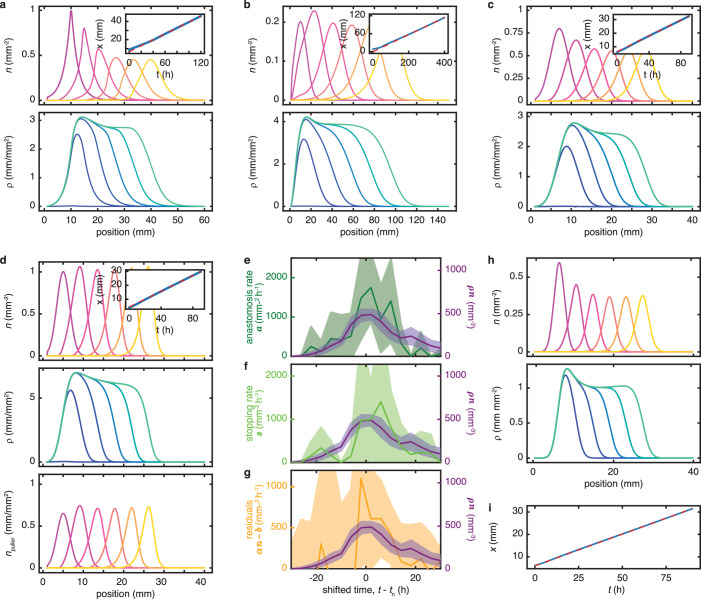
Numerical simulations of BARE wave model variants. (**a-c**) Variants with different assumptions about the spatial flux ${\boldsymbol{J}}(n)$ underlying the wave speed $c$ (see Supplementary Discussion section 4.3.1). (**a**) ${\boldsymbol{J}}(n)=-\,nv\hat{{\boldsymbol{r}}}$ with $\alpha =$ 0.039 h^−1^, *β* = 22 μm h^−1^, *v* = 200 μm h^−1^. Simulated wave speed was *c* = 330 μm h^−1^. Colour gradient: equally spaced intervals from t = 0 h to t = 120 h. (**b**) ${\boldsymbol{J}}(n)=D{\rm{\nabla }}n$ with *α* = 0.039 h^−1^, *β* = 22 μm h^−1^, and D = 0.55 mm^2^ h^−1^, *v* = 200 μm h^−1^. Simulated wave speed was *c* = 280 μm h^−1^. Colour gradient: equally spaced intervals from t = 0 h to t = 400 h. (**c**) ${\boldsymbol{J}}(n)=-\,n{v}_{d}\hat{{\boldsymbol{r}}}+D\nabla n$ with $\alpha$ = 0.039 h^−1^, ${v}_{d}$ = 235, *β* = 22 μm h^−1^, D = 0.02 mm^2^ h^−1^ and *v* = 200 μm h^−1^. Simulated wave speed was $c$ = 280 μm h^−1^. Colour gradient: equally spaced intervals from t = 0 h to t = 90 h. (**d**) Alternative model with explicit representation of puller hyphae (Eq. (7) of Supplementary Discussion), and ${\boldsymbol{J}}(n)=-\,n{v}_{d}\hat{{\boldsymbol{r}}}$ with $\alpha$ = 0.039 h^−1^, *v*_*d*_ = 220 μm h^−1^, *β* = 22 μm h^−1^, *v* = 220 μm h^−1^, ${K}_{1}=0.25\,{{\rm{h}}}^{-1},\,{K}_{2}=0.40\,{{\rm{h}}}^{-1}$ and *v*_p_ = 300 μm h^−1^. Simulated wave speed was *c* = 280 μm h^−1^. Colour gradient: equally spaced intervals from t = 0 h to t = 90 h. Insets in (**a-d**): position of simulated tip density peak over time (blue line), with linear fit (Red dashed line) that yields wave speed $c$. (**e-i**) Variant with refined density control that accurately predicts saturation density (see Supplementary Discussion 4.3.3). (**e-g**) Temporal profile in the ring reference frame for key density-dependent quantities (shaded areas: mean $\pm 2\times$ s.e.m.). Within the same measured network, the (nonlinear) density product $n{\rho }^{2}$ (purple line) is compared against anastomosis rate $a$ (**e**), rate of tip annihilation by stopping $s$ (**f**), and the residual $\alpha n-b$ quantifying the error in the linear form $b(n)=\alpha n$ (as in Fig. 3b) approximating the observed branching rate $b$. Good overall agreement between the pairs of curves in (**e-g**) suggests modelling $a$, $s$, and $\alpha n-b$ each proportional to ${n\rho }^{2}$, with coefficients ${\beta }^{{\prime} }$, ${\beta }_{\text{s}}^{{\prime} }$, ${\alpha }^{{\prime} {\prime} }$, respectively (see Supplementary Discussion 4.3.3). Combining these three processes we obtain refined expressions for the branching and annihilation rates: $b(n,\rho )=\alpha n+{\alpha }^{{\prime} {\prime} }n{\rho }^{2}$ and $a(n,\rho )=({\beta }^{{\prime} }+{\beta }_{\text{s}}^{{\prime} })n{\rho }^{2}$, respectively. Plugging into the BARE wave model and solving yields ${\rho }_{\text{sat}}=\sqrt{3\alpha /\gamma }=1.3\,{{\rm{mm}}}^{-1}$ mm^−1^, where ${\gamma \equiv \beta }^{{\prime} }+{\beta }_{\text{s}}^{{\prime} }-{\alpha }^{{\prime} {\prime} }$. From the data in (**e-g**), $\gamma \approx 0.075$ mm^2 ^h^−1^, leading to ${\rho }_{\text{sat}}\approx 1.3$ mm^−1^ – very close to the experimentally observed ${\rho }_{\text{sat}}\approx 1$ mm^−1^. (**h**,**i**) Simulations of BARE wave model with those refined rate expressions $b(n,\rho )$ and $a(n,\rho )$, demonstrating that these refinements do not compromise the existence of travelling-wave solutions with constant wave speed. We used $\gamma =0.075$ mm^2 ^h^−1^, and all other parameters as in (**c**). In (**a-d**,**h-i**), initial conditions were $\rho (r,t=0)=0$ and $n(r,{\rm{t}}=0)={n}_{\max }{e}^{-{\rm{\lambda }}{{\rm{|x}}-{x}_{0}|}^{k}}$, with parameters ${n}_{\max }$/ ${\rm{\lambda }}$ / ${x}_{0}$/$k$ (in units ${{\rm{m}}{\rm{m}}}^{-2}$/${{\rm{m}}{\rm{m}}}^{-k}$ /${\rm{m}}{\rm{m}}$/dimensionless): (**a**) 1/0.4/10/1; (**b**) 0.2/0.015/10/2; (**c**) 0.8/0.1/7/2; (**d**) 1/0.2/5/2 ($n$) 0.6/0.2/5/2 (${n}_{\text{puller}}$); (**h**) 0.6/0.3/7/2.

Supplementary Video 9 High-magnification video of flow in hyphae of *R. irregularis* in the absence of host root with carbon supplied as myristate corresponding to Extended Data Fig. 10f.

## Data Availability

All source data required to reproduce the main analysis as well as all main figures and extended data figures are available at Figshare (10.6084/M9.FIGSHARE.27889143)^66^.

## Code availability

Custom code was developed for the purpose of analysis. All custom code and scripts to reproduce the main analysis of this paper are available under MIT licence at GitHub (https://github.com/Cocopyth/AMFTravellingWave) and a frozen version is provided at Figshare (10.6084/M9.FIGSHARE.27889143)^66^.
