# Supplementary material for: A travelling-wave strategy for plant–fungal trade
Source: Nature. 2025 Feb 26;639(8053):172–80. doi: 10.1038/s41586-025-08614-x (PMC11882455; doi:10.1038/s41586-025-08614-x)
Supplement: Supplementary file 1 — Supplementary Methods, Supplementary Discussion and Supplementary References [file 41586_2025_8614_MOESM1_ESM.pdf]

nature portfolio

https://doi.org/10.1038/s41586-025-08614-x

Supplementary information

A travelling-wave strategy for plant–fungal trade

In the format provided by the authors and unedited

Supplementary Information: A traveling-wave strategy for plant-fungal trade

Loreto Oyarte Galvez[1,2+], Corentin Bisot[2,3+], Philippe Bourrianne[4,5], Rachael Cargill[1,2], Malin Klein[1,2], Marije van Son[1], Jaap van Krugten[2], Victor Caldas[1,2], Thomas Clerc[2], Kai-Kai Lin[2], Félix Kahane[2], Simon van Staalduine[2], Justin D. Stewart[1,6], Victoria Terry[1], Bianca Turcu[1], Sander van Otterdijk[1], Antoine Babu[2], Marko Kamp[2], Marco Seynen[2], Bas Steenbeek[2], Jan Zomerdijk[2], Evelina Tutucci[1], Merlin Sheldrake[1,6], Christophe Godin[3], Vasilis Kokkoris[1], Howard A. Stone[4*], E. Toby Kiers[1,6*] and Thomas S. Shimizu[2*]

[1]Amsterdam Institute for Life and Environment, Vrije Universiteit, Amsterdam, Netherlands

[2]AMOLF Institute, Amsterdam, Netherlands

[3] Laboratoire Reproduction et Développement des Plantes, Univ Lyon, ENS de Lyon, UCB Lyon 1, CNRS, INRAE, INRIA, F-69342, Lyon, France

[4]Department of Mechanical and Aerospace Engineering, Princeton University, USA

[5]PMMH, CNRS, ESPCI Paris, Université PSL, Sorbonne Université, Université Paris Cité, France

[6]Society for the Protection of Underground Networks, SPUN, Dover, DE 19901, USA

+ these authors contributed equally to this work

*co-corresponding authors

TABLE OF CONTENTS

1 List of Supplementary Videos 2
2 List of Extended Data Figures 3
3 Supplementary Methods 4
3.1 Timelapse Imaging 4
3.2 Image processing 4
3.2.1 Image stitching 4
3.2.2 Network extraction 4
3.2.3 Network Overlay 5
3.2.4 Graph generation 5
3.2.5 Node tracking 5
3.2.6 Spore extraction 6
3.3 Spatial averaging 6
3.3.1 Definition and use of rings for density mapping 6
3.3.2 Hyphal length definition 6
3.3.3 Area definition 7
3.4 Microscopic network connectivity 7
3.4.1 Connectivity of overlapping hyphae (degree 4 nodes) 7
3.4.2 Connectivity of anastomosis and crossing points 7
3.5 Phosphorus Measurement 7
3.5.1 Agar and root prep 7
3.5.2 Digestion 7
3.6 Intraradical colonization quantification 8
3.6.1 Counting nuclei 8
3.6.2 Total length of intraradical hyphae 8

3.6.3 Colonized root imaging ... 9
3.7 Graph metrics ... 9
3.7.1 Betweenness Centrality ... 9
3.7.2 Global and root efficiency ... 10
3.8 Transport imaging ... 11
3.8.1 Imaging system ... 11
3.8.2 Spatial mapping of videos in the network ... 11
Fourier filtering of kymographs ... 11
3.8.3 Hyphal radius determination ... 11
4 Supplementary Discussion ... 12
4.1 Dimensionality (2-D vs 3-D) and geometry of mycorrhizal growth ... 12
4.2 Branching and annihilating range expansion (BARE) wave model ... 12
4.2.1 General model description ... 12
4.2.2 Expressions for $\boldsymbol{bn}, \boldsymbol{\rho}$ and $\boldsymbol{an}, \boldsymbol{\rho}$ ... 13
4.2.3 Travelling wave solutions of the BARE wave model yield "pulled waves" ... 14
4.2.4 Evidence from experiments and parameter fitting ... 15
4.2.5 BARE wave model with P absorption ... 15
4.2.6 Conclusion ... 16
4.3 Further discussion of the BARE wave model, its refinement, and alternative models ... 17
4.3.1 Choice of $\boldsymbol{Jn}$ ... 17
4.3.2 Other models ... 18
4.3.3 Refinement of morphogenetic terms to match the observed saturation density ... 19
4.3.4 Estimating carbon cost ... 21
4.4 Numerical simulations of traveling-wave models ... 21
4.5 Linking modelling and experimental parameters ... 22
4.5.1 Parameter extraction from curve proportionality ... 22
4.5.2 Stopping rate ... 22
4.5.3 Total hyphal density ... 22
5 Supplementary References ... 23

1 LIST OF SUPPLEMENTARY VIDEOS

Supplementary Video 1: Extracted skeleton of the network development over the first 112 hours. Colour of each segment corresponds to time of appearance.

Supplementary Video 2: Tracking of nodes in a region of space. Node colour corresponds to their label, maintained over time. Edge colour corresponds to label, maintained over time when the two end nodes are constant. Scale bar is 1 mm.

Supplementary Video 3: Example high magnification video of flow trajectories of *Rhizophagus irregularis* A5. Scale bar is $10\mu m$.

Supplementary Video 4: Traveling wave of hyphal filaments and tip densities. Blue is filament density and red is tip density. Shaded region corresponds to uncertainty in density estimates computed via bootstrapping.

Supplementary Video 5: Example high magnification video of flow trajectories of *Rhizophagus irregularis* A5. Videos correspond to kymographs of **Extended Data Fig. 10a-e**. Scale bar is $10\mu m$.

Supplementary Video 6: High magnification video associated with **Fig. 5a.** Scale bar is $10\mu m$.

Supplementary Video 7: Example colliding wave videos corresponding to **Extended Data Fig. 9.** Scale bar is 1cm.

Supplementary Video 8: Imaging of nuclei in fixed mycelia of the strain *Rhizophagus irregularis* A5. Scale bar is $20\mu m$ corresponding to **Extended Data Fig. 6**

Supplementary Video 9: High magnification video of flow in hyphae of *Rhizophagus irregularis* in the absence of host root with carbon supplied as myristate corresponding to **Extended Data Fig. 10f**

2 LIST OF EXTENDED DATA FIGURES

Extended Data Fig. 1: Replicates of *R. irregularis* A5 plates show consistent traveling wave pattern

Extended Data Fig. 2: Saturation density varies across AM fungal genotypes but is invariably lower than that of free living fungi. AM fungi consistently regulate network structure despite changes in carbon supply, root genotype, or even absence of a host root with carbon supplied as myristate. Total number of spores across the network jumps rapidly at variable times, and then grows more gradually.

Extended Data Fig. 3: Replicates of *R. irregularis* C2 plates show consistent traveling wave pattern

Extended Data Fig. 4: Replicates of *R. aggregatum* plates show consistent traveling wave pattern

Extended Data Fig. 5: Pattern of spatial phosphorus (P) absorption aligns with regulated fungal wave growth.

Extended Data Fig. 6: Intraradical growth and colonization increase concurrently with extraradical growth

Extended Data Fig. 7: Flow statistics correlate Betweenness Centrality (BC) hierarchy and indicates incompressibility

Extended Data Fig. 8: Travelling wave dynamics do not differ between the sucrose and no sucrose treatments in the fungal compartment

Extended Data Fig. 9: Quantitative analysis of colliding waves in plates with multiple crossing events

Extended Data Fig. 10: Example kymographs of *R. irregularis* strain A5 showing the automatic detection of flow velocities across different ages. Flow patterns inside networks of *R. irregularis* strain A5 when grown in the absence of a host root.

Extended Data Fig. 11: Individual hyphal filaments grow with high directional persistence but form branches over a distribution of angles

Extended Data Fig. 12: Numerical simulations of model variants with different assumptions about the spatial flux J(n) underlying the wave speed *c*. Traveling wave model with refined density control accurately predicts saturating density

3 SUPPLEMENTARY METHODS

3.1 TIMELAPSE IMAGING

After the fungal network entered the fungus-only compartment, we placed each replicate into the sample rack of our custom-built, high-throughput imagining robot. This allowed us to generate high-resolution images of the fungal network every 1, 2 or 4 hours, for ~15 days, and every 8 hours for the next 30 days. The imaging platform was temperature controlled (25 C) in a dark enclosed environment. Our optical system integrated an illumination light source, lenses, and camera, which translated across the samples, allowing imaging of up to 40 plates in parallel. Our customized microscope was composed of a 2x objective lens (Thorlabs TL2X-SAP - 2X 0.1 NA Super Apochromatic) coupled through a tube lens (f=200mm) to a 12MP CMOS camera (Basler acA4112-30um) with a pixel size 1.725um small enough to achieve Nyquist sampling of the optical resolution, which we estimated according to Rayleigh criterion at $0.61\lambda/NA \approx$3.7um, given the objective's numerical aperture (NA=0.1) and illumination wavelength ($\lambda \approx$600nm). The objective, tube lens, and camera were mounted altogether on a Thorlabs KMTS25E/M motorized stage, which was used for focusing. For illumination, we used red LEDs (TOPLED® - Enhanced Optical Power LED (HOP 2000)), a customized arranged in a 2x2 square array with a spacing such that the array covered a 15x15mm^2 area. To collect and diffuse the light, we placed an aspheric condenser lens with diffuser (ACL2520U-DG15-A, Thorlabs) between the sample and LEDs.

The entire microscope (comprising the light source, objective, camera, intervening optical elements, and focusing stage) was mounted on an X-Y motorized linear stage with stepper motors (NEMA23L IP65 Servotronix) driven by a customized motor controller. The range of travel of this stage was 0.5m in the X-direction and 1.0m in the Y-direction, which was sufficient to cover the entire 8x5 array of plates on the sample rack. This system allowed us to generate a fully-tiled image (1.2 Gigapixels) of the fungal compartment composed of a matrix of 10x15 images (5x7mm2 each) with a 20% overlap, at every time point.

3.2 IMAGE PROCESSING

All the code used for image processing is available at https://github.com/Cocopyth/AMFTravellingWave

3.2.1 Image stitching

For each timestep, we stitched together the network images using the ImageJ Stitching plug-in Grid/Collection Stitching Plugin[67]. In this program, stitching is achieved by aligning images by utilizing cross-correlation of the intensity pattern between neighbouring images. To correct for heterogeneities in the illumination, the background of every image is first subtracted using a "rolling ball" algorithm with a ball radius of 15 px[68].

3.2.2 Network extraction

Our network extraction strategy was inspired by methods developed for fungal colonies by Heaton et al. (see [69] for mathematical details). Briefly, we first removed the background using the same rolling ball algorithm as above, then enhanced edges using the bowler hat algorithm[70] and used anisotropic smoothing to maintain connectivity of the network[71]. Edge enhancement algorithms tend to put lower weight on junction points with the risk of losing connectivity. Anisotropic smoothing allowed us to make sure junction points stay connected by expending edges slightly in following along their own local orientation. After these steps, we hysteresis-thresholded the images using the apply_hysteresis_threshold function of the skimage module of Python[59]. The high threshold is set to the minimum between the pixel value 90 and the 99.5th percentile of pixel values. The low percentile is set to be the maximum between a pixel value of 20 and the 93rd percentile of pixel values. We then stitched the resulting thresholded images together using the stitching parameters found during image stitching. The result was thinned out to a one pixel wide skeleton using the ximgproc.thinning routine of the cv2 package of Python [72].

We manually adjusted all the parameters of the extraction (*i.e.* edge enhancement, anisotropic smoothing, thresholding) once for all plates of the same strain. The aim was to maximize the extraction quality with a focus on maintaining connectivity and on minimizing the detection bias between thicker and thinner edges. Pixel level segmentation precision and recall were not considered a good metric to evaluate the quality of network extraction since it overlooks the importance of connectivity. We therefore evaluated the precision and recall of edge detection. An edge was considered to be correctly detected if both of its endpoint nodes were positioned

correctly and were of the right degree. To extract these metrics, we compared the extracted graph to the raw images of a given plate in randomly selected regions and counted manually the number of false-positive and false-negative detections of graph edges. The precision of edge detection (*i.e.* true positives divided by the sum of true positives and false positives) was estimated to be 0.93, while recall (true positives divided by the sum of true positives and false negatives) was estimated to be 0.96.

3.2.3 Network Overlay

Due to differences in the image-stitching parameters and/or imperfections in the repeatability of the imaging robot's relative positioning of the optics on the sample, networks at different timesteps did not always fully overlap. We therefore had to identify the correct translation and rotation to align extracted networks. For the overlay, we used the "Coherent Point Drift" (CPD) algorithm[73], using randomly selected subset of points from the skeleton. Because it relies on the relationship between different points more than on individual "nearest neighbour" routines, the algorithm is good at roughly matching structured shapes even when they are heavily shifted relative to each other and with a fair amount of noise. Using Python/C++ implementation in the cycpd package[74] with a sufficiently high number of random points taken from the skeleton (1% of all points of the skeleton), the computation was tractable, and a first rough overlay was obtained with this routine. The second routine to more finely adjust the translation and rotation used the iterative closest point algorithm (ICP)[75] . Here, we employed a Python/C++ implementation of the package Open3d[76] on the subset of points from the skeleton corresponding to junctions.

3.2.4 Graph generation

Using a custom algorithm based on pixel neighbouring relations in the skeleton, we transformed the pixel skeletons to a graph format where all junctions and apexes become "nodes" of the graph and branches between them become "edges". Pixels of the skeleton that had 0, 1 3 or 4 neighbours were therefore considered "nodes" and two nodes were connected by an "edge" if they were connected by a consecutive series of neighbouring skeleton pixels with no intervening nodes among them.

3.2.5 Node tracking

After transformation to a graph, we employed two postprocessing steps to identify and track every unique node over time and space. First, we removed the small unconnected components not belonging to the fungal colony. We computed a score based on the total length of each connected component. We then removed all those below 1 mm. This was chosen because most of these connected components would reach such a size within a few timepoints if they really corresponded to fungal material.

Second, to ensure consistent node identification at junctions, we introduced steps to confirm degree 3 and degree 4 nodes. Degree 3 nodes correspond to branching or to anastomosis points. Degree 4 nodes were found in the network when two hyphae crossed, or because of a rarely occurring double branching event at a single location. Because the skeleton was only one pixel wide, the degree 4 nodes were sometimes detected as two very close degree 3 nodes.

To solve the problem leading to incorrect node identification, we reassigned any detected pairs of degree 3 nodes that were within 30 pixels of each other as a single degree 4 node. Next, we assumed that nodes of degree 3 or more were static across frames, apart from a small movement (<10px) due to noise of the skeletonization process and imperfect alignment of successive image frames. Therefore, for each node at a given time t we considered the nearest neighbouring node at time $t+1$ as "biologically identical" and assigned them the same label. Sometimes ambiguity would arise because two nodes at time t have the same nearest-neighbour node at time $t+1$. In those cases, we linked the node of time t with that node at time $t+1$ lying nearest to the position of this ambiguous node at time t.

In general, degree 1 nodes are more difficult to track since they are effectively moving from apical extension. For each degree 1 node at time t, the algorithm first identified the closest edge at time $t+1$ and set it as the *current edge*. Then it selected one of the two nodes of that edge based on the direction of tip growth. Direction of tip growth was defined based on the orientation of the edge connecting to that degree 1 node at time t. The selected node was set to be the *current node*. Then the algorithm operated recursively. If the current node was of degree 1, the algorithm stopped and established identity between that node of time $t+1$ and the initial one at time t. Otherwise, it compared the angles between the *current edge* and other edges that connect to the *current node*. The edge that subtended the smallest deflection angle relative to the *current edge* was selected. That angle

was then compared with the angle between the *selected edge* and all other edges joined at the junction. If any of those deflection angles were smaller than the angle between the *current edge* and the *selected edge*, then the algorithm stopped. We established identity between the current node and the node that was of degree 1 at timestep t and that now, at time $t+1$, became of degree 3 or more (*i.e.* the apex anastomosed). Otherwise, the other endpoint node of the *selected edge* became the *current node*, and the *selected edge* became the *current edge*. This algorithm could correctly track hyphal tip when crossing over an already existing hyphal filament. Indeed, this kind of event creates a degree 4 junction where the path of lowest deflection angle followed by the algorithm would continue across the junction. To evaluate performance, we selected randomly growing tips and visually assessed that more than 98% of them were correctly tracked over two timesteps. We found that the main runner hyphae were generally more reliably tracked over several timepoints compared to the small, branched absorbing structures.

3.2.6 Spore extraction

We extracted spore number and biovolume from images using the SimpleBlobDetector function in OpenCV [72]. The function was set up to detect blobs with a minimum area of 500 pixels and a maximum area of 50000 pixels. We set the minimum circularity to 0.4 to ensure that only round or elliptical blobs were detected. Before running the SimpleBlobDetector function, we removed the background of the image to simplify the detection process. We applied this function to each image individually, saving the position and radius (from which volume was computed) of each individual spore. We then tracked individual spores across timepoints by minimizing the distance between center points.

All code related to network and spore extraction and node tracking can be found at: https://github.com/Cocopyth/AMFtrack

3.3 SPATIAL AVERAGING

3.3.1 Definition and use of rings for density mapping

At each timestep, we first separated the full colony graph into its connected component subgraphs (sets of nodes that have at least one path between them through the graph). The number of such connected subgraphs can be greater than one because crossings into the root compartment can occur more than once on a single plate. Such disjoint networks were not the most common occurrence, but also not rare. However, when they did occur, disjoint subgraphs tended to join with one another through anastomosis within a few time steps, to yield a single connected graph.

We then generated polygons (or multiple polygons in the case of multiple subgraphs) connecting the leading apexes of the network graph(s). Mathematically, these polygons correspond to the convex hull of each graph. We then selected a subset of these convex hulls at successive time points to define a set of rings (polygonal annuli) of approximately equal area. In the case of multiple subgraphs, each ring was discontinuous and went around each of the crossing front. Hyphal filament density ρ was calculated by dividing the total length of graph edges lying within each ring by the ring area. We calculated the growing tip density n by dividing the total number of growing tips lying within each ring by the ring area (see below for exact procedure of computing ring area, hyphal length, and growing tip numbers.)

We also tested an alternative scheme in which rings of equal width (rather than equal area) were used to compute density. We confirmed that there were no qualitative differences in results, namely the emergence of the traveling-wave pattern of density dynamics, and no significant quantitative differences in traveling wave parameters (the saturating density ρ_{sat} and wave speed c).

3.3.2 Hyphal length definition

In a given region of interest (ROI), we defined length as the sum of the curvilinear length of all edges of the network graph contained within that region. We computed curvilinear length of an using the list of pixels of the skeleton composing the edge and added the Euclidian distance between successive pixels after down sampling the total list of pixels by taking one out of every ten skeleton pixels. We multiplied the resulting length in pixel units by 1.725um/pixel corresponding to the pixel size projected onto the sample plane at the (2x) magnification of the optical system.

3.3.3 Area definition

We defined the area of a given region by using the area function of the Python shapely module[77]. For the total colony area, we first computed the area of each connected component subgraph using the polygon defined by the convex hull of the set of nodes composing that subgraph. We then summed over all these subgraph areas to obtain the total colony area.

3.4 MICROSCOPIC NETWORK CONNECTIVITY

3.4.1 Connectivity of overlapping hyphae (degree 4 nodes)

We typically detected nodes of the 2D network that corresponded to two hyphae overlapping without tip annihilation during crossing as degree 4 nodes. Such events were not rare. Assessing visually these degree 4 nodes at high magnification revealed that at early time they would usually not correspond to an actual biological connection (i.e., with a cytoplasmic continuity). However, after about a week, we found these junctions fused, either at points of direct contact between existing walls, or via small hyphal side branches that emerged to connect the two overlapping hyphae. Observing a total of 128 positions corresponding to overlapping hyphae at higher (50x,10x) magnification and confirming cytoplasmic continuity by imaging the flows, we estimated that about 34% of all these points could be considered “fused”.

3.4.2 Connectivity of anastomosis and crossing points

To confirm connectivity of anastomosis point detected in 2x magnification, we randomly sampled degree 3 nodes that were suspected to be anastomosis points. Imaging of the flows in higher magnification further confirmed whether or not there was complete cytoplasmic continuity. Out of 18 such suspected anastomosis points, 17 displayed cytoplasmic continuity. This led to the conclusion that when a degree 1 node corresponding to a growing tip, detected at 2x magnification, became a degree 3 node by ceasing its growth at a position occupied by an existing hyphal filament, it corresponds to an actual anastomosis event.

3.5 PHOSPHORUS MEASUREMENT

3.5.1 Agar and root prep

We calculated phosphorus concentrations in fungal and root compartments by first removing cellophane from the fungal agar, and then cutting the agar into equal sized pieces (up to 18 pieces). Each agar cube corresponded to a spatial position either away or close to the root. We cut the root compartment agar into two pieces and carefully separated the root from the root agar. We weighed each agar piece and placed it in a Teflon cylinder, and we placed each root in a kraft envelope. We put the Teflon cylinders and envelopes in the oven at 70°C for two days to dry. After drying, we weighed roots and placed them in a Teflon cylinder.

3.5.2 Digestion

We added 0.5 ml of digestion mixture (HNO3/HCl 4:1) to the Teflon cylinders using a repeating pipette and left the cylinders open for 30 mins to release gases. We then placed the closed cylinders in a destruction oven at 140°C with the temperature limit set to 160°C for 7 hours. We opened the cylinders, added 2 ml of demiwater using a dispenser and transferred the contents of the cylinder to a test tube. We left the test tubes in a fume cupboard for at least one day to release acid fumes and covered them with plastic foil before placing them in the cold room for a week.

Spectrophotometric determination of phosphate content

The phosphate estimation was based on the formation and reduction of phosphomolybdate. Following[78], we pipetted 150ul of the solution obtained after digestion in test tubes and added 4mL of colour reagent. The colour reagent was prepared in a 1L water solution with 13.33mL concentrated H2SO4, 1.14g ammonium

heptamolybdate, 1.00g ascorbic acid and 0.026g of potassium antimony. The test tubes were left for 30 mins for the colour to form. We measured absorbance at 880nm in a spectrophotometer using plastic cuvettes. Because P is known to be bound/adsorbed by soluble aluminium, iron, and manganese at low pH[79], we calculated that $\sim 2\mu g/mL$ of P was inaccessible in root and fungal compartments, which we used as our baseline in **Ext. Data. Fig. 6**.

3.6 INTRARADICAL COLONIZATION QUANTIFICATION

3.6.1 Counting nuclei

We added the concentration (copies/µL) from both FAM and HEX channels (MAT3 and MAT6 loci due to the heterokaryotic status of the A5 strain used) and we used the final value to calculate the total AM fungal nuclear content in the roots using a modified formula from Kokkoris et al.[63].

$$final\ value\ =\ \left(QuantaLife\ value\ \cdot\ \left(\frac{RV}{SQ}\right)\cdot EQ\right)$$

Where the QuantaLife value is the additive value of the copies/µL from the software for the two channels, RV is the volume of the reaction, SQ is sample DNA quantity used in the reaction, and EQ is the quantity of DNA extracted.

3.6.2 Total length of intraradical hyphae

3.6.2.1 Fixation and nuclear staining in intraradical mycelium

To be able to translate the number of nuclei to intraradical mycelium (IRM) length, we used a protocol to fix AM fungal mycelia of *R. irregularis* A5, followed by nuclear staining. This allowed for quantification of average number of nuclei per um of hyphae. As in experiments above, we harvested fungi grown in split plates. We harvested replicates across different time points to allow for variation in the fungal life stage. Before harvest, each Petri plate was stored for at least 2 days at 4°C to slow down cytoplasmic flows and avoid disturbance in the nuclei distribution during fixation. The growing network on top of the cellophane was then fixed and DAPI stained to visualize the nuclei. Specifically, the network was fixed by slowly applying PFA 4% in 1x PBS (100 mM KHP04, pH=7.4) with a syringe pump (Fusion200, ChemYX) at a flow rate of 50.000 μL/hour for 3 mins. The syringe tip was connected to the cellophane with IBIDI silicone tubing (IBIDI ref:10842). Then, the network was incubated for 45 minutes submerged in a thin layer of 4% PFA at RT. After fixation, the PFA was removed by reversing the direction of the syringe pump at the same flow rate. Lastly, the network was DAPI-stained by adding DAPI (5 µg/mL) at a flow rate of 50.000 ul/hour for 3 mins and incubating at room temperature for 10 mins. The DAPI was removed reversing the direction of the syringe pump at the same flow rate. Pieces of cellophane containing network were cut out and placed on glass slides. The pieces were covered by glass square coverslips for imaging.

3.6.2.2 Nuclear visualization

Cellophane cutouts were imaged using an Olympus BX63, a wide-field epifluorescence microscope, equipped with Ultrasonic stage and UPlanSApo 60× 1.35 NA oil-immersion objective (Olympus). Lumencore SOLA FISH light source, a Hamamatsu ORCAFusion sCMOS camera (6.5 µm pixel size, 2304 x 2304 pixels) mounted using U-CMT C-Mount Adapter, and zero-pixel shift filter set: F36-500 DAPI HC Brightline Bandpass Filter. The software used to control the microscope was CellSens Dimension 2.3 Build 18987. Per image position, field of view (fov), tens to hundreds DAPI (exposure time 5 ms) planes, depending on the thickness of the hyphal network, were taken with a spacing of 0.3 µm between each plane. Images were taken with a 60x objective.

3.6.2.3 Image analysis

To obtain calculations for density of nuclei per hyphal length, we performed a segmentation analysis, analysing 8,14 and 15 fields of view (fovs) for replicates 1-3. For the analysis, firstly the nuclei in the 8 fovs of the first replicate were manually annotated in the python image viewer 'Napari'[80]. These 8 images and nuclear masks were

divided into 144 x 144 x number of zplane crops. Crops that contained at least 3 nuclei (202 crops in total) were kept for training in a 3D StarDist model[81]. Default settings for data augmentation and training were used (see Notebook 1_training – AMF.ipynb). Next, this trained model was used to predict the nuclear mask for the images in replicates 2 and 3 (see Notebook 2_prediction – AMF.ipynb). The predicted nuclear masks were manually corrected using Napari. Next, Cell masks of the overall network were created by manually annotating a maximum projected image of the DAPI channel for every field of view. To calculate, the length of the hyphal network these cell masks were skeletonized using the medial_axis function from scikit-image morphology library[59]. These skeleton masks were checked in the Napari-viewer and corrected manually if necessary. Lastly, the nuclear density per area and length were calculated and plotted by dividing the number of nuclei by the length of the skeleton mask per FOV using the notebook "Calculate_Nuclear_Density.ipynb". Knowing the average number of nuclei per um of hyphae allows us to calculate the total IRM using the nuclear count values originated from the ddPCR analysis **Ext. Data. Fig. 14**, **SI Video 8**)

3.6.3 Colonized root imaging

The following describes the methods to obtain Ext. Data. Fig. 14 f and g.

3.6.3.1 Sample information and DAPI staining

A single root fragment was cut using a tweezer and surgical blade from the root side of a ROC organ split plate culture (*Rhizophagus irregularis* strain A5 with *Dacus carota* carrot root). The sample was grown for ~4.6 months at 25 degrees. For DAPI staining and imaging, the sample was placed on a glass slide and mounted with DAPI mounting solution (ProLong Gold antifade reagent, Invitrogen #P36934). The sample was covered with an 18 mm round coverglass. The mountant solution was allowed to polymerize at room temperature overnight in the dark.

3.6.3.2 Imaging description

Root section was imaged using an ZEISS Axio Imager M2 motorized upright wide-field epifluorescence microscope with Z-drive mot. and TFT monitor, equipped with a Scanning stage (130x85 STEP), and EC Plan-Neofluar 20x/0.50 M27 objective, X-cite Xylis XT720L LaserLED Hybrid drive light-source, Axiocam 305 mono R2 camera (3.45 µm pixel size, 2464 x 2056 pixels), and a Zeiss 49 DAPI Filter set (EX G 365, BS FT 395, EM BP 445/50). The software used to control the microscope was ZEN Pro 3.9 (V. 3.9.101.01000, Build id: 3.23.23348.14). For the image, 57 Z-stacks were taken with a spacing of 1 µm, and an exposure time of 15 ms. The image was maximally projected using ImageJ software (Image>Stack>Z project).

3.7 GRAPH METRICS

3.7.1 Betweenness Centrality

Betweenness centrality $BC(e)$ of an edge e of the graph is the sum of the fraction of pairwise shortest paths that pass through it. It can be computed specifying two (possibly overlapping) groups of nodes, "source nodes" S and "target nodes" T, the set of pairs between which defines the ensemble of shortest paths. More specifically, $BC(e) = \sum_{s \neq t} \frac{\sigma_{st}(e)}{\sigma_{st}}$ where σ_{st} is the number of shortest paths between nodes $s \in S$ and $t \in T$ and $\sigma_{st}(e)$ is the number of those paths that pass through the edge e, and the summation is over all nonidentical pairs of nodes between S and T. The resulting quantity $BC(e)$ indicates how 'central' the edge e is, on average, for the transport of resources between nodes in S and T. For mycorrhizal networks, a meaningful choice for source nodes S are those nodes directly proximal to the root compartment, and for target nodes T, those nodes where carbon resources from the roots are consumed for growth. Note that $BC(e)$ is a symmetric measure in the sense that it is invariant under swapping S and T. Thus, their designation as "source" and "target" is arbitrary, and $BC(e)$ values computed for AM fungal networks (**Figs. 4a,5d**) are equally relevant for both carbon transport towards growing tips, and for transport of phosphorous and other nutrients back to the root. We computed

betweenness centrality using the **edge_betweenness_centrality_subset** function of the Python networkx library[82] on the graph formed by the network.

We manually selected the set of source nodes S as those nodes immediately proximal to the root compartment. We then selected as target nodes T all the degree 1 nodes of the network other than those in S. For shortest path calculations, we provided curvilinear length of edges as weights to the program, so that shortest paths were detected based on the physical (rather than topological) distance along the network graph. We then set the parameter "normalized" to "True" which means the final result is normalized by $2/(n_S * n_T)$, where n_S is the number of source nodes and n_T is the number of target nodes. For each plate, we further normalized betweenness centrality by the highest centrality observed in the network. The latter normalization allowed the betweenness centrality of edges belonging to networks of different sizes to be of comparable magnitude. We noted that these normalizations did not affect the trend described in **Figs. 5** and **Ext. Data Fig. 4**.

3.7.2 Global and root efficiency

To quantify the geometric efficiency of the network graph layout for transport, we computed the 'network efficiency' measure originally proposed by Latora & Marchiori[38], which compares the distance d^{sp} along the shortest path between pairs of nodes to the shortest possible distance d^{E} in the physical space embedding the network.

To consider the transport demands for symbiotic trade, we computed geometric efficiency for two contrasting contexts: (i) transport between arbitrary pairs of nodes within the network, and (ii) transport between network nodes and the plant host. In both cases, the relevant nodes for considering transport are the terminal nodes T, which we define as the set of all nodes corresponding to growing tips (at which nutrients are consumed to build the network) and attachment sites of BAS (through which absorbed nutrients flow into the network), as well as the root nodes R, which we define as the set of all nodes corresponding to sites of crossing from the root compartment (through which C flows into the network). Because only the fungal compartment is visible with our optical system, we define a node connecting both compartments as any node in a 5mm distance near the physical barrier in the plate (see Biological Material and Plate Preparation section).

For transport between arbitrary locations of the network, we average the ratio of distances, d^{E} and d^{sp},over all pairs of nodes in the joint set $P = T \cup R$ to obtain the *global efficiency*[83], defined as $E_{\mathrm{g}} \equiv \sum_{\{i,j\}\in P, i\neq j} d_{ij}^{E} / d_{ij}^{sp}$ where the sums run over all $N_P(N_P - 1)/2$ node pairs with N_P is the number of nodes in P. For transport between the root and the terminal nodes, we average over all root-terminal node pairs to obtain the *root efficiency*, defined as $E_{\mathrm{r}} \equiv \sum_{i\in T, j\in R} d_{ij}^{E} / d_{ij}^{sp}$, where the sums run over all $N_T \times N_R$ node pairs with N_T and N_R the number of nodes in T and R, respectively.

Both efficiency measures E_{g} and E_{r} are effectively normalized by the set of shortest possible (i.e., Euclidian) distances such that $0 < \{E_{\mathrm{g}}, E_{\mathrm{r}}\} \leq 1$. Intuitively, this normalization quantifies how close the shortest paths through the network are, on average, to the shortest possible paths (*i.e.* Euclidian distances) through the physical space in which the network is embedded.

Biologically, simply maximizing the efficiencies is unlikely to be the best strategy because of associated costs. As an extreme example, a totally connected network with $N(N-1)/2$ straight edges between all N of its nodes will maximize both efficiency measures ($\{E_{\mathrm{g}}, E_{\mathrm{r}}\} = 1$), but the large number of edges means the material cost of building the network will also be very high. We therefore further normalized these computed efficiency measures using those for two ideal networks (explained also in the **Main Text**) that represent limiting cases of efficiency priorities, the "Minimum spanning tree (MST) and "Delaunay triangulation" (DT), which correspond to graphs with the minimal and maximal limits, respectively, for both efficiency and cost given a fixed set of nodes[4,38,42,69,84]. We therefore defined (analogous to [42]) the '*relative efficiencies*' as $\hat{E}_g \equiv (E_{\mathrm{g}} - E_{\mathrm{g}}^{\mathrm{MST}})/(E_{\mathrm{g}}^{\mathrm{DT}} - E_{\mathrm{g}}^{\mathrm{MST}})$ and $\hat{E}_r \equiv (E_{\mathrm{r}} - E_{\mathrm{r}}^{\mathrm{MST}})/(E_{\mathrm{r}}^{\mathrm{DT}} - E_{\mathrm{r}}^{\mathrm{MST}})$, where $E_{\mathrm{g}}^{\mathrm{MST}}$ and $E_{\mathrm{g}}^{\mathrm{DT}}$ are the global efficiencies for MST and DT graphs, respectively, and, $E_{\mathrm{r}}^{\mathrm{MST}}$ and $E_{\mathrm{r}}^{\mathrm{DT}}$ are the root efficiencies for MST and DT graphs, respectively. This normalization results in efficiencies between zero and unity ($0 \leq \hat{E}_{g,r} \leq 1$), with $\hat{E}_{g,r} = 0$ corresponding to the efficiency of an MST network for the measured set of nodes, and $\hat{E}_{g,r} = 1$ corresponding to that of a DT network for the same set of nodes.

3.8 TRANSPORT IMAGING

3.8.1 Imaging system

We built a customized microscope system to acquire high-magnification videos of cytoplasmic flows inside the mycorrhizal hyphae, with the imaging-path optical system identical to that used for low-magnification network imaging (objective, 200mm tube lens and a Basler acA4112-30um CMOS camera, mounted on a Thorlabs KMTS25E/M motorized stage). However, we used a different objective lens (100X Nikon CFI60 TU Plan Epi ELWD). A 1 W red fiber optic LED light source (Product ID: 4165, Adafruit Industries) for illumination through an LED reflector assembled with a Fresnel lens to collect and diffuse the light before the beam reached the sample. The sample stage was customized X-Y motorized linear stage, with stepper motors (NEMA23 IP20, Servotronix) driven by an Arduino Uno Rev3 micro-controller. With this setup, every video could be related to a specific coordinate in the fungal network.

For transport imaging, we mounted the sample plate on the X-Y motorized stage, which allowed accurate positioning at specific, recorded, coordinates of the network. These recorded coordinates of the movies within the network could then be used to locate the specific nodes and edges within network graph (see below) for correlative analysis between flow velocities and the geometric / topological observables of the network (**Figs. 5e-f**). At each position with recorded coordinates, we acquired high-resolution (100x magnification) videos at 20-25fps for 30 seconds, with at least 3 repetitions per position. We removed the lid of the sample plate at the time of imaging to maximize image quality.

3.8.2 Spatial mapping of videos in the network

We mapped the spatial position of each flow movie to the skeletonized network to enable analyses of flow velocities as a function of space, as seen in **Figs. 5e-f**. We recorded the acquisition X-Y coordinate within the sample plate for each high-magnification flow movie (using a 100X objective, image size of 141 µm x 103 µm at the sample plane). We aligned the full set of these coordinates for each plate with the network skeleton (extracted from the stitched image of the fungal compartment obtained with a 2X objective) leading to a rough overlap. This first alignment was achieved by matching the X-Y coordinates of one movie (such as an easily identifiable tip) within the skeleton. We then manually performed a finer adjustment of the position of each movie within the network by comparing the exact shape of each hypha imaged with the skeleton. The maximum error for alignment was around 100 µm (of the same order of magnitude as the size of the high-magnification field of view).

Fourier filtering of kymographs

In order to distinguish the traces of particles moving in opposite directions, we implemented in python a method from the software KymographClear[85]. We first tile the kymograph together with flipped version of itself. Then the tiled image is Fourier transformed. Pairs of diagonally opposing (upper left, bottom right/upper right, bottom left) quadrants as well as the central line of the image's Fourier spectrum are then set to zero to generate two images in this Fourier space. We then reverse Fourier transform the resulting images and only keep the central tile to obtain the two (backward/forward) kymographs. This method was used to generate the kymograph shown in **Fig. 5a.**

3.8.3 Hyphal radius determination

Hyphae radii were measured manually using Icy. For each "edge" (hypha segment from one intersection to another) we drew two lines across the hypha that would span its whole external diameter, which includes the hyphal cell wall. The raw measurements in pixels were then converted into µm in the data table using a coefficient of 0.0345µm/pixel. The mean difference between pairs of measures is 0.39µm without removing outliers.

4 SUPPLEMENTARY DISCUSSION

4.1 DIMENSIONALITY (2-D VS 3-D) AND GEOMETRY OF MYCORRHIZAL GROWTH

Our experimental configuration enabled imaging of the full mycelial network graph by confining growth of the mycorrhizal network to two dimensions (2-D), whereas growth in real-world soil habitats unquestionably occurs in three-dimensional (3-D) space. Yet, basic symmetry considerations suggest that the radial traveling-wave range expansions we observe in this 2-D experiment are relevant also for mycorrhizal growth in 3-D environments. Extraradical growth of mycorrhizal networks radiate outward from the plant root – a one-dimensional object – and hence the biologically relevant spatial context obeys cylindrical symmetry – an assumption made also in other modeling studies that have addressed data from 3-D soil environments[15,86]. Our 2-D measurements thus approximate growth patterns along a cross section of a cylinder surrounding the host root, and therefore can be expected to capture salient features of mycorrhizal growth relevant also in 3-D.

4.2 BRANCHING AND ANNIHILATING RANGE EXPANSION (BARE) WAVE MODEL

4.2.1 General model description

The advancing wavefront of hyphal density we observed in growing mycorrhizal networks (**Fig. 2**) is reminiscent of traveling waves observed in range expansion phenomena in growing colonies of bacteria and other microbes[20,22–24,87,88]. In the case of bacteria, properties of the wave-like migrating front have been explained using variants of the Fisher-Kolmogorov (FK) model of population range expansion[17–19]. The FK model is the canonical framework for modeling the propagation of traveling wave fronts in both biological population dynamics[17,19] and physical pattern formation[18], and is described by just one equation,

$$\partial n/\partial t = D\nabla^2 n + kn(1 - \frac{n}{n_{\text{sat}}}), \qquad (1)$$

describing the dynamics of the local population density $n(\boldsymbol{r}, t)$ at position $\boldsymbol{r}$ in d-dimensional space. Its time rate of change $\partial n/\partial t$ is determined by the sum of two contributions on the right hand side. The first term $D\nabla^2 n$ with diffusion coefficient D and ∇^2 the Laplace operator accounts for diffusive spreading of the population due to underlying random motion of individuals. The second term $kn(1 - \frac{n}{n_{\text{sat}}})$ accounts for logistic growth, characterized by growth rate k and saturation density n_{sat}. Given appropriate initial conditions, this equation has traveling-wave solutions of the form $n(r, t) = n(r')$ where the spatial profile of the density $n(r')$ in a reference frame moving at constant speed c along the r-direction (such that $r' = r - ct$) becomes invariant in time, and the orientation $\hat{r}$ of that r-direction is determined by the initial conditions[18]. Thus, the FK model explains traveling waves that arise when individuals of a growing population execute a random walk in space. In intuitive terms, the macroscopic density wave results from successive advances into virgin territory via random motion of individuals, followed by growth up to a density ceiling, typically due to resource limitation.

However, the canonical FK model cannot be applied directly to fungal networks because it fails to capture an essential feature of branched, filamentous growth. In fungi, the population of growing tips can be considered random walkers that explore space through apical growth and branching. These growing tips can interact not only with one another but also with the hyphal filaments they lay down as they move. Inspired by related works on branching morphogenesis in fungal[25,86] and other[26] networks as well as traveling-wave pattern formation[18], we propose a simple model, the Branching and Annihilating Range Expansion (BARE) wave model, that generalizes the FK framework to capture the traveling-wave dynamics we observe in mycorrhizal networks (**Fig. 3a**).

As in the FK framework, we set up a continuum mean-field model that approximates the growth and movement of the underlying population of proliferating random walkers – here the growing tips – in terms of their spatial density, $n(\boldsymbol{r}, t)$ (with units of number per d-dimensional volume, e.g. [mm^{-d}]). To represent the hyphal filaments laid down by growing tips, we introduce an additional variable $\rho(\boldsymbol{r}, t)$ to represent their spatial density (with units of filament length per d-dimensional volume, e.g. [$\mu\text{m/mm}^d$]).

If individual hyphal tips grow at speed v, each tip deposits a cumulative hyphal length of $v\Delta t$ in its path over a time interval Δt. Now, in a region of size $(v\Delta t)^d$ with a growing tip density n, the number of tips will be $N = n \times (v\Delta t)^d$, and the hyphal density will increase by $\Delta\rho = Nv\Delta t/(v\Delta t)^d$. Dividing through by Δt and taking the limit $\Delta t \to 0$ gives a dynamical equation for the hyphal density within the continuum approximation:

$$\partial\rho/\partial t = vn. \quad (2)$$

Note that the mean-field approximation applied here does not explicitly consider possible variation in growth speed across individual tips of the population, and hence if the growth-speed variance is non-zero (as is the case in our experimental data), v is taken to be the average speed of growing tips.

The calculation for growing tip density requires considerations of, along with the creation and annihilation of tips, the directional movement of tips into and out of every point in space. The latter is accounted for by their net spatial flux $\boldsymbol{J}(n)$ (a vector pointing in the direction of net movement, with units of number per time per area in d-dimensional space, e.g. [$\text{mm}^{-(d-1)}\text{s}^{-1}$]). At every point in space, new tips are created due to branching at a rate $b(n,\rho)$ (tip creation events per unit time per unit volume), existing tips are annihilated due to anastomosis at a rate $a(n,\rho)$ (tip annihilation events per unit time per unit volume) and can increase or decrease depending on the divergence of the spatial flux $\boldsymbol{J}(n)$ (increase if $\nabla \cdot \boldsymbol{J}(n) < 0$; decrease if $\nabla \cdot \boldsymbol{J}(n) > 0$). Combining these elements, we obtain the dynamical equation for tip density:

$$\partial n/\partial t = b(n,\rho) - a(n,\rho) - \nabla \cdot \boldsymbol{J}(n). \quad (3)$$

The joint system described by Eqs. 2 and 3 describe the coupled dynamics of growing tips and hyphal filaments. With appropriate choices for $a(n,\rho)$ and $b(n,\rho)$, which are explained in the following section 4.2.2 these equations demonstrate traveling wave solutions in a similar manner to the original FK model (Eq. 1), as we discuss in further detail in the section 4.2.3.

Note that in Eq. 3 we keep the spatial flux $\boldsymbol{J}(n)$ deliberately generic, to allow not only for diffusive, but also advective contribution to spatial fluxes. Whereas the choice $\boldsymbol{J}(n) = -D\nabla n$ corresponds to fully diffusive flux (as in the FK model) and $\boldsymbol{J}(n) = n\mathbf{v}$ corresponds to purely advective flux (with $\mathbf{v}$ a vector field representing the average local velocity of growing tips). More generally, both diffusion and advection can play a role, *i.e.* $\boldsymbol{J}(n) = -D\nabla n + n\mathbf{v}$. The detailed choice of $\boldsymbol{J}(n)$ does not affect whether the model yields traveling wave solutions or not. The specific choice of $\boldsymbol{J}(n)$ does however determine whether and how each microscopic parameter actually contributes to the traveling wave speed, as explained in section 4.3.1.

4.2.2 Expressions for $b(n,\rho)$ and $a(n,\rho)$

In our experiments on AM fungi, we found that new tips always emerge in the vicinity of another growing tip. This means the rate of branching events is not directly dependent on the hyphal filament density but rather on the tip density, *i.e.* $b(n,\rho) = b(n)$. Assuming the simplest case of linear dependence, we set $b(n) = \alpha n$, with α the branching rate per unit time.

For the anastomosis rate $a(n,\rho)$, it is natural to assume a dependence on both densities n and ρ because such fusion events must depend on the rate of collision between tips and hyphal filaments. For tips growing at speed v, assuming the orientation distribution of hyphae is uniform, the probability of colliding with an existing filament between t and $t + dt$ is $\rho v dt$. Assuming every time a tip meets an existing filament, it has a probability p_f of fusing to yield an anastomosis event, the probability for a tip to undergo anastomosis between t and $t + dt$ is $p_f \rho v dt$. Then in a region of tip density n the density of anastomosis events occurring between t and $t + dt$ can be writtein $a(n,\rho)dt = np_f\rho v dt$. Dividing through by dt and defining the rate parameter $\beta \equiv p_f v$, we obtain $a(n,\rho) = \beta n\rho$.

We note that this simple form for $a(n,\rho)$ proportional to both n and ρ is useful for gaining analytical insight (see 4.2.3, below) and for approximate agreement with experimental data (**Fig. 3b**), but its derivation above depends on the assumption of a uniform orientation distribution, which is not fully realistic. In particular, near the expanding front where hyphal densities are low, the relative angles between hyphae and growing tips are not fully randomized because the angular distribution of new branches is not uniform (**Extended Data Fig. 11b**) and most tips have undergone only one or two branching events from the radially expanding runner hyphae. Relaxing the assumption of uniform hyphal orientation in full generality would require a more detailed model that explicitly represents filament orientation as an additional dimension of the densities[89]. For our purposes of understanding the observed traveling wave phenotypes, however, we show below that choosing a slightly

modified form for $a(n,\rho)$ such as $a(n,\rho) = \beta n\rho^2$ (which phenomenologically accounts for the observation that orientations are not fully randomized in regions of low filament density ρ) can yield substantially improved agreement with data (see 4.3.3, below).

While the derivation of these terms is subject to assumptions, the section 4.2.4 details the evidence within our experimental data that support these specific choices for $a(n,\rho)$ and $b(n,\rho)$.

4.2.3 Travelling wave solutions of the BARE wave model yield "pulled waves"

Traveling waves are defined by a density profile that is invariant in a moving coordinate. In our experiment, the density was computed along a radial coordinate r, so we seek solutions that give asymptotically time-invariant density profiles $n(r,t) = n(r')$ and $\rho(r,t) = \rho(r')$ in a transformed radial coordinate $r' = r - ct$ where c is the speed of the traveling wavefront. With this transformation, the dependence of n and ρ on time is removed, and Eqs. 2 and 3 become:

$$-c\frac{\partial\rho}{\partial r} = vn \quad (4)$$
$$-c\frac{\partial n}{\partial r} = b(n,\rho) - a(n,\rho) - \nabla\cdot\boldsymbol{J}(n), \quad (5)$$

where we have renamed the transformed spatial coordinate back to r (i.e. $r' \to r$) for notational simplicity. Solutions of Eqs. 4 and 5 are thus traveling-wave solutions, and as with the FK model (Eq. 1), the conditions for existence for such solutions are not very restrictive. In particular, as also noted above, traveling wave solutions can be found regardless of the specific choice for $\boldsymbol{J}(n)$, from fully advective, or advective-diffusive, to fully diffusive with constant wave speed c and saturating hyphal filament density ρ_{sat} (see **Extended Data Fig. 12** for example simulations demonstrating traveling waves for each of these three choices). Indeed, as long as certain conditions are met for the branching and anastomosis terms and $b(n,\rho)$ and $a(n,\rho)$, the only requirement on $\boldsymbol{J}(n)$ is that it is non-zero. Perhaps not surprisingly, however, the specifics of $\boldsymbol{J}(n)$ do directly affect the wave speed c, a point we discuss in detail within section 4.3.1, below.

One of the simplest choices of $b(n,\rho)$ and $a(n,\rho)$ that can yield traveling wave solutions is the proportional forms $b(n,\rho) = b(n) = \alpha n$ and $a(n,\rho) = \beta n\rho$, respectively [18,25,26]. This choice effectively reduces the dynamics of tip birth and annihilation in Eqs. 3 and 5 to a form of coupled logistic growth, $b(n,\rho) - a(n,\rho) = \alpha n(1 - 2\rho/\rho_{\text{sat}})$, where the negative feedback on growth comes not directly through n but rather through ρ, whose growth in turn is determined by n. Thus, the tip density n increases exponentially at the branching rate α when the local hyphal filament density is low ($\rho \ll \rho_{\text{sat}}/2$), but its value peaks at intermediate filament density (where $\rho = \rho_{\text{sat}}/2$), and finally decays again to zero as the filament density approaches its saturation value ($\rho \to \rho_{\text{sat}}$). The saturation towards ρ_{sat} is independent of the choice of flux term $J(n)$. The value of ρ_{sat} can be obtained by substituting the tip density in Eq. (5) by its expression stated in Eq. (4) and integrating, which yields $\rho_{\text{sat}} = 2\alpha/\beta$.

A Well-known property of the FK model (Eq. 1) is that its traveling-wave solutions are examples of "pulled waves" [18], meaning that the speed c of the advancing wavefront is determined solely by the local dynamics at the wave front, and not by processes behind the front. Mathematically, sufficient conditions for obtaining a pulled wavefront in FK-type models is that the terms accounting for growth and/or annihilation allows growth to proceed exponentially near zero density (mathematically speaking, corresponding to a linear instability) but eventually terminates at a finite saturation density.

The logistic growth expression in the FK model clearly satisfies this condition, as do the above choices for $b(n,\rho)$ and $a(n,\rho)$ in our model (Eqs. 2-3). Thus, traveling wave solutions for our model with the above choice for $b(n,\rho)$ and $a(n,\rho)$ also yield pulled-wave solutions.

The speed of a pulled wave is determined by dynamics at the front[18], which in turn requires some finite outward flux (i.e. $J(n) > 0$, where $J(n) = \boldsymbol{J}(n)\cdot\hat{\boldsymbol{r}}$ is the flux in the radial r-direction indicated by unit vector $\hat{r}$) to continue to advance. Therefore, if the population of growing tips are heterogeneous with respect to their outward growth velocities, the model predicts that the subpopulation with the fastest outward (radial) velocity will dominate over others and determine the wave speed. Because of exponential growth, even if this subpopulation of fastest hyphae are small, their effects on densification are amplified exponentially through branching. In this way, therefore, they can pull the entire density wave even if the bulk of the tip population propagate slower. Variation in outward radiating speed can arise not only due to variation in the speed of growth (which is substantial, as seen in **Fig.3c**), but also due to variation in the orientation of growth. Because the tip population

effectively executes a random walk-through successive branching events, there is always a small subpopulation of tips growing in the radially outward direction of wave propagation. In general, the wave speed will be dictated by the speed of those leading "puller tips" lying at the growing front and with the fastest outward growth speeds.

In summary, our model (Eq. 2-3) yields traveling wave solutions with pulled wavefronts. Whereas the saturation density is determined by the balance of branching and anastomosis terms $b(n, \rho)$ and $a(n, \rho)$, the wave speed is determined primarily by the flux term $\boldsymbol{J}(n)$.

4.2.4 Evidence from experiments and parameter fitting

We tested these ideas with our microscopic tracking data. The tracking data allowed us to identify all branching and anastomosis events during network growth since the network grew on a plane. We estimated the average rates of change in their spatial density $b(n)$ and $a(n, \rho)$ at a given position as the wave passes by computing the temporal density profile of these events through the same spatial averaging and time translation scheme used to obtain the temporal profiles of n and ρ (see **Fig. 2b, right**). By overlaying the temporal profiles of $b(n)$ and $a(n, \rho)$ with those of n and $n\rho$ respectively, we found good agreement in their shape up to linear scaling factors α (=0.04 h^{-1}) and β(=23 µm/h) respectively (**Fig. 3b**).

Consistent with traveling-wave solutions of the model being "pulled waves" (see section 4.2.3, above), the observed wave speed (**Fig. 2d Inset**, $c \approx 280$ um/h) closely matched that of the subpopulation of tips at the advancing front (**Fig. 3c**, cyan, $\langle v_{\mathrm{p}} \rangle \approx 280$ um/h), which was in turn faster than the average speed of the remainder of the growing tip population (**Fig. 3c**, pink; $\langle v \rangle \approx 240$ um/h). Together with the proportionality between the branching and anastomosis event density profiles and of n and $n\rho$ respectively, noted above, these data provide support for our model with coupled logistic growth (specifically, the choice $b(n) = \alpha n$ and $a(n, \rho) = \beta n \rho$) as a basis for an AM fungal traveling wave.

4.2.5 BARE wave model with P absorption

General framework

To model the absorption of phosphorous (P) by the traveling AM fungal wave, we need to consider both the absorptive flux of P through the hyphal membrane and diffusion of P through the medium. Following the methods developed in ref. [15], at any point in time and space, the amount of phosphorous (P) acquired by the colony is a function of the local P concentration and of the hyphal surface area. Approximating hyphal filaments as cylinders of radius r, when the local filament length density is ρ, the density of surface area density is $s = 2\pi r \rho$. The flux φ_P of P into the hyphae is assumed proportional to s but a saturating function of P-concentration (as in an enzymatic reaction), such that $\varphi_P = V_{max} s \frac{[P]}{[P]+K_m}$, with K_m, the Michaelis-Menten constant for absorption. The dynamics of P-concentration in the medium is then determined by the balance of this depleting flux $-\varphi_P$, which depletes P from the medium, and the diffusive flux $D_P \nabla^2[P]$, yielding the following partial differential equation (PDE),:

$$\frac{\partial [P]}{\partial t} = D_P \nabla^2 [P] - \varphi_P, \qquad (6)$$

where D_P is the diffusion coefficient of P in the medium.

Soil specific P dynamics:

P dynamics in agar differ from what can be observed in a real soil. P in soil is in most case reversely bound to soil particles and only a small fraction is in solution. The equilibrium between the liquid fraction concentration C_L and the solid fraction C_S concentration can be represented with the following relation: $\frac{dC_S}{dC_L} = b_p$, where $b_p \approx 200$ represents the phosphorous buffer power of the soil [15]. Within the regime far from saturation of the solid fraction where b_p is a constant, this simplifies to $C_S = b_p C_L$.

The above equations can therefore be adapted to account for this buffering effect. First, the P concentration experienced by transporters at the hyphal surface is C_L and the equation. The expression for ϕ_P is therefore

adapted to be:

$$\varphi_P = \frac{sV_{\max}[P]/b_p}{[P]/b_p + K_m}$$

where $[P] = C_S$ represents the total P bound on soil particles. Then neglecting the diffusion of P on solid surfaces, one can rewrite:

$$\frac{\partial[P]}{\partial t} = D_P \nabla^2 \left(\frac{[P]}{b_p}\right) - \varphi_P$$

This means the diffusion is effectively slowed down by a factor $b_p \approx 200$ which corresponds to the general consensus for the movement of adsorbed species and specifically Phosphorous in soils[90,91].

Modelling parameters

For **Fig. 3e** and **Ext. Data Fig. 5b,c**, we numerically integrated the set of three PDEs comprising the traveling wave model Eqs. (2-3) together with the P absorption model Eq. (6). We chose $\boldsymbol{J}(n) = -nv_d\hat{\boldsymbol{r}} + D\nabla n$ and parameters α =0.039 h^{-1}, $\beta = 22\ um/$h and D = 0.0008 mm^2/h so that $v_d \approx v_{wave}$ and we could more easily vary wave speed.

P absorption parameters were from refs. [15,92] for AM fungi: $r = 5\mu m$ for the (average) hyphal radius, $V_{max} = 2 \times 10^{-5} \mu mol.cm^{-2}.s^{-1}$ and $K_m = 3 \times 10^{-4} \mu mol/cm^3$ for P uptake, and $D_P = 10^{-5} cm^2 s^{-1}$which is the diffusion coefficient of a small molecule in water. As initial conditions we used a uniform P-concentration $[P]_0 = 0.02 \mu g.mm^{-3}$, which corresponds to intermediate concentration of P in soils. For the spatial flux term in Eq. (2), we chose other equation parameters such that $v_{wave} = 250 \mu m.h^{-1}$.

Initial conditions were $\rho(r,0) = 0, n(r,0) = n_{max} e^{-\lambda(r-r_0)^2}$where $n_{\max} = 0.3 mm^{-3}$, $\lambda = 0.2 mm^{-2}$, $r_0 = 7mm$ and $[P](r,0) = [P]_0 \left(1 - e^{-\lambda_2\left(r - r_0^{(P)}\right)}\right)$ where $\lambda_2 = 0.1 mm^{-1}$ and $r_0^{(P)} = 1mm$. The later profile for P concentration was chosen to reflect the existence of a P depletion zone around the plant root.

2-D and 3-D embedding

In the case of modelling of P absorption as shown in **Fig. 3e** and **Ext. Data Fig. 5b,c**, our goal was to represent the consequences in real soil of the observed colony propagation dynamics. We therefore used 3-D concentration of P and 3-D embedding corresponding to cylindrical coordinates with translational symmetry for the tip and hyphal densities along the cylinder axis. The only consequence of this change is the different units in which those densities were expressed, which allowed a direct connection to volumetric P absorption. In particular, this choice did not change the overall dynamics. For **Fig. 3e** and **Ext. Data Fig. 5b,c**, we used a buffer parameter $b_p = 239$. For all other figures (**Ext. Data Fig. 12**), we wanted to represent the dynamics in our system with agar and therefore used a 2-D embedding.

Total mass of P absorbed m_P as shown in **Ext. Data Fig. 5b** was calculated using the integral over space and time of φ.

4.2.6 Conclusion

In summary, our analysis combining both microscopic and macroscopic observables provided agreement that AM fungal traveling waves are generated by an underlying branching random walk, in which coupled logistic growth due to branching and anastomosis is pulled by tips of runner hyphae that radiate outwards at a speed v_p

greater than the rest of the tip population, leaving in their wake a hyphal network of constant, saturating density ρ_{sat}.

Notably, saturation density ρ_{sat} in this BARE wave model is determined by the rate parameters α and β for branching and anastomosis, respectively. Therefore, in contrast to typical population waves, such as those of bacterial colonies modeled by the FK model, the BARE wave model suggests the constant density level behind the traveling wavefront of AM fungi is set not by the carrying capacity of the environment, but rather a morphogenetic program of the fungal network itself. In this sense, the AMF traveling wave is *self-regulating*[89] .

It is also worth noting that only a small subpopulation of fast-growing tips is sufficient to "pull" the wave of mycelial network expansion at their growth speed. Because of this, somewhat counter-intuitively, the speed c of traveling-wave range expansion can be substantially greater than that of the typical growing tip $\langle v \rangle$, as we discuss in more detail below in section 4.3.1.

Finally, we note that while the aim of the BARE wave model described here (Eqs. 2-3) and in the Main Text (Eqs. 1-2) is to provide conceptual and analytical insight, it is also interesting to ask whether further refinements of the model can yield deeper mechanistic insights. To that end, we present below in section 4.3, more detailed discussions of quantitative and mechanistic considerations, as well as suggested directions for future modeling efforts.

4.3 FURTHER DISCUSSION OF THE BARE WAVE MODEL, ITS REFINEMENT, AND ALTERNATIVE MODELS

4.3.1 Choice of $\boldsymbol{J}(n)$

One possible choice for $\boldsymbol{J}(n)$ is to consider that growing tips move in a purely persistent manner: $\boldsymbol{J}(n) = n\mathbf{v}$, where $\mathbf{v}(\boldsymbol{r}, t)$ is the local tip growth velocity (a vector field over the spatial coordinate $\boldsymbol{r}$). This form has been proposed in previous studies of fungal colonies[25,86]. Here, $\mathbf{v}$ is essentially a uniform outward-oriented velocity field, $\mathbf{v} = v\hat{\boldsymbol{r}}$, where v is the speed and $\hat{\boldsymbol{r}}$ is the unit vector in the outward radial direction[25]. This choice appears at first sight quite appropriate for modeling AM fungal growth where the movement of individual growing tips are directionally highly persistent with little or no decorrelation of orientation (**Extended Data Fig. 11a**). However, this uniform-velocity approximation for $\boldsymbol{J}(n)$ leads to somewhat unphysical predictions. Although mathematically, the resulting system of Eqs 2-3 can yield travelling wave solutions, the existence conditions for those solutions require $c > v$, meaning the wave speed must exceed the speed of growing tips[25], which is physically implausible. Furthermore, the wave speed is dependent on initial conditions, and obtaining a traveling wave solution requires in addition that the shape of the initial density profile is an exponential distribution, the spatial decay length of which affects the wave speed over an indefinite distance, which is again unphysical (**Extended Data Fig. 12a**). A purely advective $\boldsymbol{J}(n)$ is therefore an unsuitable choice for describing our experimental observations.

Another possible choice for $\boldsymbol{J}(n)$ is as a purely diffusive flux: $\boldsymbol{J}(n) = -D\nabla n$ where D is a diffusion coefficient. For a hyphal network, the microscopic parameters that determine D are those of the temporal autocorrelation functions of growth speed and orientation, as well as the branching angle distribution of hyphal tips. This choice of $\boldsymbol{J}(n)$ leads to the same spatial flux term as in the classical FK model (Eq. 1). Moreover, a recent study of branching morphogenesis in mammalian mammary glands[26] also used this as the spatial flux term in a model very similar to Eqs. 2-3. Such a choice is partially justified in our system as evidenced by the fact that the direction chosen at birth of a new hypha subtends a randomly distributed angle with average $\langle \theta \rangle \approx 70°$ relative to the growth direction of the parent hypha (**Extended Data Fig. 11b**). Traveling-wave solutions with this choice for $\boldsymbol{J}(n)$ yields a wave speed given by $c = 2\sqrt{D\alpha}$ for the coupled system of Eqs. 2-3, equivalent to that for the well-known FK model solution[17–19]. However, to match the wave speed of our system, one must select a value for the parameter D, which, when combined with other parameters directly measured in our system predicts a width for the peak of growing tip density (27mm at half height) that is well beyond what is observed in our system (6mm at half height) (**Fig. 2B**, **Extended Data Fig. 12b**). We therefore conclude that a purely diffusive $\boldsymbol{J}(n)$ also fails to sufficiently describe traveling waves of AM fungi.

Finally, one can further generalize $\boldsymbol{J}(n)$ to combine both advective and diffusive components: $\boldsymbol{J}(n) = nv_d\hat{\boldsymbol{r}} - D\nabla n$. This choice means that there exists a moving reference frame traveling along $\hat{\boldsymbol{r}}$ at a "drift

speed" v_d, within which the movement of tips are correctly described as purely diffusive. Velocity differences across the growing tip population can emerge not only from growth speed variations/fluctuations but also from orientation randomization due to branching. Indeed, an individual tip's velocity can be positive or negative within this moving reference frame, depending on whether its velocity vector $\boldsymbol{v}$ projected along the outward radial direction $\boldsymbol{v} \cdot \hat{\boldsymbol{r}}$ is greater or less than v_d.

The wave speed c for the traveling-wave solution for Eqs. 2-3 with this advective-diffusive flux for $\boldsymbol{J}(n)$ can be solved for analytically[26]. Given that the speeds of pulled waves are determined only by dynamics of the linear instability at the front[18], we introduce an ansatz $n(r) = Ke^{\lambda r}$ describing the leading profile of the growing tip wavefront along the coordinate r along the propagation direction ($r \equiv \boldsymbol{r} \cdot \hat{\boldsymbol{r}}$). Inserting this ansatz for $n(r)$ and $\boldsymbol{J}(n) = nv_d\hat{\boldsymbol{r}} - D\nabla n$ into Eq. 5 and linearizing, one obtains the following eigenvalue equation,

$$D\lambda^2 + (c - v_d)\lambda + \alpha = 0$$

which yields the solution,

$$\lambda = \frac{-(c - v_d) \pm \sqrt{(c - v_d)^2 - 4D\alpha}}{2D}$$

A necessary condition for a stable wavefront is that λ is real and negative. This leads to the conclusion that c must satisfy $c \geq v_d + 2\sqrt{D\alpha}$. Numerical simulations show that the wave speed selected is precisely the minimum: $v_d + 2\sqrt{D\alpha}$ (**Extended Data Fig. 12c**). This means that given a fixed α, different combinations of v_d and D can yield the same wave speed. These parameters can then be adjusted to match the observed width of the tip density peak. Within this framework, the value of v_d can be interpreted to reflect an average velocity of propagation of tips projected onto the wave propagation direction, and hence there is nothing unphysical about a wave speed greater than v_d. In simulations we find (**Extended Data Fig. 12c**) adjusting D to match the experimentally observed wave speed while constraining v_d and α to experimentally determined values yields a peak width for the n density (6mm at half height) close to that of experiments (**Fig. 2b, lower**). This agreement provides confidence in the advective-diffusive $\boldsymbol{J}(n)$ provides a reasonable approximation for tip spatial flux in AM fungal traveling waves.

In addition to yielding good agreement with the macroscopic density dynamics of AM fungal growth, the choice of an advective-diffusive approximation for $\boldsymbol{J}(n)$ can be further motivated by features of the observed microscopic parameters. First, we found that the growth of individual hyphae after birth is highly directionally persistent (leading to an approximate lower bound for the persistence length $\ell_p \gtrsim 10$ cm; **Extended Data Fig. 11a**), thus motivating an advective component to the flux. Second, the orientation changes upon the birth of a new hypha were randomly distributed, with an average angle $\langle\theta\rangle \sim 70°$ relative to the orientation of the parent hypha (**Extended Data Fig. 11b**), motivating the diffusive term. Third, we also noted the broad distribution of growth speeds that hyphae can adopt (**Fig. 3C**), which can contribute further to the diffusive term. However, we emphasize that the advective-diffusive flux $\boldsymbol{J}(n)$ also remains an imperfect approximation. In particular, for the diffusion approximation to hold accurately, one requires a separation of scales between the diffusive dynamics approximated by the flux term $-D\nabla n$ and the underlying random walk. Yet given the extremely long persistence length of individual hyphae ($\ell_p \gtrsim 10$ cm), the effective persistence length of the tip population's branching random walk L_p will be dominated by orientation randomizations upon branching and will be of the order of the inter-branch interval, i.e. $L_p \sim v/\alpha$, which (given our measured values for v and α) is of order ~6 mm, comparable to the width of the growing tip pulses (**Fig. 2b**). Thus, there is no clear separation of scales, and hence any interpretation of fitted parameters D and v_d in terms of microscopic parameters such as the tip growth speeds and branching statistics will require adequate care. Because of these limitations, we discuss in the following section 4.3.2 one additional alternative formulation of a mean-field model that does allow an explicit representation of the important microscopic parameter v_p, which we found (see above, and **Figs. 2-3**) to be the determinant of the propagation speed c of AM fungal waves.

4.3.2 Other models

An alternative formulation to describe explicitly puller and average hyphae consists in adding a third term to the system of equations explicitly describing a puller population of density n_p. This population moves with a purely advective flux $\boldsymbol{J}(n_p) = n_p v_p \hat{\boldsymbol{r}}$ at a higher speed than the rest of the "normal" population that has density n. A tip from the "normal" population can become puller with a rate K_1 and puller can transition to a "normal" state with a rate K_2 and moves via an advective-diffusive flux $\boldsymbol{J}(n) = nv_d\hat{\boldsymbol{r}} - D\nabla n$. These rates can be interpreted to represent multiple underlying phenomena. Firstly, a change of orientation through branching or limited directional persistence of growth that can direct hyphae towards or away from the direction of propagation of the

wave. Secondly, the broadly variable speeds observed across individual tips within the population of hyphae. This leads to a system of three equations:

$$\begin{cases} \frac{\partial n}{\partial t} = \alpha n - K_1 n + K_2 n_\mathrm{p} - \beta n \rho - \nabla \cdot (n v_d \hat{\boldsymbol{r}}) + D \nabla^2 n \\ \frac{\partial \rho}{\partial t} = n v + n_\mathrm{p} v_\mathrm{p} \\ \frac{\partial n_\mathrm{p}}{\partial t} = \alpha n_\mathrm{p} + K_1 n - K_2 n_\mathrm{p} - \nabla \cdot \left(n_\mathrm{p} v_\mathrm{p} \hat{\boldsymbol{r}} \right) \end{cases} \quad (7)$$

Numerical simulations confirm that this model gives rise to a travelling wave with a wave speed very close to (albeit slightly below) that of growth speed v_p of puller tips (**Extended Data Fig. 12d**). Interestingly, the difference between the speed of the wave and the one of puller hyphae is dependent on the values of α, K_1 and K_2. While this formulation still includes a term that depends on the diffusion approximation, it does allow for an explicit representation of the puller hyphae population and their growth speed v_p, which our analysis (see above) and experimental data (**Figs. 2-3**) revealed to be the key microscopic parameter determining the AM fungal wave speed.

As an extension to this approach, one could relax the assumption of two discrete populations and explicitly model the underlying space of possible speeds and orientations as additional dimensions to the continuous space in which population densities are defined, such that the density then becomes an explicit function of its orientation φ and its speed v, *i.e.* $n = n(\boldsymbol{r}, \varphi, v, t)$. Within this higher-dimensional space, branching formulated as jump Markov processes between orientation and speed states[89] could circumvent the diffusion approximation and may be a promising direction for future modeling endeavors.

These two formulations have some degree of equivalence with the advection-diffusion formulation discussed above but they allow to explicitly describe the puller hyphae population which facilitate the interpretation of the terms opening the possibility of observing them microscopically.

4.3.3 Refinement of morphogenetic terms to match the observed saturation density

The saturation value for the filament density ρ_sat predicted by our model ($\frac{2\alpha}{\beta} = 3.5\ \mathrm{mm}^{-1}$), is of the same order of magnitude as that observed in our experiments ($\approx 1\ \mathrm{mm}^{-1}$ for runner hyphae only; see **Fig. 2b,c**) and yet the exact values are unmatched by a few-fold. Although the aim of the BARE wave model presented in the main text is to provide conceptual and analytical insight, it is interesting to ask what mechanistic factors might account for this quantitative discrepancy. In this section, we present an analysis of multiple factors within the morphogenetic terms accounting for branching and anastomosis that could contribute to this discrepancy, to iteratively refine both the model itself, and the data analysis for calibrating model parameters.

Firstly, our extraction of the density-dependent rate coefficient for anastomosis β from tracking data (**Fig. 3b**, lower) could be too simplistic. That analysis detected anastomoses by looking for events in which the collision of a growing tip, a degree 1 node of the network, with a hyphal filament, an edge of the network, produces a new degree 3 node. However, we did also observe numerous examples of anastomoses that form through a more complex series of events that could have been missed by this analysis. Specifically, we found that even when growing tips did not immediately produce anastomosis upon collision and continued growth after crossing with the hypha with which they collide, hence producing a degree 4 node, they would stop growth after a few hundred micrometers beyond the point of crossing. Follow-up observations at higher magnification revealed that hyphal fusion can occur at those crossing points. While such “overshoot anastomosis” events were not detected by our tracking analysis (**Fig. 3b**, lower), it is possible to account for them by introducing a density-dependent stopping rate s, which can be written $s(n, \rho) = \beta_\mathrm{s} n \rho$ where the rate coefficient β_s can be estimated from experimental data through the same linear scaling analysis of density profiles in the ring reference frame (**Fig. 3b**) that we used to extract α and β (see above, and also section 4.5.1 below). This analysis resulted in the value $\beta_\mathrm{s} = 9\mathrm{um/h}$.

Secondly, one can observe that the matching between the linearly density-dependent model for branching rate $b(n) = \alpha n$ and the experimentally observed branching rate b (**Fig. 3b**, upper) is slightly imperfect, with b decaying faster than tip density αn. We therefore asked whether the branching rate could be better described by a generalized form $b(n, \rho)$ that can depend also on ρ. Specifically, expanding the coefficient α to first order in ρ, we can write $b(n, \rho) = \alpha(\rho) n = \alpha n + \alpha' n \rho$. Thus matching with the the experimentally observed branching rate b requires $b - b(n, \rho) = b - \alpha n - \alpha' n \rho = 0$, or equivalently to finding a value of α' that balances the

residual so that $\alpha n - b(n) = -\alpha' n\rho$. This procedure gives $\alpha' \approx -7\text{um/h}$. A negative value of α' means branching is attenuated in regions of higher filament density ρ.

We can therefore generalize the branching and annihilation rates respectively as $b(n, \rho) = \alpha n + \alpha' n\rho$ and $a(n, \rho) = \beta n\rho + s(n, \rho) = (\beta + \beta_s)n\rho$ in the BARE wave model (Eqs. 4-5). This leads to a solution for the saturation density $\rho_{sat} = 2\alpha/(\beta + \beta_s - \alpha') = 2\ \text{mm}^{-1}$, which is closer to the experimentally observed value but still off by ~2-fold.

Thirdly, it is also noticeable from **Fig. 3b** (lower) that the front of the density product $n\rho$ arrives slightly ahead of that of anastomosis-event density. This discrepancy could arise, for example, from geometric effects in 2-D, because the new tips generated just behind puller tips at the growing front will tend to be oriented outward into virgin territory (because the average branching angle is < 90 degrees; **Extended Data Fig. 11b**), and hence have no chance of colliding with any existing hyphae to produce anastomoses. Such effects can be modeled phenomenologically by generalizing the coefficient β to allow it to depend explicitly on the filament density ρ, i.e. $\beta = \beta(\rho)$. Given that filament density ρ is always lowest at those front regions where we observe a relative paucity of anastomosis events, $\beta(\rho)$ should be an increasing function of ρ. Taking the simplest possible linear form $\beta(\rho) = \beta'\rho$, where β' is a positive constant (with units of length squared per unit time, e.g. [mm^2/h]), we obtain an expression for the anastomosis rate that depends quadratically, rather than linearly on ρ, as $a(n, \rho) = \beta(\rho)n\rho = \beta' n\rho^2$. Similarly, the rate coefficient $\alpha'$of the $n\rho$-dependent term of the branching function may depend on ρ, giving $b(n, \rho) = \alpha n + \alpha'(\rho)n\rho = \alpha n + \alpha'' n\rho^2$. These modifications do not qualitatively alter the travelling wave behavior – the resulting equations still satisfy the conditions of a pulled wave – but does lead to a different expression for the saturation density on the branching and tip annihilation rates, $\rho_{sat} = \sqrt{3\alpha/(\beta' - \alpha'')}$ (obtained through integrating Eqs. 4 and 5 as explained above, under 4.2.3).

Combining all of the above, we considered the possibility that the density-dependent stopping and branching coefficients discussed above (β_s and α', respectively) can similarly depend on ρ, *i.e.* $\beta_s = \beta_s(\rho) = \beta'_s\rho$ and $\alpha' = \alpha'(\rho) = \alpha''\rho$, respectively. Then, all of three of these density-dependent contributions (anastomosis, stopping, and branching attenuation) became proportional to $n\rho^2$ instead of $n\rho$, so that the combined branching and annihilation terms could be written $b(n, \rho) - a(n, \rho) = \alpha n + \alpha'' n\rho^2 - (\beta' + \beta'_s)n\rho^2 = \alpha n - \gamma n\rho^2$, where we have defined $\gamma \equiv \beta' + \beta'_s - \alpha''$. We found that experimental data for the anastomosis rate, stopping rate, and branching attenuation were very well matched by proportional fits to $n\rho^2$ (**Extended Data Fig. 12e-g**), with the constants of proportionality yielding estimates for β', β'_s, and α'', respectively. Using those estimates leads to $\gamma \approx 0.075 mm^2h^{-1}$ yields for the saturating density model solution $\rho_{sat} = \sqrt{3\alpha/\gamma} = 1.3\ \text{mm}^{-1}$, which is in very close agreement with the measured value ($\approx 1\ \text{mm}^{-1}$). We also note that this modified model with $n\rho^2$-dependence robustly yields traveling wave solutions (**Extended Data Fig. 12h-i**) with almost the same wave speed c as that of a model that uses instead the original rate expressions $b(n) = \alpha n$ and $a(n, \rho) = \beta n\rho^2$ but otherwise identical parameters (**Extended Data Fig. 12c**).

In summary, we have demonstrated how morphogenetic terms of the BARE wave model of AM fungi traveling waves presented in the **Main Text** can be iteratively refined to achieve better quantitative agreement with the observed data. Although full quantitative explanation of the data would likely require a more detailed model that explicitly accounts for the variability in microscopic variables such as hyphal orientations and growth speeds, we have shown that many of the quantitative observables within the experimental data can be captured by slightly generalizing the simplest mean-field approximations within the BARE wave model of Eqs. 2-3. Overall, we found a better agreement with experimental data (with the same number of parameters), when the density-dependent rates of anastomosis, stopping (a proxy for overshoot anastomosis), and branching attenuation were all assumed proportional to $n\rho^2$, rather than $n\rho$. It is interesting to ask what mechanism might account for this quadratic dependence on the hyphal filament density ρ. As noted above, for the anastomosis rate (and by extension the related rate of stopping), the correlation between density and the degree of angular randomization must be a contributing factor. However, for the attenuation of the branching rate, it is not obvious how the growth orientation could play a significant role. It would seem more likely that effective dependence of the branching propensity depends on some sensing of the local hyphal density, perhaps through some chemical cue that is either secreted or depleted by hyphal filaments. Exploring these possibilities through further experimental and modeling efforts would be a promising line of future inquiries.

4.3.4 Estimating carbon cost

In order to estimate carbon cost of the colony in **Fig. 4e** we first estimated the total carbon in each edge linking node $\boldsymbol{i}$ and node **j**. To estimate its biovolume, we approximated it to be a cylinder of radius $r = 5\mu m$ and therefore defined its volume $\boldsymbol{V_{i,j}}$ to be $\boldsymbol{V_{i,j} = \pi r^2 L_{i,j}}$ where $\boldsymbol{L_{i,j}}$ is the length of the edge. To then estimate the total carbon in each edge, we assumed fixed ratios true for most biological material. The first one is the ratio of dry mass to wet mass $\boldsymbol{p_{dry}}$ which is generally estimated to be 21% for fungi[93]. The second one is the ratio of carbon in dry mass $\boldsymbol{p_{carbon}}$ that we set to 50%[7]. The third element is the cell density $\boldsymbol{d_{cell}}$ which is generally estimated to be around 1.1 g/cm^3 [93]. Such ratio could theoretically be expected to be different for AMF. They could also differ between edges and within the phylogeny. But these "living cells" averages are a good first-order approximation and don't affect the general conclusion discussed within the scope of this paper.

The mass of carbon contained in the edge is therefore $\boldsymbol{m_{i,j}^C = V_{i,j} d_{cell} p_{dry} p_{carbon}}$

Carbon cost was finally estimated to be $\boldsymbol{\xi_{ij} = \frac{m_{i,j}^C}{CUE} = \gamma L_{i,j}}$ where CUE is the carbon use efficiency i.e. the proportion of carbon incorporated in biomass and not respired of all the carbon used. We used an average CUE for soil microorganisms of 50%[94] as this does not affect the general trend shown in that figure. We therefore obtain

$$\boldsymbol{\xi_{ij} = \gamma L_{ij}}$$

Where $\boldsymbol{\gamma = \frac{\pi r^2 d_{cell} p_{dry} p_{carbon}}{CUE}}$.

We then could estimate the network wide carbon cost of the network by summing over all the edges: $\boldsymbol{C = \sum \xi_{ij}}$. For 1 m of colony length, this typically means the typical carbon mass of hyphae is approximately $3\mu g$ and the total carbon used to grow this biovolume is $\sim 6\mu g$.

We then normalized this network-wide carbon cost normalized with respect to its counterparts for the limiting networks $\hat{C} = (C - C_{MST})/(C_{DT} - C_{MST})$, where C_{DT} is the cost for Delaunay triangulation and C_{MST} is the cost of the minimum-spanning tree.

To estimate carbon cost of phosphorus absorbed in **Ext. Data Fig. 5c** we considered $\boldsymbol{L}$ to be the integral over space of hyphal density. Because of the 3-D embedding and cylindrical symmetry, it therefore represents hyphal length per root length. Total mass of P and C are then given per unit length. Carbon cost of Phosphorus was finally estimated to be $\pi = \frac{\boldsymbol{m_C}}{\boldsymbol{m_P}} \times \frac{\boldsymbol{1}}{\boldsymbol{CUE}}$ with $\boldsymbol{m_P}$ the mass of P as defined in **section 4.2.5**.

4.4 NUMERICAL SIMULATIONS OF TRAVELING-WAVE MODELS

The system of partial differential equations (PDEs) for each model described in **Supplementary Text** were numerically integrated using the Python dolfinx package based on the FEniCS project's computational framework[95] to translate the PDE model into efficient finite element C++ and Python code. A polar coordinate system was used to express explicitly the gradient and Laplacian operators. In that coordinate system and with the expressions $b(n,\rho) = b(n) = \alpha n$, $a(n,\rho) = \beta n\rho$ and $J(n) = n v_d \hat{r} - D\nabla n$, equation (2) and (3) become:

$$\partial\rho/\partial t = vn \tag{8}$$

$$\partial n/\partial t = \alpha n - \beta n\rho - \frac{1}{r}\frac{\partial}{\partial r}(r n v_d) + D\frac{1}{r}\frac{\partial}{\partial r}\left(r\frac{\partial n}{\partial r}\right) \tag{9}$$

Time was discretized using a first order upwind scheme to avoid instabilities due to the advection term. The equations were reformulated in their weak form with Dirichlet boundary conditions by multiplying (7) and (8) by two test functions and integrating second-order derivative by parts.
Space was divided in a mesh of 200 cells and time was divided in 900 elements.

We set initial conditions for the tip density to be a gaussian whose height and width was manually adapted to accelerate the convergence towards the travelling wave solution. In the case of a purely advective flux, we used exponentially decaying initial conditions as described in the caption of **Extended Data Fig. 12**. The solve function of the dolfinx package was used to integrate the equations between each timestep. Notebooks to reproduce these numerical simulations can be found on the dedicated repository at https://github.com/Cocopyth/model_integration.

4.5 LINKING MODELLING AND EXPERIMENTAL PARAMETERS

4.5.1 Parameter extraction from curve proportionality

The parameters in the continuous model represented proportionality factors between various quantities, such as n, ρn, the anastomosis rate, and the branching rate, among others. To obtain accurate estimates of these factors, we computed the average of the temporal dynamics associated with these quantities in the reference frame of successive rings (as shown in **Fig. 3b**, right).

We then extracted fit these average time series within a window width 40 h, centred at the point of their common peak (0h for ρn, anastomosis rate, and all quantities shown in **Extended Data Fig. 12e-g**). This windowing was necessary to avoid the near-zero values of these time series, where noise would otherwise become dominant to corrupt estimates of extracted proportionality factors. We then obtained the proportionality factor by minimizing the mean square fitting error within this window by linear regression.

4.5.2 Stopping rate

To extract stopping rate as shown in **Extended Data Fig. 12f** we first detected stopping events in our tracking analysis, where they were defined as the subset of all events at which a tracked node's gets closer than $70\mu m$ from its final position but stays of degree 1. We recorded for each stopping event its position in space, defined as its final position, and its time, defined as the last timestep where it was further away than $70\mu m$ from its final position. The stopping rate (in units $mm^{-2}hr^{-1}$) within a given ring at time t was computed by dividing the number of stopping events occurring within that ring over a time interval $[t, t + \Delta t]$ by the area of that ring and also by Δt, the time interval between two successive frames.

4.5.3 Total hyphal density

In the versions of the model presented in **Extended Data Fig. 12a-d,h,i**, the modelling assumptions do not represent branched absorbing structures (BAS) that have different dynamics of growth and branching from those of runner hyphae (RH). Parameter extraction must therefore be done considering only tips and hyphal filaments that belong to runner hyphae for parameters to accurately represent the growth dynamics of this subpopulation. This is why in order to extract parameters from **Fig3.b** and **Extended Data Fig. 12e/f/g**, we used RH density for the quantity $\boldsymbol{\rho}$ and RH tip density is used for the quantity $\boldsymbol{n}$.

The total hyphal density as shown in **Fig 2.** can then be extrapolated using the constant density of BAS shown in **Fig. 4b**. Specifically, since BAS represent 30-40% of the total hyphal length, we can multiply the runner hypha density by a factor of 1.4-1.6 to obtain the total hyphal density.

5 SUPPLEMENTARY REFERENCES

67. Preibisch, S., Saalfeld, S. & Tomancak, P. Globally optimal stitching of tiled 3D microscopic image acquisitions. *Bioinformatics* **25**, 1463–1465 (2009).
68. Sternberg. Biomedical Image Processing. *Computer* **16**, 22–34 (1983).
69. Heaton, L. *et al.* Analysis of fungal networks. *Fungal Biol. Rev.* **26**, 12–29 (2012).
70. Sazak, Ç., Nelson, C. J. & Obara, B. The multiscale bowler-hat transform for blood vessel enhancement in retinal images. *Pattern Recognit.* **88**, 739–750 (2019).
71. Long, Y. *et al.* Cellular Heterogeneity in Pressure and Growth Emerges from Tissue Topology and Geometry. *Curr. Biol.* **30**, 1504-1516.e8 (2020).
72. Bradski, G. *The Opencv Library*. *Dr. Dobb's J. Softw. Tools* vol. 25 (2000).
73. Myronenko, A. & Song, X. Point-Set Registration: Coherent Point Drift. *IEEE Trans. Pattern Anal. Mach. Intell.* **32**, 2262–2275 (2010).
74. Anthony. Cython-CPD. (2023).
75. Besl, P. J. & McKay, N. D. A method for registration of 3-D shapes. *IEEE Trans. Pattern Anal. Mach. Intell.* **14**, 239–256 (1992).
76. Zhou, Q.-Y., Park, J. & Koltun, V. Open3D: A Modern Library for 3D Data Processing. Preprint at https://doi.org/10.48550/arXiv.1801.09847 (2018).
77. Gillies, S. *et al.* Shapely. https://doi.org/10.5281/zenodo.5597138 (2023).
78. Murphy, J. & Riley, J. P. A modified single solution method for the determination of phosphate in natural waters. *Anal. Chim. Acta* **27**, 31–36 (1962).
79. Etesami, H., Jeong, B. R. & Glick, B. R. Contribution of Arbuscular Mycorrhizal Fungi, Phosphate–Solubilizing Bacteria, and Silicon to P Uptake by Plant. *Front. Plant Sci.* **12**, (2021).
80. Ahlers, J. *et al.* napari: a multi-dimensional image viewer for Python. Zenodo https://doi.org/10.5281/zenodo.8115575 (2023).
81. Weigert, M., Schmidt, U., Haase, R., Sugawara, K. & Myers, G. Star-convex Polyhedra for 3D Object Detection and Segmentation in Microscopy. in *2020 IEEE Winter Conference on Applications of Computer Vision (WACV)* 3655–3662 (2020). doi:10.1109/WACV45572.2020.9093435.
82. Hagberg, A., Swart, P. J. & Schult, D. A. *Exploring Network Structure, Dynamics, and Function Using NetworkX*. https://www.osti.gov/biblio/960616 (2008).
83. Aldous, D. J. & Shun, J. Connected Spatial Networks over Random Points and a Route-Length Statistic. *Stat. Sci.* **25**, 275–288 (2010).
84. Latora, V. & Marchiori, M. Economic small-world behavior in weighted networks. *Eur. Phys. J. B - Condens. Matter Complex Syst.* **32**, 249–263 (2003).
85. Mangeol, P., Prevo, B. & Peterman, E. J. G. KymographClear and KymographDirect: two tools for the automated quantitative analysis of molecular and cellular dynamics using kymographs. *Mol. Biol. Cell* **27**, 1948–1957 (2016).
86. Schnepf, A., Roose, T. & Schweiger, P. Growth model for arbuscular mycorrhizal fungi. *J. R. Soc. Interface* **5**, 773–784 (2007).
87. Gandhi, S. R., Yurtsev, E. A., Korolev, K. S. & Gore, J. Range expansions transition from pulled to pushed waves as growth becomes more cooperative in an experimental microbial population. *Proc. Natl. Acad. Sci.* **113**, 6922–6927 (2016).
88. Warren, M. R. *et al.* Spatiotemporal establishment of dense bacterial colonies growing on hard agar. *eLife* **8**, e41093 (2019).
89. Edelstein-Keshet, L. & Ermentrout, B. Models for Branching Networks in Two Dimensions. *SIAM J. Appl. Math.* **49**, 1136–1157 (1989).
90. Bieleski, R. L. Phosphate Pools, Phosphate Transport, and Phosphate Availability. *Annu. Rev. Plant Biol.* **24**, 225–252 (1973).
91. Darrah, P. R. & Staunton, S. A mathematical model of root uptake of cations incorporating root turnover, distribution within the plant, and recycling of absorbed species. *Eur. J. Soil Sci.* **51**, 643–653 (2000).
92. Schweiger, P. F. & Jakobsen, I. The Role of Mycorrhizas in Plant P Nutrition: Fungal Uptake Kinetics and Genotype Variation. in *Plant Nutrition — Molecular Biology and Genetics* (eds. Gissel-Nielsen, G. & Jensen, A.) 277–289 (Springer Netherlands, Dordrecht, 1999). doi:10.1007/978-94-017-2685-6_31.
93. Bakken, L. R. & Olsen, R. A. Buoyant Densities and Dry-Matter Contents of Microorganisms: Conversion of a Measured Biovolume into Biomass. *Appl. Environ. Microbiol.* **45**, 1188–1195 (1983).
94. Manzoni, S., Taylor, P., Richter, A., Porporato, A. & Ågren, G. I. Environmental and stoichiometric controls on microbial carbon-use efficiency in soils. *New Phytol.* **196**, 79–91 (2012).
95. Scroggs, M. W., Dokken, J. S., Richardson, C. N. & Wells, G. N. Construction of Arbitrary Order Finite Element Degree-of-Freedom Maps on Polygonal and Polyhedral Cell Meshes. *ACM Trans Math Softw* **48**, 18:1-18:23 (2022).
