# Supplementary material for: A travelling-wave strategy for plant–fungal trade
Source: Nature. 2025 Feb 26;639(8053):172–80. doi: 10.1038/s41586-025-08614-x (PMC11882455; doi:10.1038/s41586-025-08614-x)
Supplement: Supplementary file 2 — Reporting Summary [file 41586_2025_8614_MOESM2_ESM.pdf]

nature portfolio

Corresponding author(s): E. Toby Kiers; Howard A. Stone; Thomas S. Shimizu

Last updated by author(s): 25/11/2024

Reporting Summary

Nature Portfolio wishes to improve the reproducibility of the work that we publish. This form provides structure for consistency and transparency in reporting. For further information on Nature Portfolio policies, see our Editorial Policies and the Editorial Policy Checklist.

Statistics

For all statistical analyses, confirm that the following items are present in the figure legend, table legend, main text, or Methods section.

n/a	Confirmed	
☐	☒	The exact sample size (*n*) for each experimental group/condition, given as a discrete number and unit of measurement
☐	☒	A statement on whether measurements were taken from distinct samples or whether the same sample was measured repeatedly
☒	☐	The statistical test(s) used AND whether they are one- or two-sided<br>*Only common tests should be described solely by name; describe more complex techniques in the Methods section.*
☒	☐	A description of all covariates tested
☒	☐	A description of any assumptions or corrections, such as tests of normality and adjustment for multiple comparisons
☐	☒	A full description of the statistical parameters including central tendency (e.g. means) or other basic estimates (e.g. regression coefficient) AND variation (e.g. standard deviation) or associated estimates of uncertainty (e.g. confidence intervals)
☒	☐	For null hypothesis testing, the test statistic (e.g. *F*, *t*, *r*) with confidence intervals, effect sizes, degrees of freedom and *P* value noted<br>*Give P values as exact values whenever suitable.*
☒	☐	For Bayesian analysis, information on the choice of priors and Markov chain Monte Carlo settings
☒	☐	For hierarchical and complex designs, identification of the appropriate level for tests and full reporting of outcomes
☐	☒	Estimates of effect sizes (e.g. Cohen's *d*, Pearson's *r*), indicating how they were calculated

Our web collection on statistics for biologists contains articles on many of the points above.

Software and code

Policy information about availability of computer code

Data collection	The tile images of fungal time-lapses were acquired using a custom software designed by AMOLF engineering staff. The high resolution videos were acquired using Pylon Viewer 5.1.0 (camera software) and motors controlled using Arduino IDE software. The software used to control the microscope for root section colonization imaging was ZEN Pro 3.9 (V. 3.9.101.01000, Build id: 3.23.23348.14). The software used to control the microscope for fungal nuclei imaging was CellSens Dimension 2.3 Build 18987.
Data analysis	The tile images were stitched using Fiji distribution of ImageJ 1.53q and its plug-in 'Stitching Grid/Collection'. The network extraction and node tracking was performed with custom python code. All code used for segmentation, node tracking and post-analysis can be found at the following repository https://github.com/Cocopyth/AMFTravellingWave. Packages used are indicated as dependencies in the / Kymograph were extracted from videos using MATLAB. Speeds were extracted from kymographs using KymoButler (Wolfram Mathematica).

For manuscripts utilizing custom algorithms or software that are central to the research but not yet described in published literature, software must be made available to editors and reviewers. We strongly encourage code deposition in a community repository (e.g. GitHub). See the Nature Portfolio guidelines for submitting code & software for further information.

Data

Policy information about availability of data

All manuscripts must include a data availability statement. This statement should provide the following information, where applicable:

- Accession codes, unique identifiers, or web links for publicly available datasets
- A description of any restrictions on data availability
- For clinical datasets or third party data, please ensure that the statement adheres to our policy

Data availability

Raw data to reproduce the main analysis as well as source data necessary for all main figures are made available together with associated code in the following data repository (https://doi.org/10.6084/M9.FIGSHARE.27889143). Detailed instructions for installation and how to reproduce these results are provided.

Research involving human participants, their data, or biological material

Policy information about studies with human participants or human data. See also policy information about sex, gender (identity/presentation), and sexual orientation and race, ethnicity and racism.

Reporting on sex and gender	N.A.
Reporting on race, ethnicity, or other socially relevant groupings	N.A.
Population characteristics	N.A.
Recruitment	N.A.
Ethics oversight	N.A.

Note that full information on the approval of the study protocol must also be provided in the manuscript.

Field-specific reporting

Please select the one below that is the best fit for your research. If you are not sure, read the appropriate sections before making your selection.

☒ Life sciences ☐ Behavioural & social sciences ☐ Ecological, evolutionary & environmental sciences

For a reference copy of the document with all sections, see nature.com/documents/nr-reporting-summary-flat.pdf

Life sciences study design

All studies must disclose on these points even when the disclosure is negative.

Sample size

For network data, we prepared a number of plates necessary to obtain a minimum of 4 biological replicates per treatment. This number was chosen in order to allow quantitative comparison accross treatment taking into account the variability of biological samples while allowing sampling accross the parameter space within experimental temporal constraints. Based on the difference between hyphal density accross the two closest strains (std = 1000um/mm^2) and the estimated std for hyphal density values accross samples in A5 (~300um/mm^2) we estimated that n=4 replicates are sufficient (2*t(n-1)*std/sqrt(n) = 900). In some case, more plates were lost during data acquisition than initially planned leading to reduced sample size. We explain why the sample size are still sufficient in those specific cases below. Measured quantities such as puller hyphae growth speed and saturation density could vary from sample to sample but the general travelling wave pattern was robustly observed every time the network fully colonized the split plate's fungal compartment (23 independent biological samples across Extended Data Figs. 1,4,5,10 with a minimum of 3 for Extended Data Figs. 10). In all these cases, since the effect was seen in all replicates, we decided that a sample size of 3 was sufficient to demonstrate the consistency of the phenomenology. For effects of carbon/ strain treatments (Ext. Data Figs. 2,3) at least two, and on average 14 biological replicates were obtained. Because the effect of carbon was consistent accross the two strains totalizing 5 replicates, we estimated that the sample size was sufficient. Network efficiency, cost, and spore data (Fig. 4, Ext. Data Fig. 8, Fig. 5c Inset) were from 12 biological replicates. Density dynamics upon wave collisions (Ext. Data Fig. 11) were from 4 biological replicates. The Phosphorous depletion data (Fig. 3d and Ext. Data Fig. 6) were obtained from 11 biological replicates. That number was chosen to obtain approximately 4 biological replicates per timepoint. Intraradical colonization data (Ext. Data Fig. 7) were from a minimum of 3 biological replicates per time point. For video analysis, the rationale for the choice of biological repliate was similar to the one of network analysis and we tried to have at least 4 networks per treatment to account for biological variability. In a given network, the number of videos depended on the total size of the network. When sampling along a hypha we sample once every millimeter and sample 2 to 3 main hyphae. Bootstrap estimates and errorbar shown within the figure testify that this sampling procedure was sufficient. Average and maximum flow velocity data (Fig. 5b,c) were from 1600 videos acquired across 28 biological replicates. Correlations between velocity and d_tip (Fig. 5e) and between velocity and BC (Fig. 5f) were confirmed robust across 7 biological replicates (Ext Data Fig. 9). Flow velocity data for myristate-grown networks (Ext. Data Fig. 13) were from 7 biological replicates. Figure panels illustrating experimental strategy (Fig. 1), traveling-wave phenomenology (Fig. 2), model validation strategy (Fig. 3b,c, Ext. Data Figs. 15,17), graph betweenness centrality (Fig. 4a), kymograph analysis (Fig. 5a, Ext. Data Fig. 12), and intraradical imaging (Ext Data Fig. 14), each used data from a single representative

	biological replicate. Clarity and conciseness drove the choice of showing only one replicate in those plots to illustrate the experimmental strategy/phenomenology.
Data exclusions	No samples werer excluded from analysis, with the exception of split-sample plates that met any of the the following 3 criteria: (1) Failure to grow more than 1cm of total network length in the fungal compartment. Such samples represented unhealthy AMF colonies, resulting from contamination, inoculation errors and/or failure of host root colonization (30% of prepared samples). More rarely (5% of prepared samples) further growth did not occur due to accidental network detachment from the host root during petri dish handling. (2) Visible contamination before the end of the observation period (45 days). This was a pre-established criterion, and a rare event (5% of prepared sample). (3) Poor image quality that precludes automatic network extraction. This was due to inadequate image focus or a large sampling period (more than one day) due to mechanical failure of the imaging setup. This was a pre-established criterion. Improvements in our robotic imaging setup have made such failures quite rare, but in the early phase of this work, up to 50% samples had to be discarded for this reason.
Replication	Experimental findings were successfully reproduced by employing multiple biological replicates for each strain/species tested totalizing a number of 23 replicates. (Fig. 4, Extended Data Figs. 1,4,5,8,10). The Phosphorous depletion data (Fig. 3d and Ext. Data Fig. 6) were obtained from 11 biological replicates. Average and maximum flow velocity data (Fig. 5b,c Extended Data Fig. 9) were from 1600 videos acquired across 28 biological replicates. Intraradical colonization data (Ext. Data Fig. 7) were from a minimum of 3 biological replicates per time point totalizing a number of 24 replicates). In the case of myristate data (Ext. Data Fig. 13), 7 replicates of networks grown in non-symbiotic context with myristate were used.
Randomization	We did not use randomization. This was not applicable to our study since its focus was on individual colony morphogenesis more than comparison between treatments.
Blinding	We did not use blinding when performing the experiments since the regular handling of experiments required experimenters to be aware of the samples they were managing.

Reporting for specific materials, systems and methods

We require information from authors about some types of materials, experimental systems and methods used in many studies. Here, indicate whether each material, system or method listed is relevant to your study. If you are not sure if a list item applies to your research, read the appropriate section before selecting a response.

Materials & experimental systems

n/a	Involved in the study
☒	☐ Antibodies
☐	☒ Eukaryotic cell lines
☒	☐ Palaeontology and archaeology
☒	☐ Animals and other organisms
☒	☐ Clinical data
☒	☐ Dual use research of concern
☐	☒ Plants

Methods

n/a	Involved in the study
☒	☐ ChIP-seq
☒	☐ Flow cytometry
☒	☐ MRI-based neuroimaging

Eukaryotic cell lines

Policy information about cell lines and Sex and Gender in Research

Cell line source(s)	The R. irregularis strains were isolated from Hausweid, Tänikon, Switzerland (2004) https://doi.org/10.1073/pnas.0306441101 and have been maintain in monoxenic in-vitro cultures with (Ri) T-DNA transformed root organ cultures since then. The strains have been submitted to the Canadian Collection of Arbuscular mycorrhizal Fungi (CCAMF).
Authentication	The strains have been authenticated by the Canadian Collection of Arbuscular mycorrhizal Fungi (CCAMF) and have unique DAOM identifiers
Mycoplasma contamination	The strains have been maintained in monoxenic in-vitro cultures and are not contaminated by Mycoplasma.
Commonly misidentified lines (See ICLAC register)	N.A.

Dual use research of concern

Policy information about dual use research of concern

Hazards

Could the accidental, deliberate or reckless misuse of agents or technologies generated in the work, or the application of information presented in the manuscript, pose a threat to:

No	Yes	
☒	☐	Public health
☒	☐	National security
☒	☐	Crops and/or livestock
☒	☐	Ecosystems
☒	☐	Any other significant area

Experiments of concern

Does the work involve any of these experiments of concern:

No	Yes	
☒	☐	Demonstrate how to render a vaccine ineffective
☒	☐	Confer resistance to therapeutically useful antibiotics or antiviral agents
☒	☐	Enhance the virulence of a pathogen or render a nonpathogen virulent
☒	☐	Increase transmissibility of a pathogen
☒	☐	Alter the host range of a pathogen
☒	☐	Enable evasion of diagnostic/detection modalities
☒	☐	Enable the weaponization of a biological agent or toxin
☒	☐	Any other potentially harmful combination of experiments and agents

Plants

Seed stocks

N.A.

Novel plant genotypes

N.A.

Authentication

N.A.
